# Nb_12_^+^—niobespherene: a full-metal hollow-cage cluster with superatomic stability and resistance to CO attack

**DOI:** 10.1093/nsr/nwac197

**Published:** 2022-09-22

**Authors:** Benben Huang, Hanyu Zhang, Wen Gan, Mengzhou Yang, Zhixun Luo, Jiannian Yao

**Affiliations:** Beijing National Laboratory of Molecular Sciences (BNLMS), State Key Laboratory for Structural Chemistry of Unstable and Stable Species, Institute of Chemistry, Chinese Academy of Sciences, Beijing 100190, China; School of Chemical Science, University of Chinese Academy of Sciences, Beijing 100049, China; Beijing National Laboratory of Molecular Sciences (BNLMS), State Key Laboratory for Structural Chemistry of Unstable and Stable Species, Institute of Chemistry, Chinese Academy of Sciences, Beijing 100190, China; Beijing National Laboratory of Molecular Sciences (BNLMS), State Key Laboratory for Structural Chemistry of Unstable and Stable Species, Institute of Chemistry, Chinese Academy of Sciences, Beijing 100190, China; School of Chemical Science, University of Chinese Academy of Sciences, Beijing 100049, China; Beijing National Laboratory of Molecular Sciences (BNLMS), State Key Laboratory for Structural Chemistry of Unstable and Stable Species, Institute of Chemistry, Chinese Academy of Sciences, Beijing 100190, China; Beijing National Laboratory of Molecular Sciences (BNLMS), State Key Laboratory for Structural Chemistry of Unstable and Stable Species, Institute of Chemistry, Chinese Academy of Sciences, Beijing 100190, China; School of Chemical Science, University of Chinese Academy of Sciences, Beijing 100049, China; Beijing National Laboratory of Molecular Sciences (BNLMS), State Key Laboratory for Structural Chemistry of Unstable and Stable Species, Institute of Chemistry, Chinese Academy of Sciences, Beijing 100190, China; Key Laboratory of Photochemistry, Institute of Chemistry, Chinese Academy of Sciences, Beijing 100190, China

**Keywords:** niobespherene, superatom, cage aromaticity, CO tolerance, cluster materials

## Abstract

Why one chemical is more stable than another is not always easy to understand. A unified answer for metal clusters has led to the establishment of the superatom concept, which rationalizes the delocalization of electrons; however, cluster stability based on superatom theory has not been confirmed unambiguously for any metal other than the s- and p-blocks of the periodic table of elements. Here, we have prepared pure niobium clusters and observed their reactions with CO under sufficient gas collision conditions. We find prominent inertness of Nb_12_^+^, which survives CO attack. Comprehensive theoretical calculation results reveal that the inertness of Nb_12_^+^ is associated with its cage structure and well-organized superatomic orbitals, giving rise to energetic superiority among the studied clusters. It is revealed that not only the 5s but also the 4d electrons of Nb delocalize in the cluster and significantly contribute to the superatomic state, resulting in reasonable cage aromaticity. This hollow-cage cluster, which we have called a ‘niobespherene’, provides a clue with regard to designing new materials of all-metal aromaticity and Nb-involved catalysts free of CO poisoning.

## Teaser

The reactions of niobium clusters with carbon monoxide find a CO-tolerent full-metal cluster Nb_12_^+^ (named niobespherene) which exhibits prominent stability and cage aromaticity and is one of ideal carriers for new catalysts.

## INTRODUCTION

Niobium has been used as a catalytic aid or a carrier in various reactions [1–3], including the selective production of arenes, hydrogen evolution and cycling, sustainable production of fuels, and energy conversion processes [4–8]. For these applications, the stability of the niobium component is important for maintaining the catalytic performance, but challenges remain, as niobium ([Kr]4d^4^5s^1^) is highly reactive with various chemicals. For example, niobium readily coordinates with carbon monoxide, which is an important but toxic reduction gas used in the metallurgical industry and is also the main component of syngas. There has been much research interest in the adsorption mechanism of CO on diverse metals [9–12] and in new catalysts that can convert and remove toxic CO; insights have been gained regarding the chemical bonding in transition metal (TM) carbonyl clusters [13]. However, a pending question remains on how to rationally design CO-tolerant [14–17] metal catalysts for highly efficient chemo-selective reactions.

Metal clusters bridge the gap between atoms and macroscopic materials, enabling us to reveal the chemical mechanisms involved in the aforementioned processes. Stable metal clusters are often determined through a reaction with oxygen, and their stabilities are reinforced at electronic/geometric shell closures based on the nearly free electron gas theory of metals and the jellium model [18,19], such as the findings of spheroidal Al_13_^−^ [20], Ag_17_^−^ [21] and Au_55_ [22]. The spheroidal structure was also addressed for a series of heteroatom-doped clusters [23–25] enabling the optimal packing of atoms and tuneable valence electrons. Previous studies have also predicted the high stability of a few hollow-cage metal and non-metal clusters, such as Au_16_^−^, Au_32_ and C_60_ [26–28]. Also illustrated is that the radial extension of the outermost d-electrons of TMs contributes to electronic delocalization, showing superatomic features [29–33]. However, it is challenging to fully understand how the d-bands construct superatom orbitals and how superatom states behave in the gas-phase reactions of TM clusters.

With one atom making a difference, the stability and the reactivity of metal clusters are highly dependent on the size and geometric/electronic structures. For example, the reactivities of Al_12_^−^ with various chemicals were found to be in sharp contrast to the high stability of Al_13_^−^ [34]. Additionally, the reactivity of oxygen reduction on Pt_12_ and Pt_13_ clusters embodies significantly different catalysis [35]. For niobium, previous studies have observed the altered relative inertness of Nb_8_, Nb_10_^+/0^ and Nb_12_^+/0^ in gas-phase reactions [36–39]. However, a parallel comparison of the stability and reaction mechanisms of pure niobium clusters is limited. It is significantly challenging to identify a magic number of Nb*_n_*^+^ clusters and strictly differentiate the size-dependent cluster stability and reactivity.

Here, we report a study of rich-pressure reactions of Nb*_n_*^+^ with CO by a customized multi-ion laminar flow tube reactor in tandem with a triple quadrupole mass spectrometer (MIFT-TQMS). As a result, Nb_12_^+^ was found to be inert in the reaction with CO, which is in sharp contrast to its neighbouring clusters. Nb_12_^+^ was also found to be inert in reactions with N_2_ and C_2_H_4_. First-principles density functional theory (DFT) calculations demonstrate that the cluster stability of Nb_12_^+^ is associated with its slightly distorted icosahedral cage structure and superatomic orbitals involving both contributions from the 5s and 4d electrons. We named this aromatic full-metal hollow-cage niobium cluster ‘niobespherene’ and propose CO-tolerant Nb_12_^+^ as a promising candidate for designing new materials and catalysts.

## RESULTS AND DISCUSSION

### Mass spectrometry

**Figure 1.**
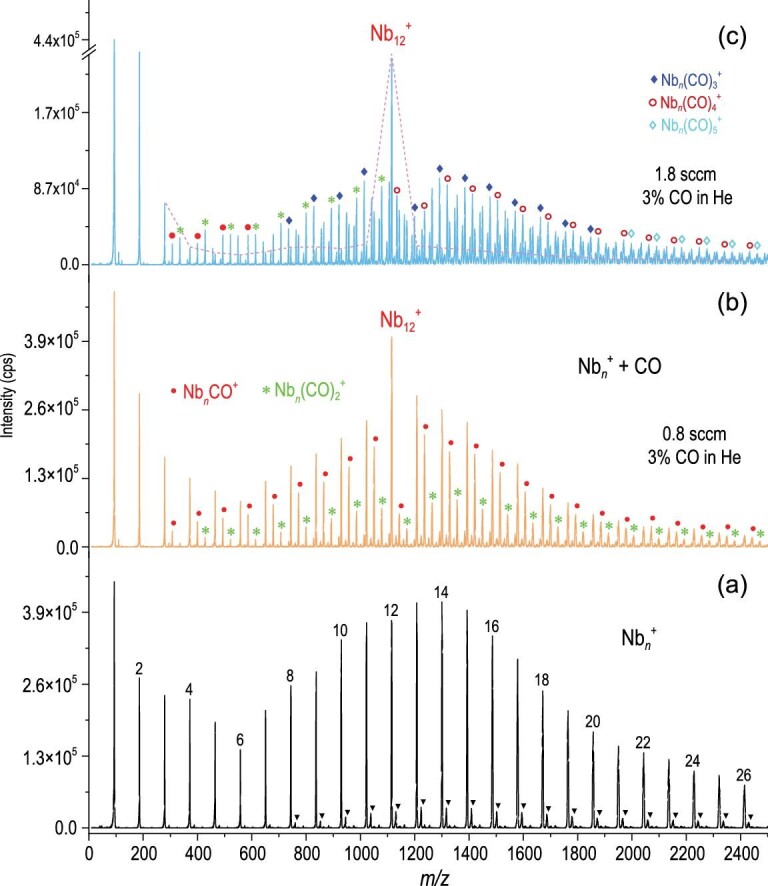
(a) A typical mass spectrum of the cationic niobium clusters produced by a homemade magnetron sputtering (MagS) source and collected by a customized triple quadrupole mass spectrometer (TQMS). (b and c) Mass spectra in the presence of 0.8 sccm and 1.8 sccm reactants (3% CO in He) respectively. The purple dashed line in (c) refers to the remaining pure cationic niobium clusters after the reaction. The unit of mass peak intensity is counts per second (i.e. cps).

Figure 1 presents the mass spectra of Nb*_n_*^+^ (n = 1–26) before and after reacting with CO (for more details see Figs S3–10), where the clusters Nb*_n_*^+^ with n ≥ 3 all generate multiple CO adsorption products and the number of adsorbed CO molecules increases progressively with the rising flow rate of the CO reactant gas. The larger the size of the Nb*_n_*^+^ cluster, the more reactive sites available for CO adsorption. In the Nb*_n_*^+^ (n ≥ 3) series, Nb_12_^+^ is prominently inert with dramatically fewer Nb*_n_*(CO)*_m_*^+^ products than other Nb clusters of similar sizes. With an increasing gas flow rate of CO, Nb_12_^+^ becomes more dominant in the mass spectrum, in sharp contrast to the rest of the pure clusters, which are almost depleted in the reaction (Fig. 1c). Repeated reaction experiments with different distributions of Nb*_n_*^+^ clusters, different reaction times and varying CO concentrations, all conclude that *Nb*_12_^+^ is prominently inert. It is worth mentioning that neither the cation Nb^+^ nor the dimer Nb_2_^+^ reacts with CO, likely because the collisions in the high-pressure flow tube allow vibrationally excited transients to undergo rapid desorption in parallel with thermalization by He collisions that remove excess energy. This is consistent with previous similar studies [40–43]. Also, there are unmountable transition state energy barriers for dissociative CO adsorption on Nb^+^ and Nb_2_^+^, which is in sharp contrast to that of Nb_3_^+^ (Fig. S41).

**Figure 2.**
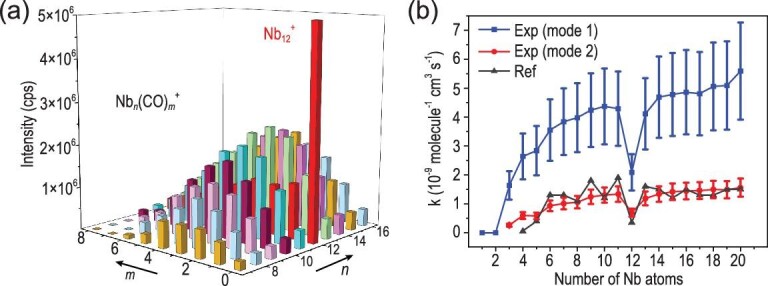
(a) The intensity of Nb*_n_*(CO)*_m_*^+^ (n = 7–16, m = 0–8) when Nb*_n_*^+^ reacts with 1.8 sccm CO reactant (3% in He) after 2.2 ms (Fig. 1c). (b) The reaction rate of Nb_3-20_^+^ is estimated using the quasi first-order reaction equation (mode 1) and Ferguson's measurement method (mode 2) according to the integrated area of Nb*_n_*^+^ and Nb*_n_*(CO)_1–3_^+^ when 1% CO/He reacts with Nb*_n_*^+^ for ∼7.8 ms. Referenced results (black line) are based on the values published by Balteanu *et al*. [46].

Figure 2a shows a histogram of the integral area of the mass spectrum peaks of the Nb*_n_*(CO)*_m_*^+^ products and the residual Nb*_n_*^+^ clusters (n = 7–16, m = 0–8). The mass abundance of the remaining Nb_12_^+^ after the reaction is nearly 10 times larger than that of the other unmodified Nb*_n_*^+^ clusters. Figure 2b plots the reaction rate constants based on Ferguson's method for laminar flow tube reactors [44] (mode 2) in comparison with the quasi first-order reaction rate equation [45] (mode 1) (Figs S11–13 and Table S1). The logarithmic ratio of Nb*_n_*^+^ after and before the reaction, i.e.—ln (I/I_0_), shows a proportional relationship with the gas flow of the CO reactant (Fig. S13), indicating the dominant CO-addition reactions with the niobium clusters (i.e. Nb*_n_*^+^ + m CO **→** Nb*_n_*(CO)*_m_*^+^). Nb_12_^+^ breaks the increasing trend of the reaction rates and corresponds to the local minimum value. This result is consistent with a previous study on charge-dependent CO adsorption on such TM clusters [46]. In addition, the unique inertness of Nb_12_^+^ can also be observed in the reactions with N_2_ and C_2_H_4_ (Figs 14 and 15, and Table S2) and is consistent with the reaction of Nb*_n_*^+^ with other hydrocarbons [37].

### Structure determination

**Figure 3.**
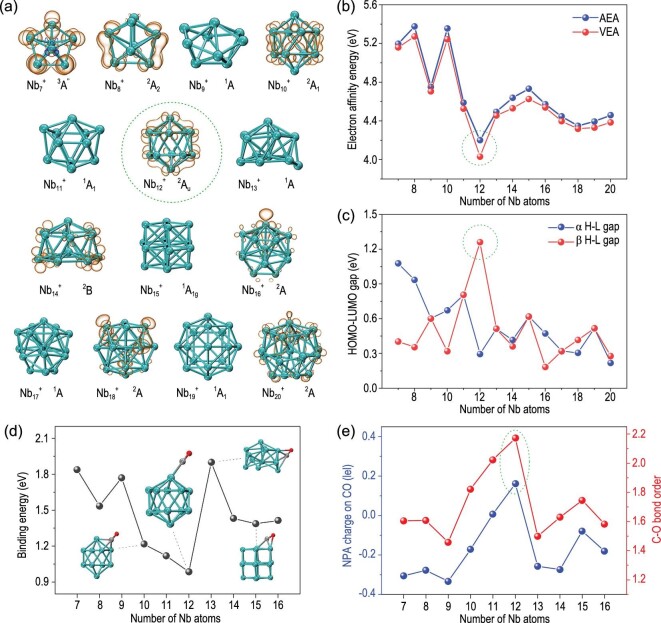
(a) The lowest energy structures of the Nb*_n_*^+^ (n = 7–20) clusters. Electronic states and unpaired electron spin density (an isosurface value at ±0.001 a.u.) are displayed. (b) Adiabatic and vertical electron affinity energies of the Nb*_n_*^+^ clusters. (c) HOMO-LUMO gaps of α and β orbitals of Nb*_n_*^+^. (d) The binding energy of CO on the Nb*_n_*^+^ (n = 7–16) clusters. (e) NPA charge distribution on CO and the C–O bond order in Nb*_n_*CO^+^. All the data are calculated at the BPW91/Lanl2TZ(f) level for Nb*_n_*^+^ and BPW91/6-311G(d) level for CO using the G09 program.

To understand the origin of the inertness of Nb_12_^+^ in reaction with CO, we conducted DFT calculations to determine its chemical stability. Figure 3a displays the lowest energy structures, electronic states and unpaired electron spin densities of the lowest energy isomers of the cationic Nb*_n_*^+^ (n = 7–20) clusters [47,48]. Most of the cationic Nb*_n_*^+^ (n = 7–20) clusters bear symmetric structures, among which Nb_12_^+^ displays a slightly distorted icosahedron structure with an electronic open shell (see Figs S19–23 for natural population analysis of charges, electrostatic surface potential and thermodynamic stability by molecular dynamics simulation). This hollow-cage structure of Nb_12_^+^ is similar to that of Ta_12_^+^ but is different from that of V_12_^+^ (a comparison given in Fig. S32) [49–51]. Similarly, ground-state Nb_10_^+^ also takes the form of a hollow-cage structure (a comparison of Nb_10_^+^ vs. Nb_12_^+^ is given in Figs S33 and S34), which is in agreement with the ground states of V_10_^+^ and Ta_10_^+^ [51]. The Nb_13, 14_^+^ clusters bear an irregular structure containing an Nb core, which is reminiscent of the structure of the Al_13_^+^ cationic cluster [52]. In comparison, the Nb_15-20_^+^ clusters all correspond to core-shell structures containing a mono-niobium core. It is worth mentioning that the lowest energy structures of the anionic and neutral niobium clusters bear differences with the cations. For example, the neutral Nb_12_ could prefer an asymmetry character with a salient dipole moment [53], which concurs with the low-temperature experiments [54]; but the similar isomer of the Nb_12_^+^ cation is much higher in energy than the slightly distorted icosahedron (Figs S16 and S32).

Figure 3b shows the electron affinity energies of the Nb_7-16_^+^ clusters. Interestingly, Nb_12_^+^ is located at the minimum point, indicating that Nb_12_^+^ is the most unfavourable structure for accepting electrons from ligands among all the studied Nb*_n_*^+^ clusters. Figure 3c shows the gaps between the highest-occupied molecular orbital (HOMO) and lowest-unoccupied molecular orbital (LUMO) of the α and β orbitals of Nb_7-16_^+^. Interestingly, the β HOMO-LUMO gap of Nb_12_^+^ is as high as 1.26 eV, which is larger than that of any other cluster. Previous studies have illustrated that the diversity of CO coordination reactions is essentially associated with the local polarization, ligand-to-metal electronic donation and inverse π backdonation [55–58]. Here, the relatively high energy level of the α-LUMO of Nb_12_^+^ could restrict the donation of electron pairs from the 5σ orbital of the CO ligand; additionally, a large β HOMO-LUMO gap minimizes the electronic backdonation to the vacant 2π* orbitals of CO.

### CO adsorption

Figure 3d presents the thermodynamic energy for a CO molecule adsorbed on the Nb*_n_*^+^ (n = 7–16) clusters. Among the Nb*_n_*CO^+^ clusters (Fig. S35), only Nb_12_^+^ adopts top-site adsorption (namely, μ_1_ state), while all the other clusters adopt bridge-site (μ_2_ state, e.g. Nb_15_^+^) or hollow-site (μ_3_ state, e.g. Nb_10_^+^) adsorption. The essentially different adsorption mode of CO on Nb_12_^+^ results in the minimum binding energy of Nb_12_CO^+^. More details of the bond order and bond lengths are presented in Table S3. It is worth mentioning that even in the presence of multiple CO molecules, the adsorptive products Nb_12_(CO)_2,4_^+^ still adopt a top-site adsorption mode, which contrasts with the hollow-site adsorption of Nb_10_(CO)_2,4_^+^ (Fig. S39). Interestingly, the peak intensities of Nb_11_CO^+^ and Nb_13_CO^+^ are nearly equal in mass spectra, but the binding energies of CO on the Nb_11_^+^ and Nb_13_^+^ are significantly different from each other. It is speculated that the CO-bonding mode, adsorption energy and dissociation energy, as well as the stability of nascent metal clusters, contribute to a balance of the final mass spectrometry observation.

The natural population analysis (NPA) of charge distribution of Nb_1-20_^+^ (Fig. S19) shows that each atom on Nb_12_^+^ has an evenly distributed charge between 0 and 0.1 |e|, while the other cage clusters Nb_7-11_^+^ display a variety of charge distributions, and the Nb_13-20_^+^ clusters display negative charge accumulation on the core atom (−1.2 |e| to −1.6 |e|) and a positive charge on the shell atoms (0.15 |e| to 0.4 |e|). In the presence of CO, the size-dependent charge distribution (Tables S5 and S6) results in variation in the electron transfer between the Nb*_n_*^+^ clusters and CO, giving rise to C–O bond activation to different degrees, as shown in Fig. 3e. Nb_12_CO^+^ possesses the shortest C–O bond length at 1.16 Å and the largest C–O bond order among the studied systems, as well as a minor change in the HOMO-LUMO gap of Nb_12_CO^+^ compared with that of Nb_12_^+^ (Fig. S36). In addition, from the energy decomposition analysis (EDA) and infrared-active vibrational modes before and after CO adsorption (Fig. S40 and Table S7), Nb_12_CO^+^ also shows the minimal ν(C–O) value and the lowest interaction energies (Fig. S44, Tables S9 and S10) among all the studied Nb*_n_*CO^+^ clusters.

### Superatomic features

**Figure 4.**
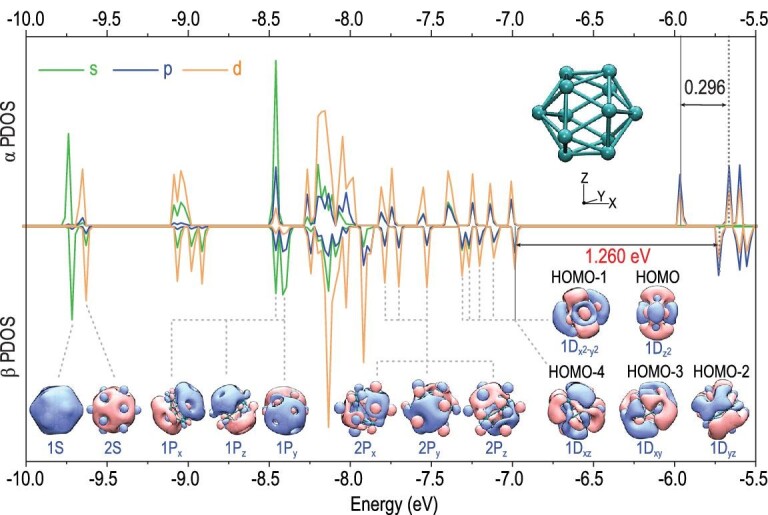
Partial density of states (PDOS) and selected canonical molecular orbitals (CMOs) of the Nb_12_^+^ clusters, calculated at the BPW91/Lanl2TZ(f) level (an isosurface value at ±0.015 a.u.). The pink orbital boundary surface refers to positive isosurface while the blue one refers to negative isosurface. The HOMO-LUMO gap is given in eV (note: there is a broadening of energy levels in plotting the DOS pattern). The green, blue and orange curves indicate the PDOS of shell types s, p and d, respectively.

Nb_12_^+^ possesses a spheroidal icosahedral cage structure and an open electronic shell with 59 valence electrons in total. To fully illustrate its electronic structure, Fig. 4 presents a diagram of the partial density of states (PDOS), where the compositions of the s, p and d shells and the selected canonical molecular orbitals (CMOs) are displayed. It is interesting that the CMOs display remarkable superatomic features with a high degree of electron cloud delocalization throughout the whole cluster. The icosahedral hollow-cage structure and the formation of superatomic orbitals account for the enhanced stability of the Nb_12_^+^ cluster. Through the natural atomic orbital (NAO) method, we analysed the superatomic orbital composition (Tables S11–19). The superatomic 1S orbital mainly originates from the valence 5s orbitals (≥60%) and 4s orbitals (∼24%) of the Nb atoms; however, the main contribution to the superatomic 2S orbital comes from the valence 4d electrons. Similarly, the superatomic 1P orbital originates from the valence 5s shell, while the 2P orbital is formed by the valence 4d electrons of Nb atoms [59]. In brief, there are two sets of superatomic orbitals. One set is 1S^2^|1P^6^ composed of s-electrons, while the other set is 2S^2^|2P^6^|1D^10^ composed of d-electrons.

The superatomic feature results in balanced electron–nuclei interactions, which account for the enhanced stability of Nb_12_^+^. Furthermore, we provide insights into its chemical inertness in reaction with CO. Based on the electronegativities of the atoms (1.6 for niobium and 2.55 for carbon), the Nb–C bonding in these Nb*_n_*CO^+^ clusters is not ionic. To form coordinate-covalent bonds, a proper match, including orbital symmetry and related energy levels of d-state occupation is important [60]. Here, Nb_12_^+^ displays the highest number of unmatched orbital patterns with CO, and the LUMO energy level of Nb_12_^+^ is one of the highest among all the Nb*_n_*^+^ (n = 1–20) clusters (Fig. S27). In addition, when the d-states are located far away from the HOMO energy level of CO, sp–d hybridization is reduced, giving rise to a relatively weak coordinate and smaller adsorption energy. The d-band of Nb_12_^+^ is located far below the α-HOMO energy level with a gap of 1.04 eV calculated at the BPW91/Lanl2TZ(f) level, which is in sharp contrast to the d-bands of the other studied clusters. The distinction of Nb_12_^+^ is also associated with its α-HOMO pattern, which is mainly composed of p-state electrons of the niobium atoms (see PDOS in Fig. 4). In addition, the β-HOMO has a low energy level and there is an unusually large β HOMO-LUMO gap up to 1.26 eV at the BPW91/Lanl2TZ(f) level (a B3LYP method works out at an even larger value of 2.13 eV, Fig. S28).

In addition, we also compared the Kohn-Sham orbital correlation diagrams of Nb_12_CO^+^ and Nb_10_CO^+^ (Fig. S45). The π backdonation in Nb_12_CO^+^ (Nb_12_^+^→CO) is relatively weak, which is only associated with the α-HOMO of Nb_12_^+^ and the vacant 2π* orbital of CO. In contrast, there are relatively strong π backdonation interactions of both α and β orbitals in Nb_10_CO^+^, as well as in the other Nb*_n_*CO^+^ systems. In brief, the experimentally observed relative inertness of Nb_12_^+^ is not only associated with its distinct geometric and electronic stability but also rationalized by the bonding nature and coordination interactions.

### Delocalization of d-orbitals

**Figure 5.**
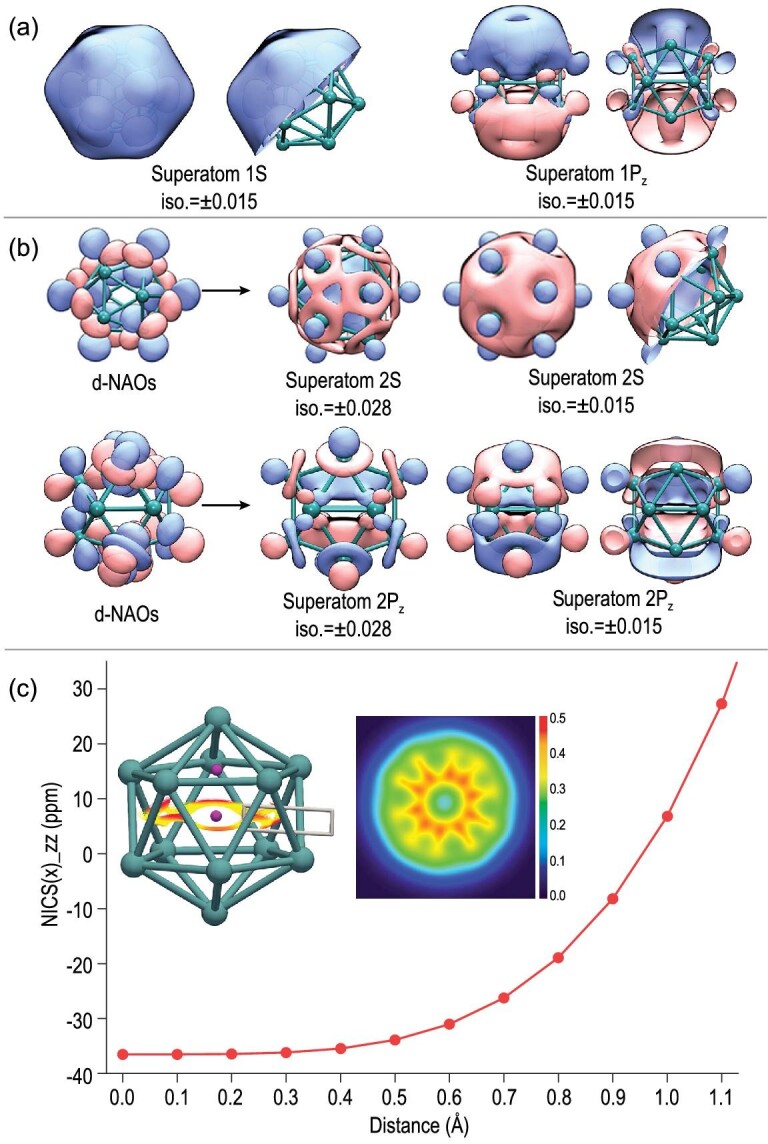
Composition of (a) 1S and 1P_z_ superatomic orbitals and (b) 2S and 2P_z_ superatomic orbitals in Nb_12_^+^ based on the NAO method. The pink orbital boundary surface refers to positive isosurface while the blue refers to negative isosurface. (c) The NICS scan (the start and end positions of the scan are labelled as the purple dot in the cluster structure) of the Nb_12_^+^ cluster. The insets are the stream traces (in their original colours) of the induced ring current of Nb_12_^+^ when an external magnetic field is applied in the [0, 0, 1] direction. The integral of the induced current that crosses the defined section is labelled (white rectangle). The localized orbital locator (LOL) is projected at the XY plane of Nb_12_^+^. An appendix video (.mp4) shows how the d-electrons compose the superatomic orbital.

Figure 5a and b present a comparison of the two sets of superatomic orbitals composed of s- and d-electrons, respectively. It is shown that the d-orbitals of each atom match each other with identical phases to form the superatomic 2S and 2P orbitals that are delocalized throughout the Nb_12_^+^ cluster, giving rise to significant contributions to cluster stability. We also calculated the nucleus-independent chemical shifts (NICSs) at several points along the central axis of a cross section of Nb_12_^+^. As shown in Fig. 5c, the NICS(0)_ZZ value of Nb_12_^+^ is as high as −37.0 ppm in its centre, indicating prominent cage aromaticity [61], which is evidence for the enhanced stability of such clusters. In addition, the analysis of gauge-including magnetically induced current (GIMIC) shows ring current inside the hollow cage when an external magnetic field is applied (insets in Fig. 5c). The GIMIC calculation results show an induced current of 1.13 nA/T in total, with a dominant positive contribution of the β density at 2.91 nA/T and a negative contribution (−1.78 nA/T) to the α density. In this regard, the aromaticity of Nb_12_^+^ is dominated by the β density, which is similar to a recent study on a Pt_10_ cluster [62]. The insets in Fig. 5c also present the integrated electron density using the localized orbital locator (LOL) [63], where the LOL values for hollow-cage Nb_12_^+^ are close to 0.3, indicating distinct electron delocalization in the related region.

### Niobespherene Nb_12_^+^ and Nb_12_^2+^

**Figure 6.**
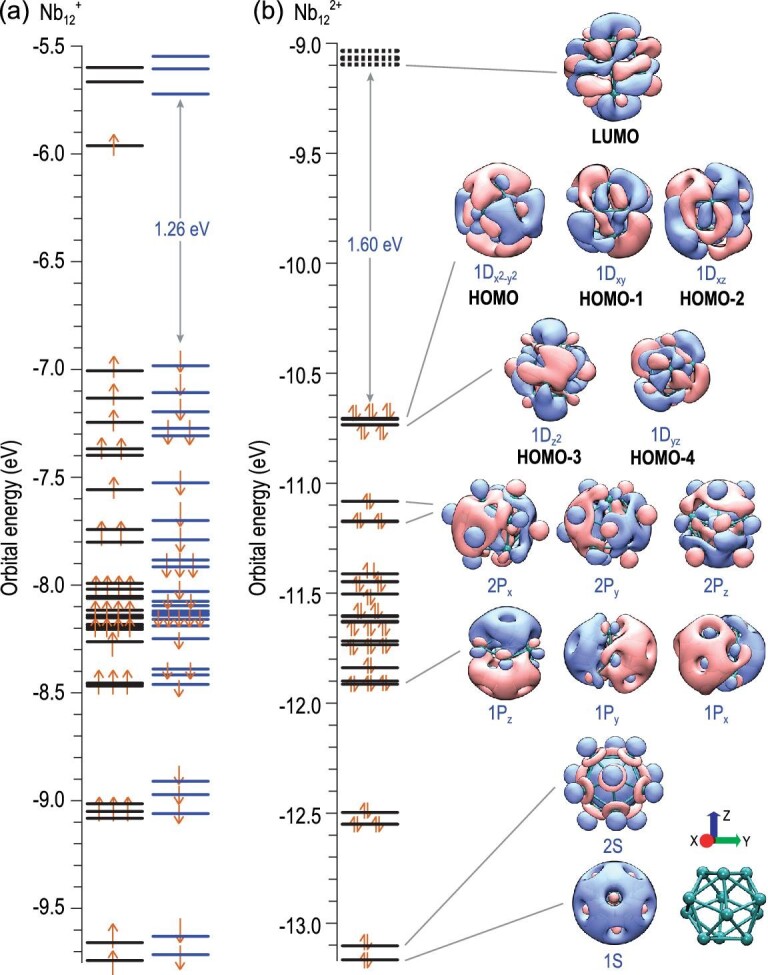
(a) CMO levels of Nb_12_^+^. (b) The CMOs and superatomic features of Nb_12_^2+^ calculated at the BPW91/Lanl2TZ(f) level.

As a comparison, a closed-shell cluster Nb_12_^2+^ with 58 valence electrons has also been studied to fully reveal the superatomic nature of such a cage cluster. As shown in Fig. 6, the orbital energy levels of Nb_12_^2+^ shift downwards as a whole and become more degenerated. It is inferred that the closed-shell Nb_12_^2+^ is more stable than Nb_12_^+^ given the larger HOMO-LUMO gap (1.60 eV by BPW91 level of theory, and even larger at 2.5 eV calculated by B3LYP, Fig. S29), which also leads to inconvenient π backdonation both from the α and β orbitals of Nb_12_^2+^. It is worth mentioning that not all 58 valence electrons delocalize and occupy the superatomic orbitals; instead, only 26 of them embody obvious superatomic features (1S^2^|2S^2^|1P^6^|2P^6^|1D^10^|). The other valence electrons are subject to s-d related hybridization, contributing to the local Nb–Nb bonds thus beneficial to the skeleton stability [59]. We calculated the value of the distortion parameter to evaluate the energy levels in the Clemenger-Nilsson diagram (Fig. S46). As a result, Nb_10_^+^ shows the largest distortion among the three clusters (Nb_12_^+^, Nb_12_^2+^ and Nb_10_^+^), which is associated with fewer valence electrons delocalized in the occupied superatomic orbitals. In comparison, Nb_12_^+^ and Nb_12_^2+^ have a smaller distortion pertaining to the Jahn-Teller effect, and are both associated with 26 electrons (1S^2^|2S^2^|1P^6^|2P^6^|1D^10^|) occupying the frontier superatomic orbitals. In reference to previous studies of cage clusters of Pb_12_^2−^ (‘stannaspherene’) and Sn_12_^2−^ (‘plumbaspherene’) [64], we named the full-metal hollow-cage niobium cluster Nb_12_^+^ ‘niobespherene’ and studied it to better understand its reasonable stability and superatomic nature.

## CONCLUSIONS

A CO-tolerant niobium cluster Nb_12_^+^ was discovered by reacting Nb_n_^+^ with CO in a laminar flow tube in tandem with a customized MIFT-TQMS and a homemade magnetron sputtering (MagS) source. Nb_12_^+^ was found to be inert not only in the reaction with CO but also in reactions with N_2_ and C_2_H_4_. Quantum chemistry calculations revealed that the global minima structure of Nb_12_^+^ corresponds to a slightly distorted icosahedron. This highly symmetric structure gives rise to well-organized superatomic orbitals with the electron shell assigned to 1S^2^|2S^2^|1P^6^|2P^6^|1D^10^. Interestingly, Nb_12_^+^ contains two sets of superatomic orbitals; one set is 1S^2^|1P^6^ and is composed of s-electrons, while the other set is 2S^2^|2P^6^|1D^10^ and is composed of d-electrons. Although the d-orbitals of each atom are anisotropic in space, they match each other and form energy-descent superatomic 2S and 2P orbitals that delocalize throughout the Nb_12_^+^ cluster and balance the geometric and electronic structure, giving rise to enhanced stability. We named this Nb_12_^+^ cluster ‘niobespherene’ and revealed its cage aromaticity with a reasonable induced ring current and an NICS value of −37.0 ppm. We demonstrated the origin of the chemical inertness of Nb_12_^+^ in reaction with CO, which shows top-site adsorption on Nb_12_^+^, corresponding to the smallest binding energy. This is in contrast to the hollow-site and bridge-site adsorption for the other studied clusters. This study helps build an understanding of the formation of superatomic clusters with d-electrons and provides a strategy to design CO-tolerant materials that can prevent the block of catalytic activity caused by CO poisoning.

## METHODS

### Experimental methods

The experiments in this study were conducted on our customized MIFT-TQMS. A self-designed MagS source with a DC power supply of 5 kW was used to obtain the clean mass distributions of small niobium clusters. The experimental details have been provided in the Supplementary Data. The laminar flow tube reactor (60 mm diameter, 1 m long) was maintained at ∼0.9 Torr pressure for laminar flow and sufficient collisional reactions. Different concentrations of CO, N_2_ and C_2_H_4_ (ca., 0.1%–30% in He, 0–10 sccm) were injected into the flow tube from different inlets, corresponding to varying reaction times.

### Theoretical methods

The unbiased global research was conducted by the genetic algorithm (GA) method based on *ab-initio* evolutionary algorithm USPEX (Universal Structure Predictor: Evolutionary Xtallography) [65] combined with Vienna *ab-initio* Simulation Package (VASP) software [66]. All the isomers were checked to make sure that there was no imaginary frequency and the energies were corrected by zero-point vibrations. More details of the calculation methods are provided along with the Supplementary Data.

## Supplementary Material

nwac197_Supplemental_Files
