# Supplementary material for: Nb12+—niobespherene: a full-metal hollow-cage cluster with superatomic stability and resistance to CO attack
Source: Natl Sci Rev. 2022 Sep 22;10(3):nwac197. doi: 10.1093/nsr/nwac197 (PMC10081918; doi:10.1093/nsr/nwac197)
Supplement: nwac197_Supplemental_Files [file nwac197_supplemental_files.zip › Revised_ESI.docx]

***Supporting Information for***

**Nb_12_^+^——****Niobespherene: A** **Full-Metal Hollow Cage Cluster with Superatomic Stability and Resistant to CO Attack**

Benben Huang^#1,2^, Hanyu Zhang^#1^, Wen Gan^1,2^, Mengzhou Yang,^1^ Zhixun Luo*^1,2^ Jiannian Yao^1,3^

**^1^** Beijing National Laboratory of Molecular Sciences (BNLMS), State Key Laboratory for Structural Chemistry of Unstable and Stable Species, Institute of Chemistry, Chinese Academy of Sciences, Beijing 100190, China.

**^2^** School of Chemical Science, University of Chinese Academy of Sciences, Beijing 100049, China.

**^3^** Key Laboratory of Photochemistry, Institute of Chemistry, Chinese Academy of Sciences, Beijing 100190, China.

* Corresponding author. Email: zxluo@iccas.ac.cn

**^#^** These authors contributed equally to this work.

**Contents**

[1. Methods 2](#_Toc86734997)

[1.1 Experimental methods 2](#_Toc86734998)

[1.2 Theoretical methods 2](#_Toc86734999)

[2. Experimental Results and Analysis 4](#_Toc86735000)

[2.1 Argon collision experiment 4](#_Toc86735001)

[2.2 Concentration dependence 5](#_Toc86735002)

[2.3 Reaction time dependence 8](#_Toc86735003)

[2.4 Size distribution dependence 10](#_Toc86735004)

[2.5 Mass abundance analysis 12](#_Toc86735005)

[2.6 Rate constant estimation 14](#_Toc86735006)

[2.7 The reaction of “Nb_n_^+^ + N_2_” 15](#_Toc86735007)

[2.8 The reaction of “Nb_n_^+^ + C_2_H_4_” 16](#_Toc86735008)

[3. Theoretical Calculations on Nb_n_^+^ 19](#_Toc86735009)

[3.1 Global search and structure determination 19](#_Toc86735010)

[3.2 Charge population and electrostatic potential 22](#_Toc86735011)

[3.3 Molecular dynamics simulation 23](#_Toc86735012)

[3.4 PDOS of Nb_n_^+^ clusters 26](#_Toc86735013)

[3.5 Molecule orbitals and NAO analysis 28](#_Toc86735014)

[3.6 HOMO and LUMO energy levels 34](#_Toc86735015)

[3.7 MOs of Nb_n_^+^ at B3LYP/Lanl2TZ(f) Level 35](#_Toc86735016)

[3.8 A comparison of Nb_n_^+^ with V_n_^+^ 39](#_Toc86735017)

[3.9 A comparison of Nb_10_^+^ with Nb_12_^+^ 41](#_Toc86735018)

[4. CO Adsorption 42](#_Toc86735019)

[4.1 Geometric and electronic structures 42](#_Toc86735020)

[4.2 NPA of Nb_10,12_CO^+^ 46](#_Toc86735021)

[4.3 Vibration frequency 48](#_Toc86735022)

[4.4 Reaction coordinates 49](#_Toc86735023)

[4.5 PDOS and NAO of Nb_12,10_CO^+^ 50](#_Toc86735024)

[4.6 Energy decomposition analysis 52](#_Toc86735025)

[4.7 Electron deformation density analysis 55](#_Toc86735026)

[5. Superatomic nature 57](#_Toc86735027)

[References 66](#_Toc86735028)

1. Methods

1.1 Experimental methods

The experiments in this study are conducted on our customized multi-ions laminar flow tube reactor in tandem with a triple quadrupole mass spectrometer (MIFT-TQMS) [1, 2], seen as below. A niobium disk (99.95% purity, 50.8 mm diameter, 5 mm thickness) was used as the sputtering target. High-purity helium (>99.9997%) and high-purity argon (>99.9997%) (Beijing AP BAIF Gases Industry Co., Ltd.,) were used as carrier gas and work gas of sputtering, introduced from the rare of MagS source and the magnetron head respectively, with the gas flow rates controlled by mass flowmeters (Alicat, a range of 0-100 sccm) and adjustable needle valves. Typical parameters for the MagS source to produce the niobium clusters are: ~170 V DC voltage, ~4.5 A current, a background pressure of source chamber at ~7 Torr, He carrier gas at ~15 slm, while argon at 60-120 sccm.


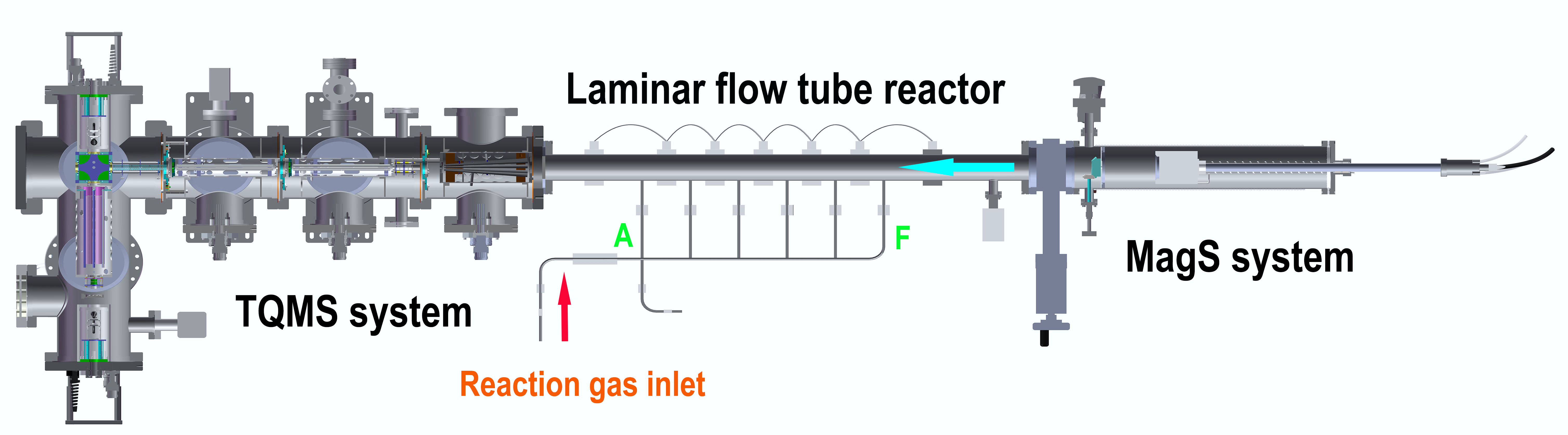


Figure S1 | Instrumentation. A diagram showing the customized MIFT-TQMS instrument. The inlets A to F of the laminar reaction tube correspond to the different reaction time duration for the reactant gas to interact with the metal clusters in the rich-pressure condition.

1.2 Theoretical methods

The lowest-energy structures of all the Nb_n_^+^ are optimized at the BPW91/Lanl2TZ(f) level of theory using the G09 program [3], along with a global energy minimum structure search for chosen structures conducted by the genetic algorithm (GA) method based on the ab initio evolutionary algorithm USPEX (Universal Structure Predictor: Evolutionary Xtallography) [4] implanted in the Vienna ab initio simulation software package (VASP) software [5] (Figs S16-18). Then, the determined structures with relative lower energies, as well as those referred to in previous literature [6-10], were taken as initial guesses and further optimized at the BPW91/Lanl2TZ(f) [11-13] level of theory using G09 grogram [3]. All the isomers were checked to make sure that there is no imaginary frequency and the energies were corrected by zero-point vibrations. Natural population analysis (NPA), Wiberg bond index (WBI), partial density of states (PDOS), natural atomic orbital (NAO) [14, 15] and electron configuration are analysed by using the Multiwfn software [16]. The geometric structures, orbitals, and NPA charge distributions are plotted by visual molecular dynamics (VMD) [17]. Energy decomposition analysis (EDA) and Kohn-Sham orbital correlation diagrams were conducted based on natural orbitals for chemical valence (NOCV), calculated with Amsterdam Density Functional (ADF) program [18, 19] at the PW91/TZP level of theory. The analyses of nucleus-independent chemical shifts (NICS) [20-23] and gauge-including magnetically induced current (GIMIC) were conducted by referring to the previous literature [24-26].

Born–Oppenheimer molecular dynamics (BOMD) simulations were performed for the global minima of Nb_12_^+^, Nb_10_^+^, and Nb_12_^2+^ at different temperatures for 5 ps using the software suite of CP2K [27]. The gauge including magnetically induced current (GIMIC) [24, 25] of Nb_12_^+^ was calculated on the basis of gauge-invariant atomic orbitals (GIAO) [28, 29] method. The open-shell calculation of GIMIC was conducted utilizing the program package of GIMIC [26].

To test the DFT methods, a comparison of binding energies per atom, adiabatic ionization energy, and dissociation energy of the Nb_n_ clusters was conducted, as shown in Fig. S2. Considering that BPW91/Lanl2TZ(f) level of theory finds better consistency with the experimental values, we chose it to describe the energetics of Nb clusters in this study.


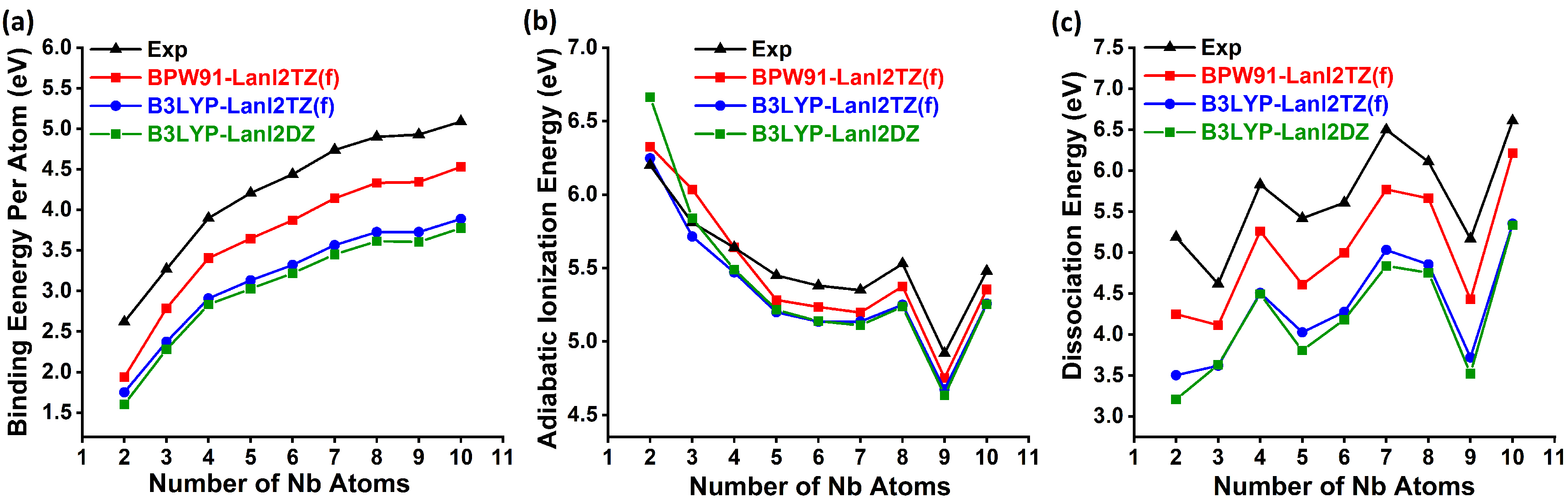


Figure S2 | DFT method test. (a) Binding energies per atom of the Nb_n_ clusters, defined as $\boldsymbol{BE}\left( \boldsymbol{Nb}_{\boldsymbol{n}} \right)\boldsymbol{=}\left[ \boldsymbol{n\times E}\left( \boldsymbol{Nb} \right)\boldsymbol{-E}\boldsymbol{(Nb}_{\boldsymbol{n}}\boldsymbol{)} \right]/\boldsymbol{n}$. (b) Adiabatic ionization energy of the Nb_n_ clusters. c, Dissociation energies of the Nb_n_ clusters, defined as $\boldsymbol{DE}\left( \boldsymbol{Nb}_{\boldsymbol{n}} \right)\boldsymbol{=}\boldsymbol{E}\boldsymbol{(Nb}_{\boldsymbol{n-1}}\boldsymbol{)+E}\left( \boldsymbol{Nb} \right)\boldsymbol{-E}\boldsymbol{(Nb}_{\boldsymbol{n}}\boldsymbol{)}$. The experimental values are taken from refs [6, 9, 30-32].

2. Experimental Results and Analysis

2.1 Argon collision experiment


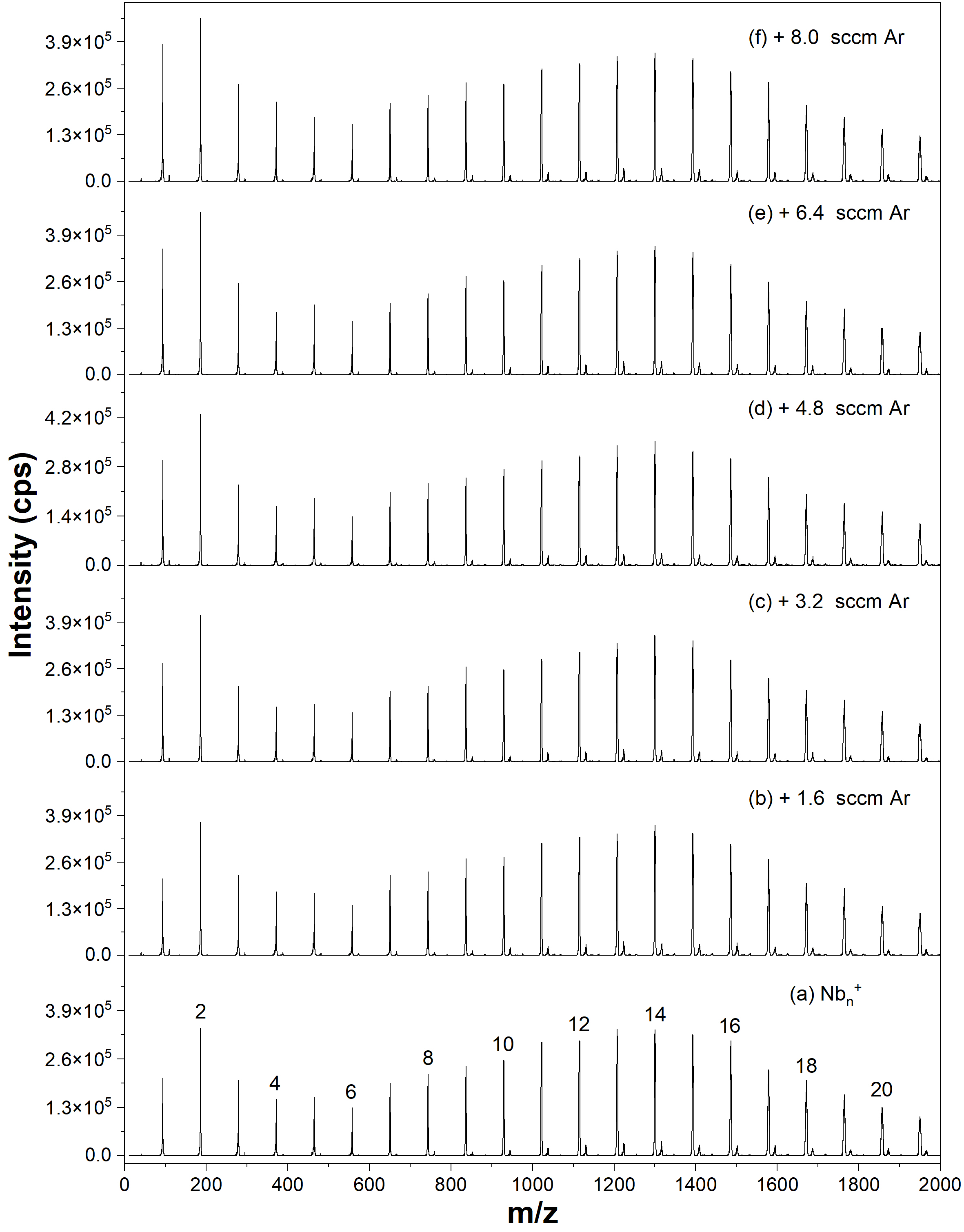


Figure S3 | Exclusion experiments by argon collisions. Mass spectra of (a) cationic niobium clusters and (b-f) after 2.2 ms colliding with high-purity argon gas at a flow rate of 1.6, 3.2, 4.8, 6.4, 8.0 sccm. The unit of intensity is counts per second (cps).

In the collision experiments of Nb_n_^+^ with Ar, the intensity and distribution of the nascent clusters almost don’t change (Fig. S3), indicating a universal size dependence of the Nb_n_^+^ clusters in reacting with CO.

2.2 Concentration dependence

Repeated experiments of the reactions of Nb_n_^+^ with varying CO concentrations all arrive at the same end showing prominent inertness of Nb_12_^+^, displayed in the following Figs S4-6.


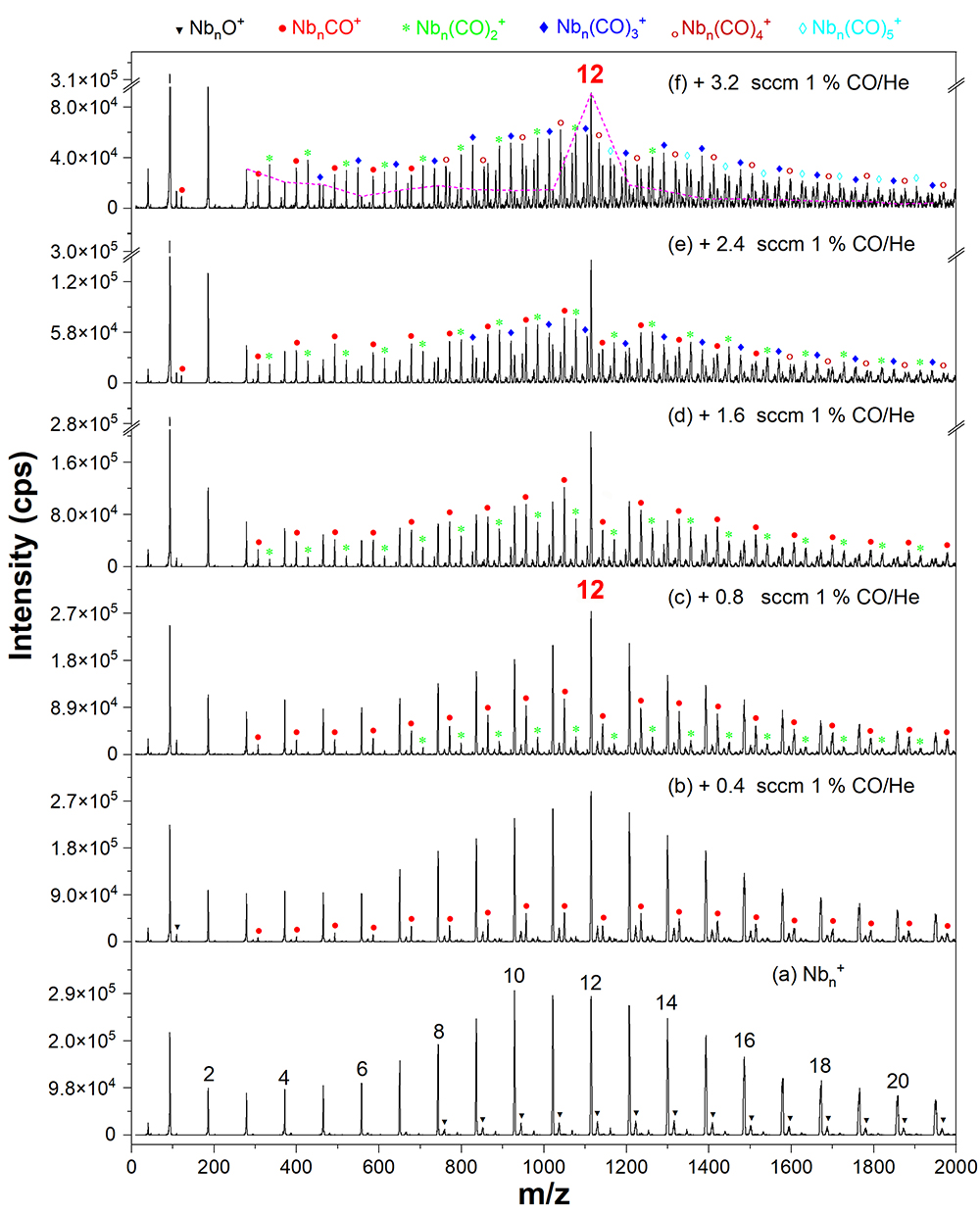


Figure S4 | Reactions with 1% CO/He. Mass spectra of (a) cationic niobium clusters and (b-f) after 7.8 ms reacting with 0.4, 0.8, 1.6, 2.4, 3.2 sccm 1% CO/He. The purple dash line refers to remaining pure cationic niobium clusters after the reaction.


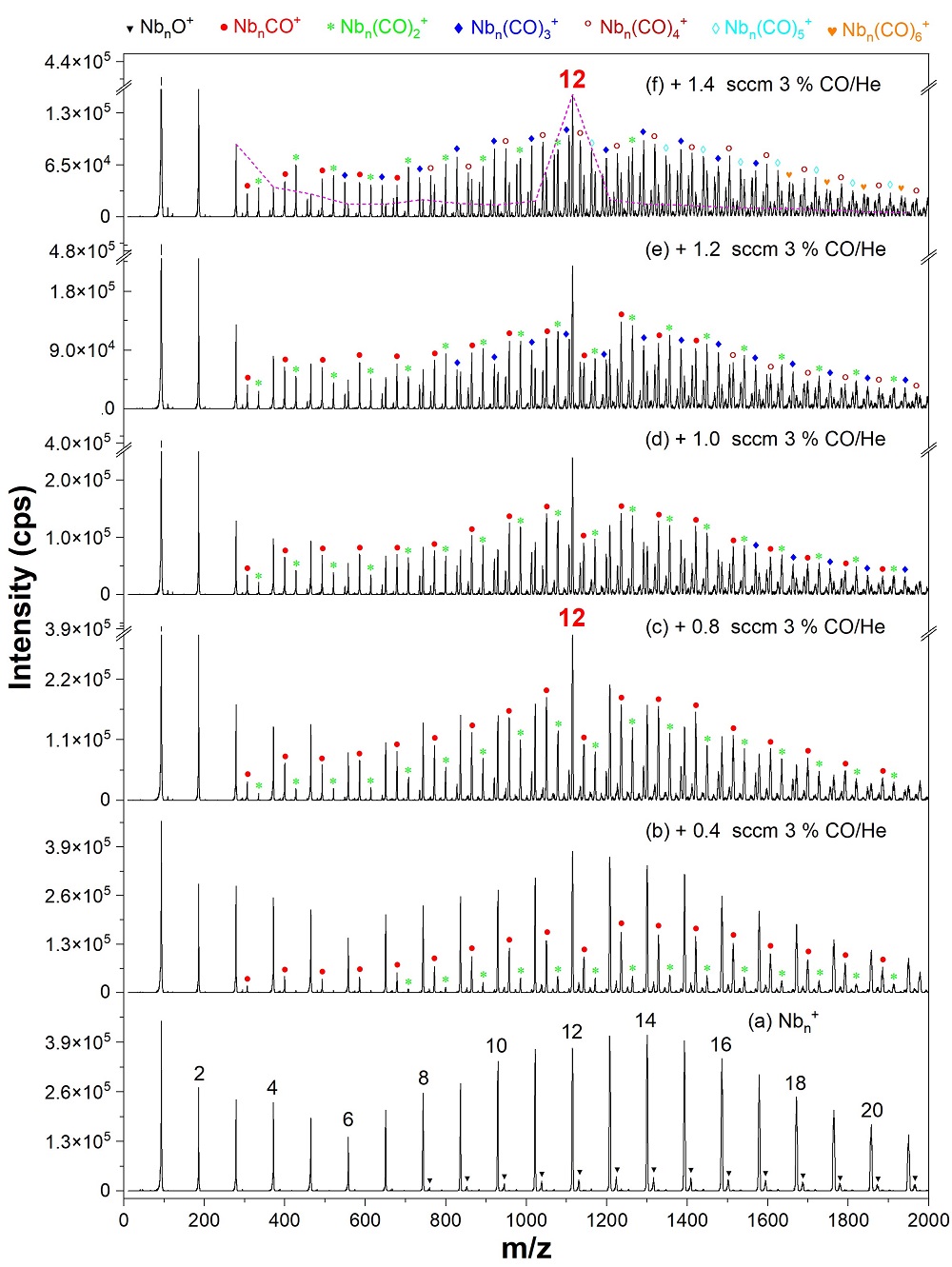


Figure S5 | Reactions with 3% CO/He. Mass spectra of (a) cationic niobium clusters and (b-f) after 7.8 ms reacting with 0.4, 0.8, 1.0, 1.2, 1.4 sccm 3% CO/He. The purple dash line refers to the remaining pure cationic niobium clusters after the reaction.

**
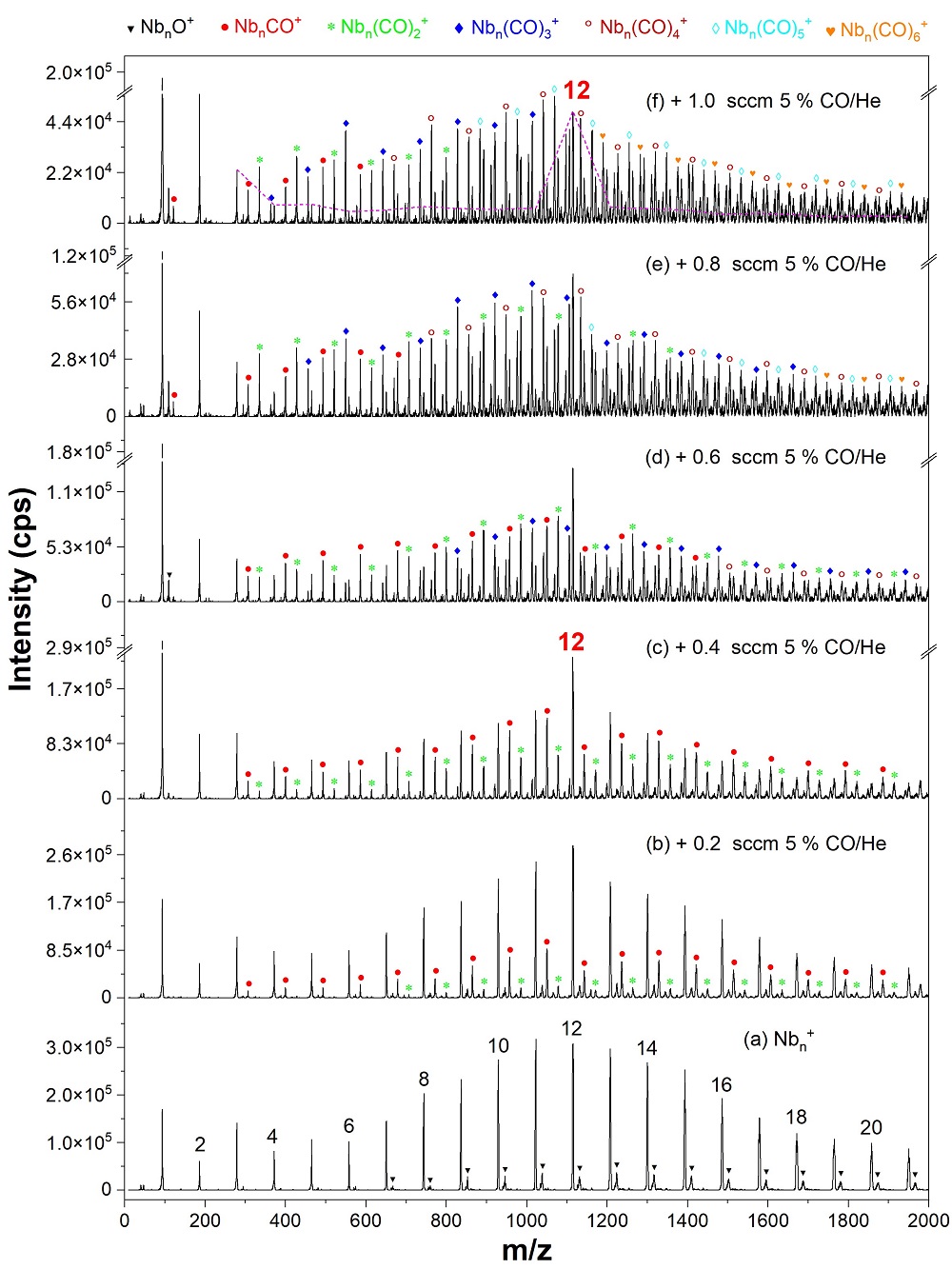
**

Figure S6 | Reactions with 5% CO/He. Mass spectra of (a) cationic niobium clusters and (b-f) after 7.8 ms reacting with 0.2, 0.4, 0.6, 0.8, 1.0 sccm 5% CO/He. The purple dash line refers to the remaining cationic niobium clusters after reactions.

2.3 Reaction time dependence

Repeated experiments of the reactions of Nb_n_^+^ with CO at different duration time also arrive at the same end showing prominent inertness of Nb_12_^+^, as shown in the following Figs. S7-8.


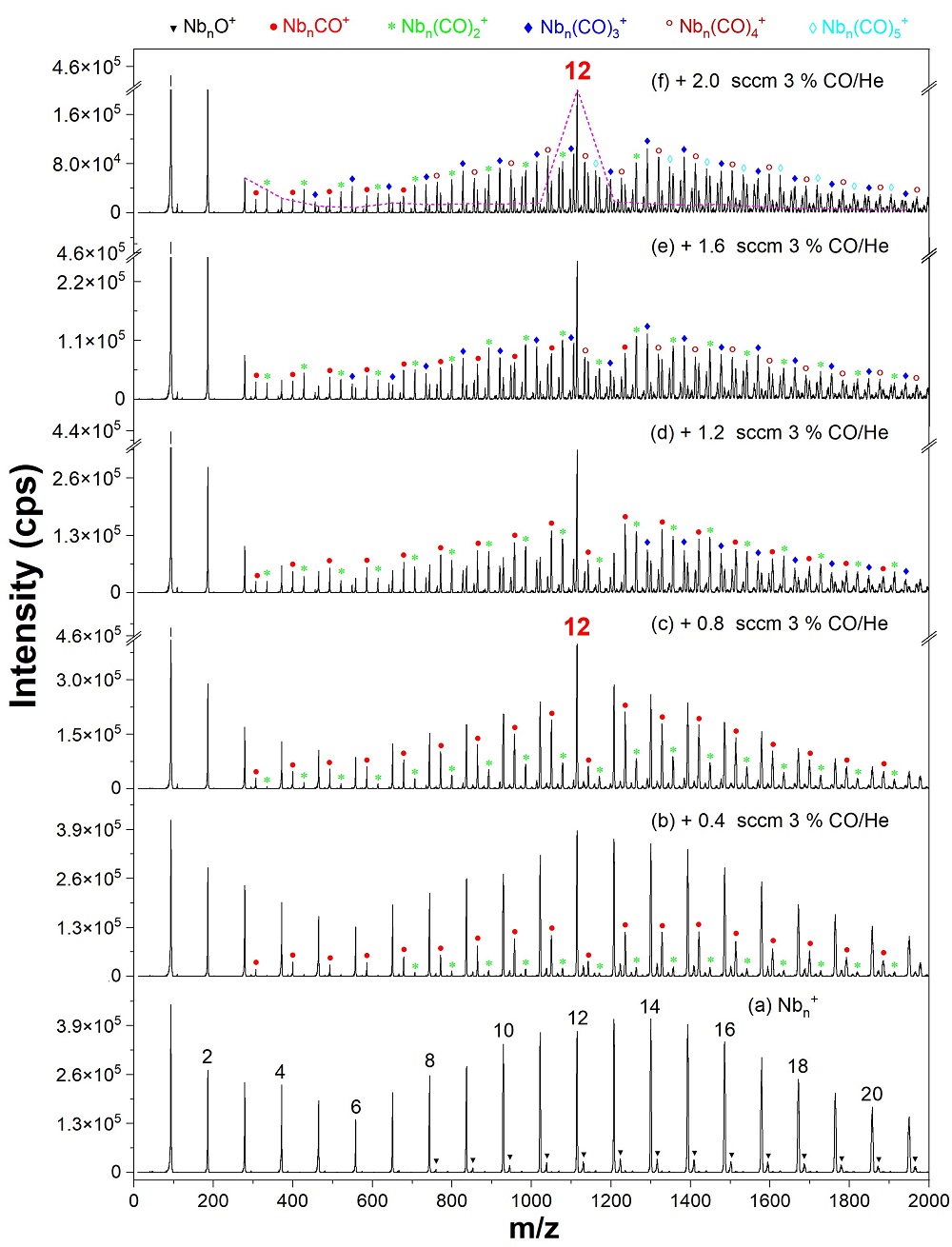


Figure S7 | Reaction time at 2.2 ms for 3% CO in He. Mass spectra of (a) cationic niobium clusters and (b-f) after 2.2 ms reacting with 0.4, 0.8, 1.2, 1.6, 2.0 sccm 3% CO/He. The purple dash line refers to remaining pure cationic niobium clusters after the reaction. The unit of intensity is counts per second.

**
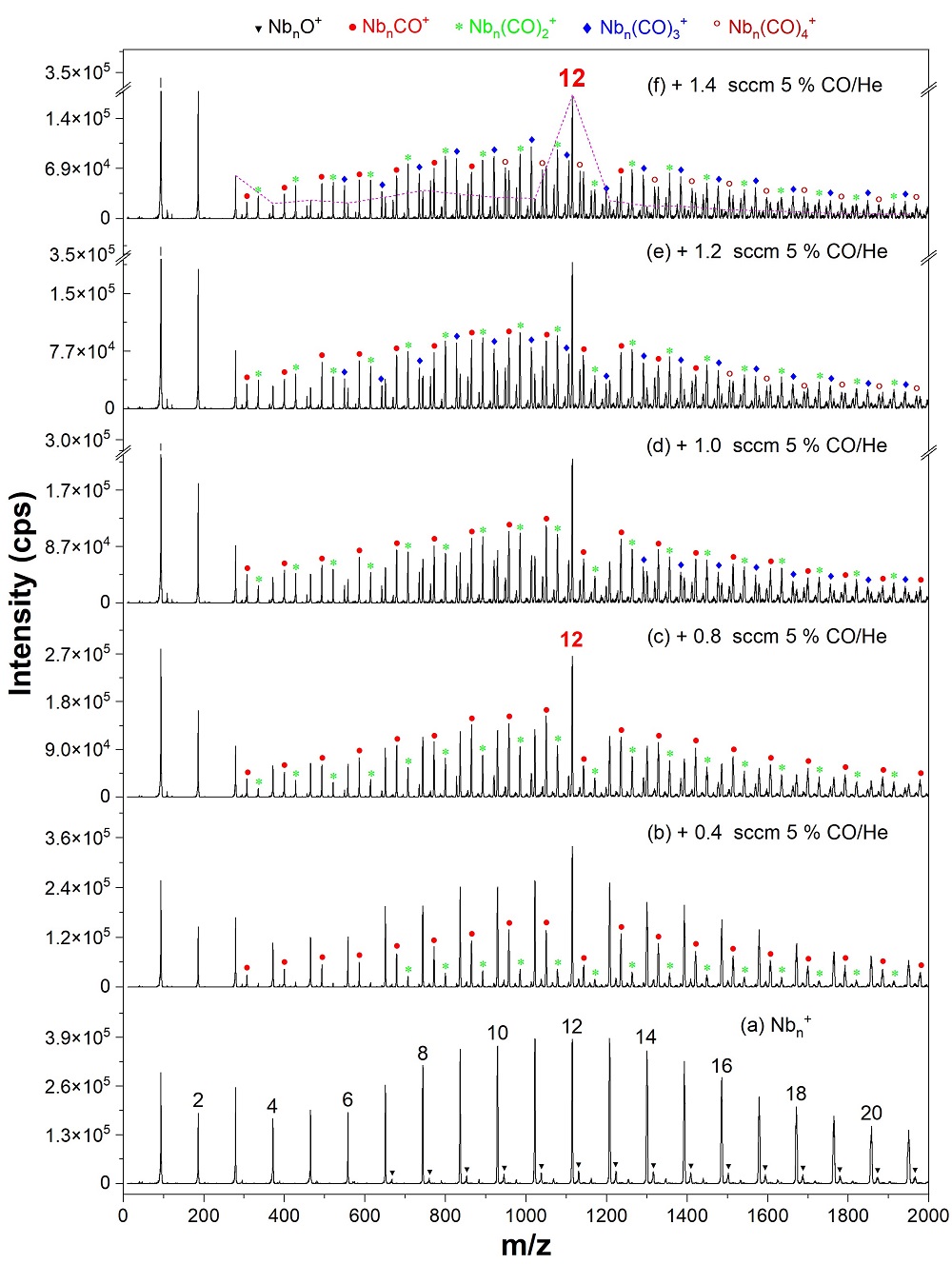
**

Figure S8 | Reaction time at 2.2 ms for 5% CO in He. Mass spectra of (a) cationic niobium clusters and (b-f) after 2.2 ms reacting with 0.4, 0.8, 1.0, 1.2, 1.4 sccm 5% CO in He. The purple dash line refers to remaining pure cationic niobium clusters after the reaction. The unit of intensity is counts per second.

2.4 Size distribution dependence

Repeated experiments of CO reacting with the Nb_n_^+^ at different size distributions also arrive at the same end showing prominent inertness of Nb_12_^+^, as shown in the following Figs. S9-10.


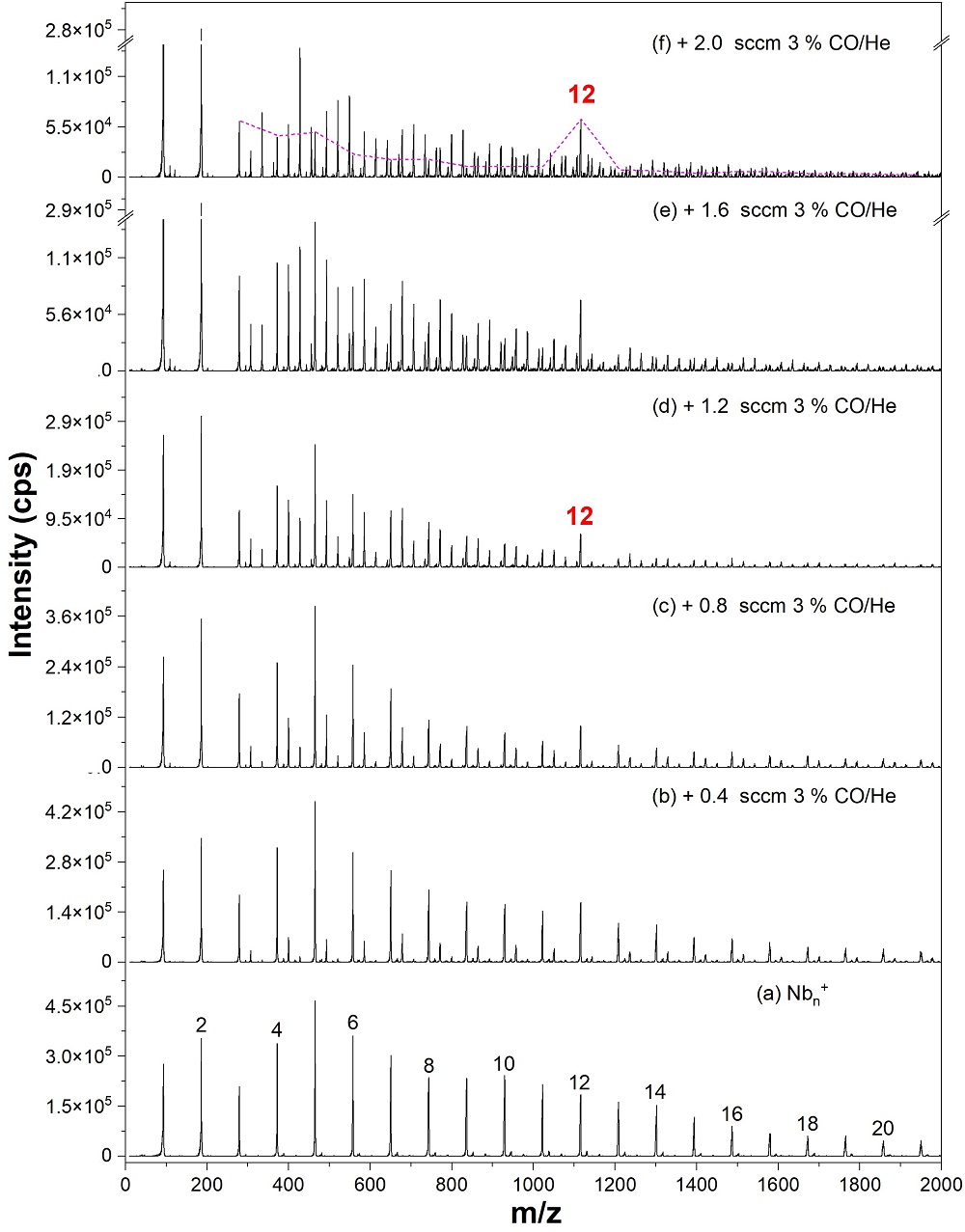


Figure S9 | Repeated experiments with a different size distribution. Mass spectra of (a) another size of distribution of cationic niobium clusters and (b-f) after 2.2 ms reacting with 0.4, 0.8, 1.2, 1.6, 2.0 sccm 3% CO/He. The purple dash line refers to remaining pure cationic niobium clusters after the reaction. The unit of intensity is counts per second.


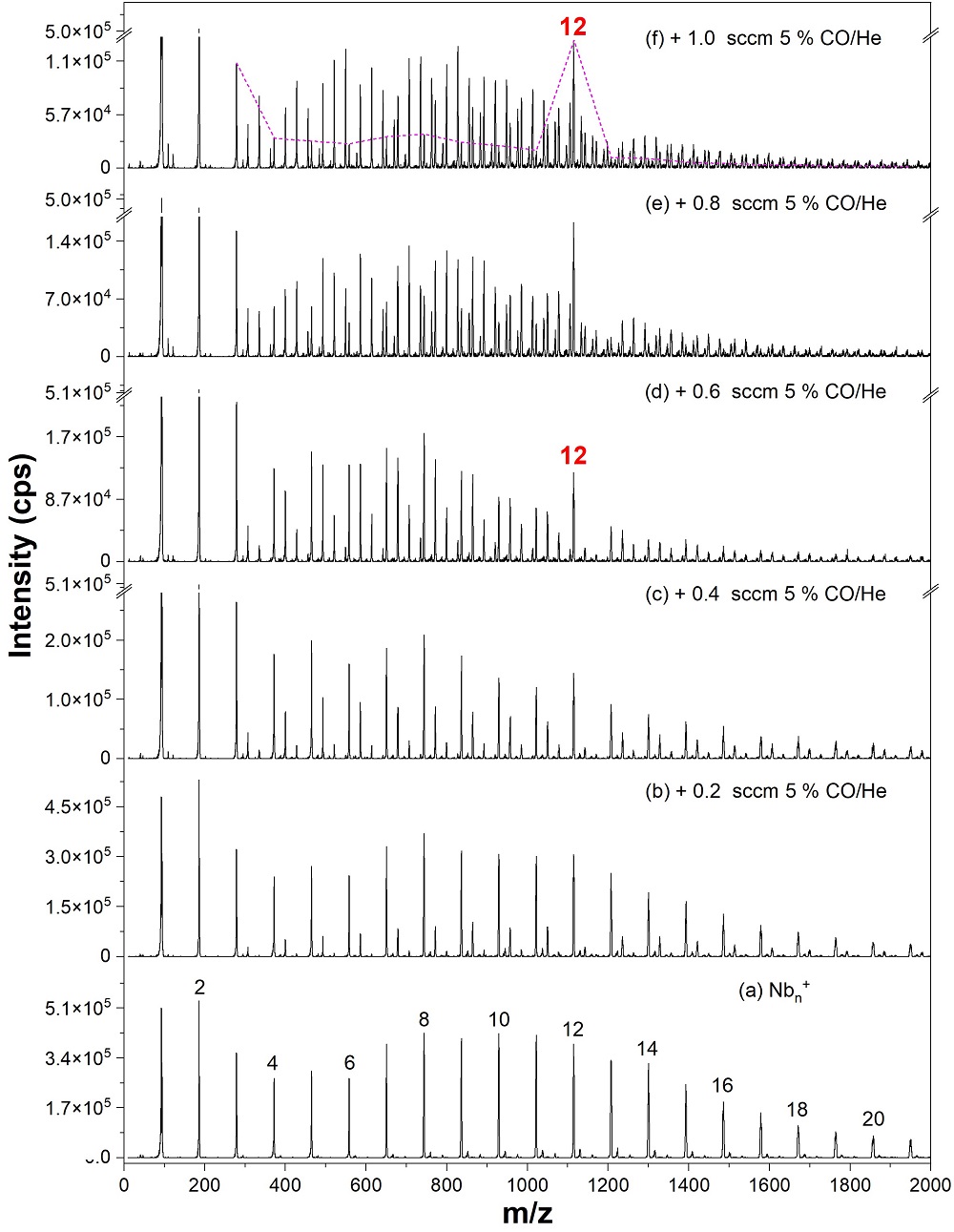


Figure S10 | Repeated experiments with a different size distribution. Mass spectra of (a) another size of distribution of cationic niobium clusters and (b-f) after 2.2 ms reacting with 0.2, 0.4, 0.6 1.8, 1.0 sccm 5% CO in He. The purple dash line refers to remaining pure cationic niobium clusters after the reaction. The unit of intensity is counts per second.

2.5 Mass abundance analysis


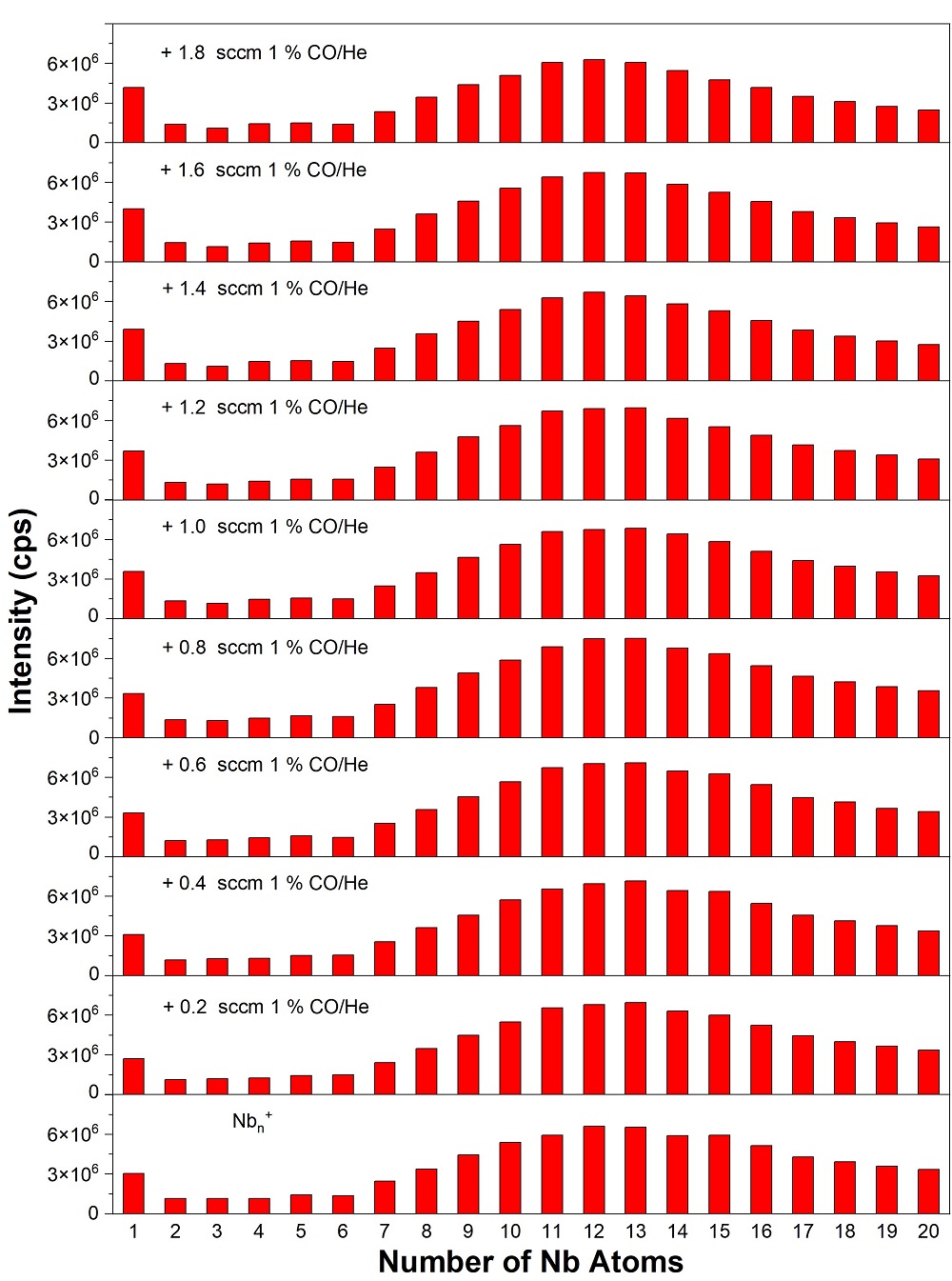


Figure S11 | Mass abundance analysis. The changes in total intensity of Nb_n_^+^ (n=1-20) and their initial products Nb_n_CO^+^, Nb _n_(CO)_2_^+^, Nb_n_(CO)_3_^+^ as the flow of 1% CO in He increases from 0 to 1.8 sccm.


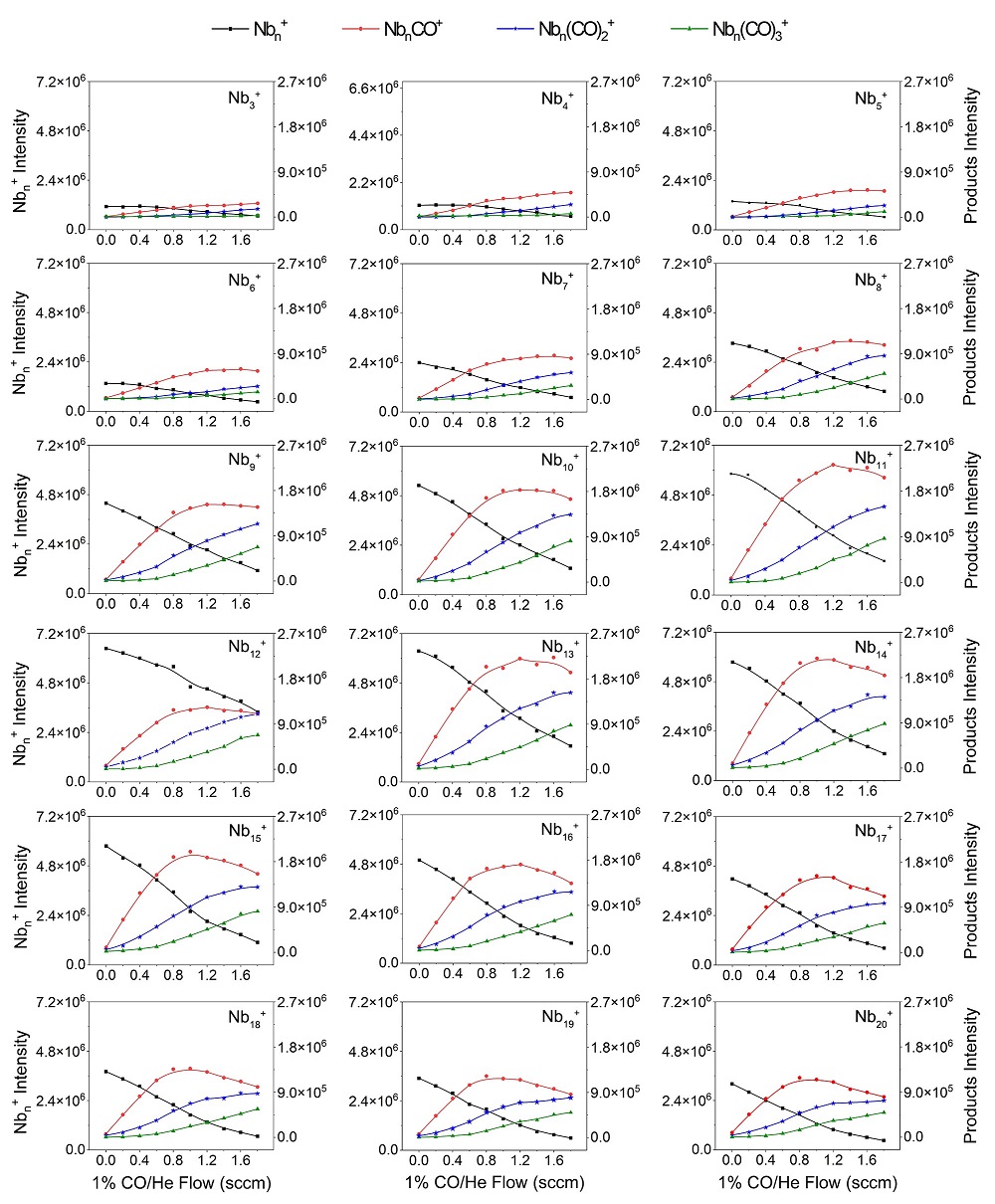


Figure S12 | The changes of mass peak intensities. The changes in intensity of Nb_n_^+^ (n=3-20) and products Nb_n_CO^+^, Nb_n_(CO)_2_^+^, Nb_n_(CO)_3_^+^ vs. the flow (0-1.8 sccm) of the reactant, typically 1% CO in He.

2.6 Rate constant estimation


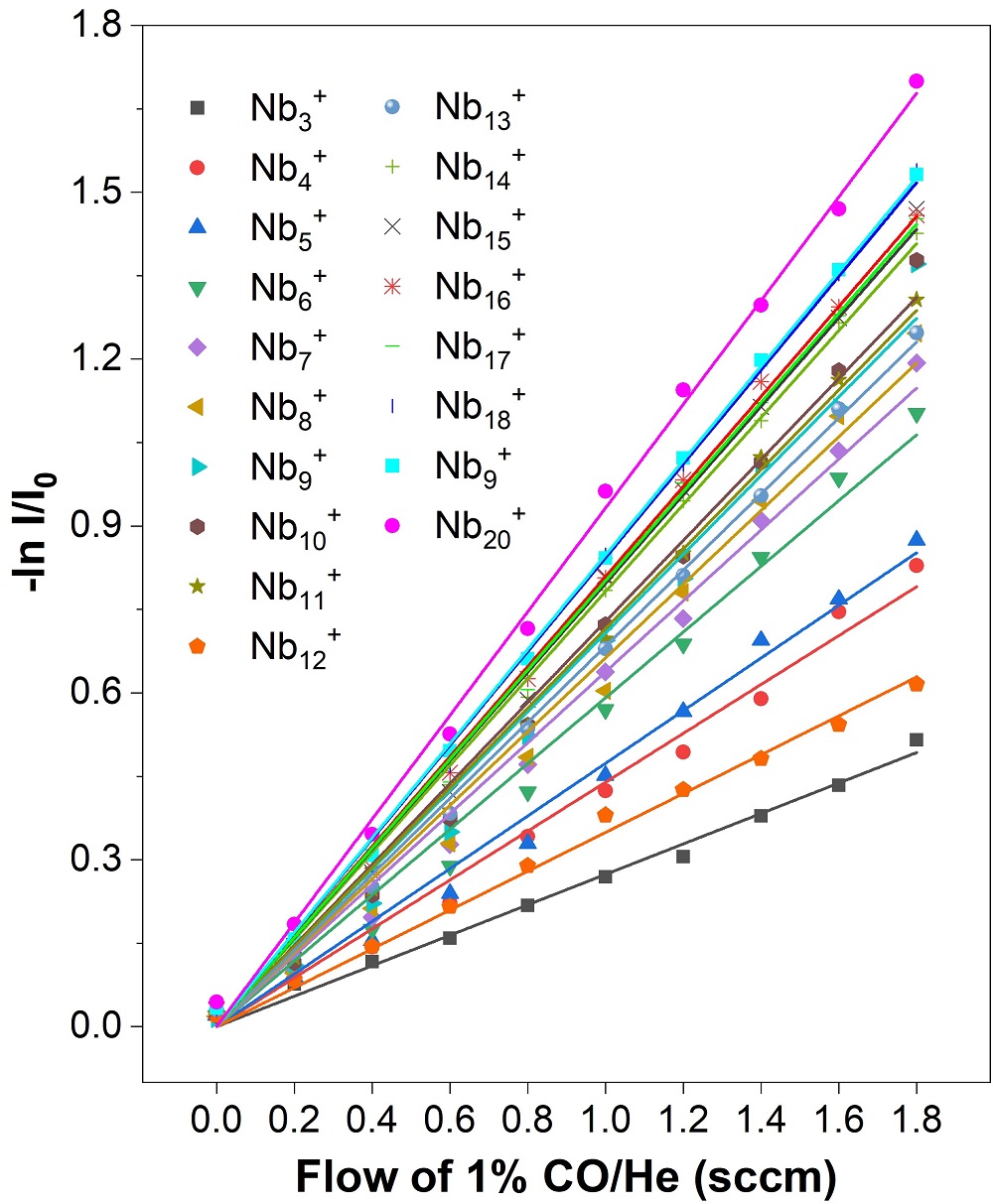


Figure S13 | Relative reaction. Linear fitting of - ln (I/I_0_) of Nb_n_^+^ (n=3-20) vs. the flow (0-1.8 sccm) of 1% CO in He.

Table S1 | The scope of the function of the ln(*I/I_0_*) of Nb_1-20_^+^ with the flow of 1% CO in He and R^2^ showing first-order reactions.

| ***n*** | ***k_scope_*** | ***R^2^*** | ***n*** | ***k_scope_*** | ***R^2^*** |
| --- | --- | --- | --- | --- | --- |
| **1** | - | - | **11** | 0.72 | 0.999 |
| **2** | - | - | **12** | 0.35 | 0.998 |
| **3** | 0.27 | 0.997 | **13** | 0.68 | 0.999 |
| **4** | 0.44 | 0.996 | **14** | 0.78 | 0.999 |
| **5** | 0.47 | 0.997 | **15** | 0.80 | 0.999 |
| **6** | 0.59 | 0.996 | **16** | 0.81 | 0.999 |
| **7** | 0.64 | 0.997 | **17** | 0.80 | 0.999 |
| **8** | 0.66 | 0.996 | **18** | 0.84 | 0.999 |
| **9** | 0.71 | 0.996 | **19** | 0.85 | 0.999 |
| **10** | 0.73 | 0.997 | **20** | 0.93 | 0.999 |

2.7 The reaction of “Nb_n_^+^ + N_2_”


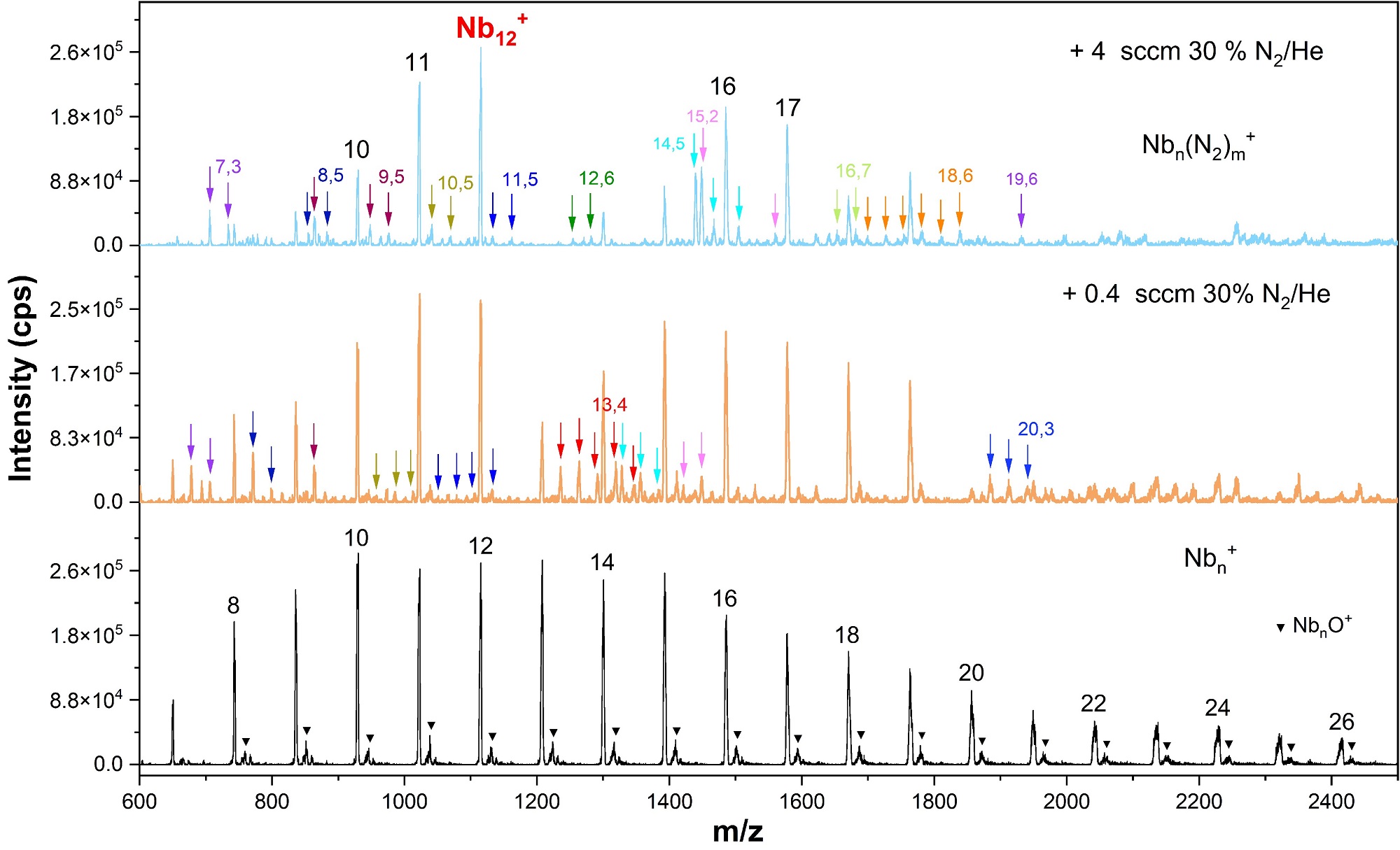


Figure S14 | Reaction of “Nb_n_^+^ + N_2_”. Mass spectra of cationic niobium clusters and after 2.2 ms reacting with 0.4, 4 sccm 30% N_2_/He. The main products of some clusters are also marked out with Nb_n_(N_2_)_m_^+^. The unit of intensity is counts per second.

2.8 The reaction of “Nb_n_^+^ + C_2_H_4_”

**
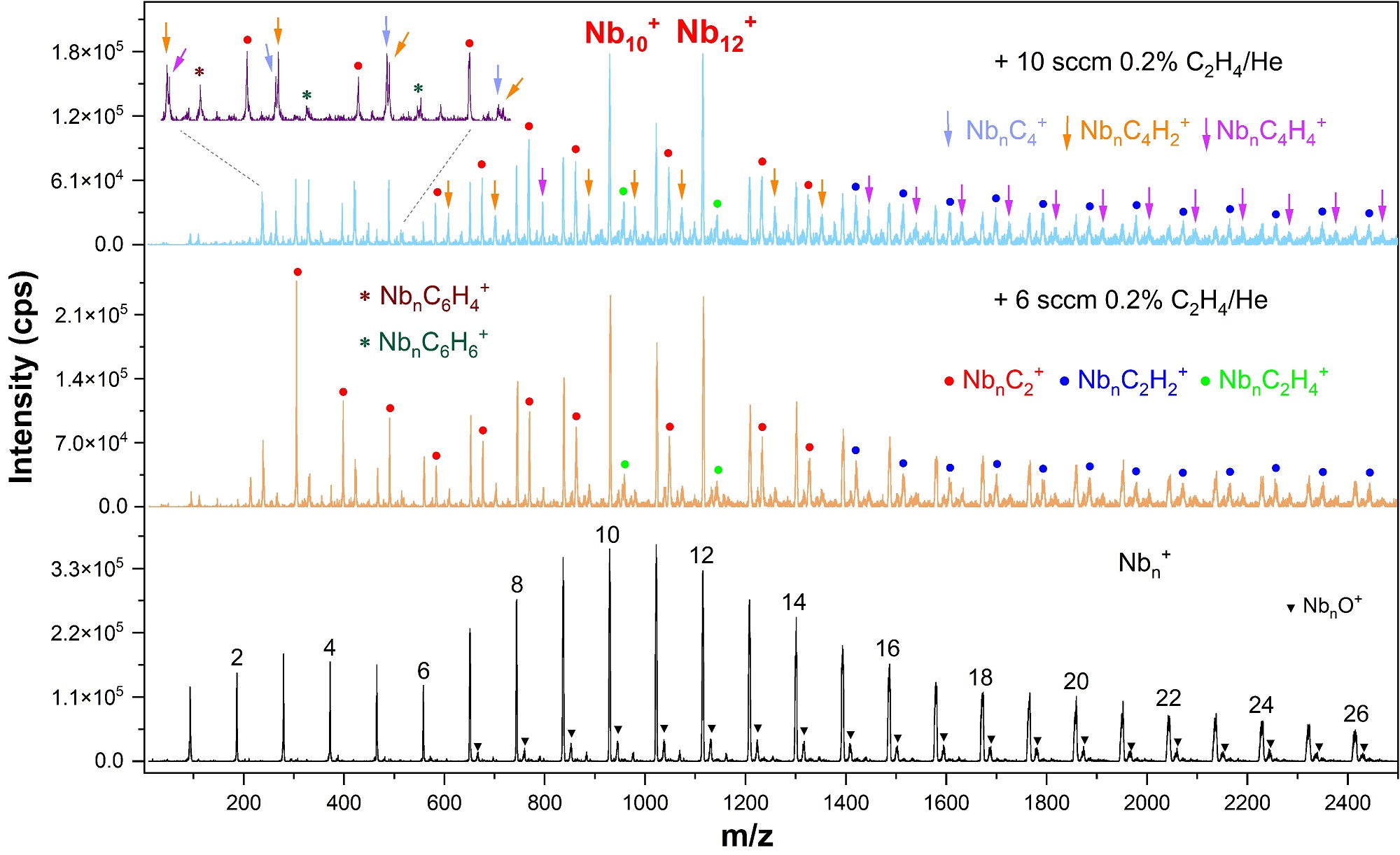
**

Figure S15 | Reaction of “Nb_n_^+^ + C_2_H_4_”. Mass spectra of cationic niobium clusters and after reacting (2.2 ms) with C_2_H_4_ (10 sccm flow, 0.2% in He). The unit of intensity is counts per second.

Considering the reactions of Nb_n_^+^ with CO, N_2_, and C_2_H_4_ all follow the pseudo-first-order rate law [33], simply we have first estimated the pseudo-first-order rate constant (*k*) by the following equation (namely mode 1, unit of cm^3^·molecule^-1^·s^-1^):

$k=- ln \frac{I}{I_{0}}/\rho t=k_{scope} /\bar{\rho}t$ (s1)

*I:* the intensity of Nb_n_^+^ after reacting with CO

*I_0_:* the intensity of Nb_n_^+^ before reacting with CO

*ρ:* the molecular number density of CO

$\bar{\rho}$: the molecular number density of a unit of CO

*t:* reaction time

On the other hand, we have also estimated the reaction rate constants based on Ferguson’s method for laminar flow tube reactors [34] (mode 2, unit of s^-1^), where the written as [35],

$K\left( T \right)=-\frac{\pi R^{2}\left\langle v_{z} \right\rangle^{2}}{Q L}\ln\left( \frac{I}{I_{0}} \right)$ (s2)

where *Q* is the flow rate of introduction of reactant gas, in units of flow [34], while the radial-dependent velocity of the gas in the flow tube is represented by:

$\boldsymbol{v}_{\boldsymbol{z}}\boldsymbol{(r)=}\frac{\left( \boldsymbol{P}_{\boldsymbol{0}}\boldsymbol{-}\boldsymbol{P}_{\boldsymbol{L}} \right)\boldsymbol{R}^{\boldsymbol{2}}}{\boldsymbol{4\mu L}}\left[ \boldsymbol{1-}\left( \frac{\boldsymbol{r}}{\boldsymbol{R}} \right)^{\boldsymbol{2}} \right]$ (s3)

Here *μ* is the viscosity of the collisional gas; *P*_0_ and *P_L_* are the pressures at the beginning and end of the flow region of length *L* (*P*_0_ > *P_L_*); r and *R* correspond to the radius of the cluster and the flow tube.

Based on mode 1 and mode 2, the estimated rate constants are listed in Table S2, where the two sets of experimental results display the same trend and the rate constant of Nb_12_^+^ locates at the lowest point compared to its neighbouring clusters.

Table S2 | Reaction rate coefficients of Nb_1-20_^+^ with CO, N_2_, C_2_H_4_ using continuity equation (Mode 1) and Ferguson’s measurements method (Mode 2).

| **Nb_n_^+^ clusters** | **Rate coefficients (10^-9^** **cm^3^·molecule^-1^·s^-1^)** | | | | | | |
| --- | --- | --- | --- | --- | --- | --- | --- |
|  | **CO** | | | **N_2_** | | **C_2_H_4_** | |
|  | **Mode 1** | **Mode 2** | **Ref[36]** | **Mode 1** | **Mode 2** | **Mode 1** | **Mode 2** |
| **Nb^+^** | - | - | - | - | - | - | - |
| **Nb_2_^+^** | - | - | - | - | - | - | - |
| **Nb_3_^+^** | 1.64 | 0.26 | - | - | - | - | - |
| **Nb_4_^+^** | 2.64 | 0.60 | 0.05 | - | - | - | - |
| **Nb_5_^+^** | 2.84 | 0.58 | 0.40 | - | - | - | - |
| **Nb_6_^+^** | 3.55 | 0.93 | 1.3 | - | - | - | - |
| **Nb_7_^+^** | 3.84 | 1.01 | 1.3 | 0.49 | 0.139 | 16.91 | 4.83 |
| **Nb_8_^+^** | 3.98 | 1.06 | 1.1 | 0.11 | 0.031 | 15.35 | 4.39 |
| **Nb_9_^+^** | 4.24 | 1.24 | 1.8 | 0.05 | 0.015 | 15.72 | 4.49 |
| **Nb_10_^+^** | 4.37 | 1.30 | 1.2 | 0.01 | 0.002 | 7.01 | 2.00 |
| **Nb_11_^+^** | 4.29 | 1.34 | 1.9 | 0.004 | 0.001 | 13.56 | 3.88 |
| **Nb_12_^+^** | 2.09 | 0.69 | 0.35 | 0.005 | 0.001 | 4.58 | 1.31 |
| **Nb_13_^+^** | 4.11 | 1.19 | 1.6 | 0.70 | 0.2 | 14.80 | 4.23 |
| **Nb_14_^+^** | 4.69 | 1.34 | 1.5 | 0.45 | 0.128 | 16.19 | 4.63 |
| **Nb_15_^+^** | 4.78 | 1.42 | 1.2 | 0.16 | 0.045 | 16.23 | 4.64 |
| **Nb_16_^+^** | 4.86 | 1.44 | 1.5 | 0.03 | 0.01 | 15.74 | 4.50 |
| **Nb_17_^+^** | 4.81 | 1.46 | 1.3 | 0.006 | 0.002 | 14.72 | 4.21 |
| **Nb_18_^+^** | 5.06 | 1.49 | 1.3 | 0.07 | 0.021 | 16.08 | 4.59 |
| **Nb_19_^+^** | 5.09 | 1.49 | 1.5 | 0.008 | 0.002 | 16.54 | 4.73 |
| **Nb_20_^+^** | 5.59 | 1.56 | 1.5 | 1.10 | 0.315 | 17.12 | 4.89 |

**Note:** The reaction rate constants of the reference were reported by Iulia Balteanu et al.[36] using the FT-ICR mass spectrometer.

3. Theoretical Calculations on Nb_n_^+^

3.1 Global search and structure determination


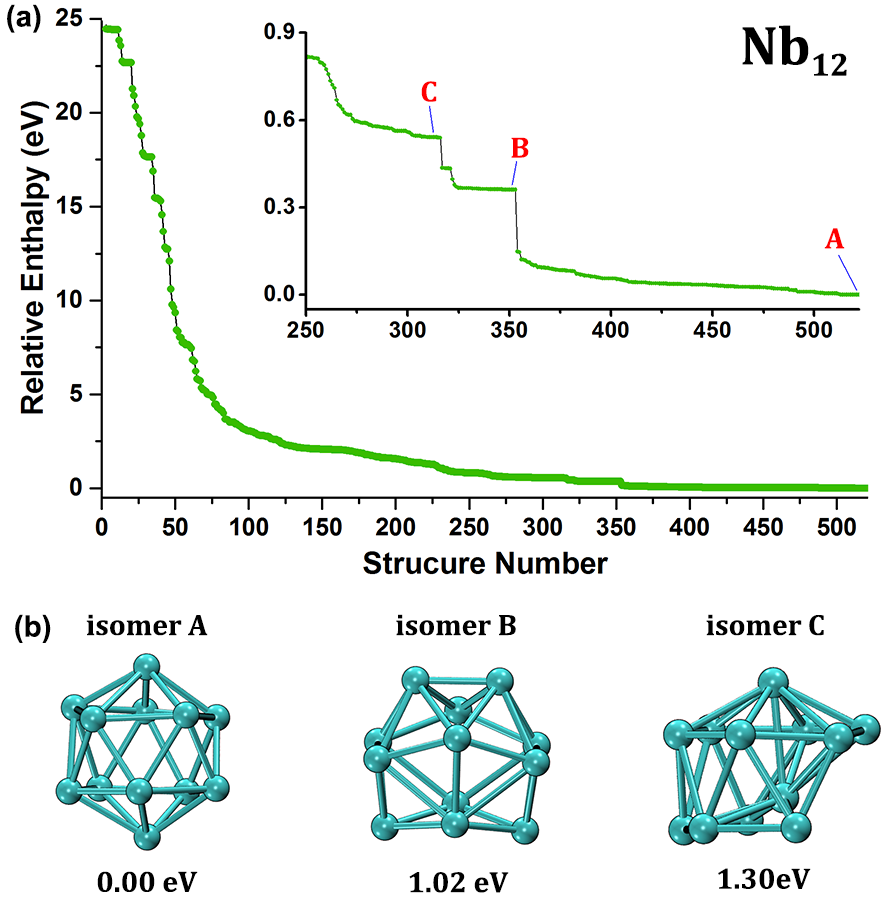


Figure S16 | Global research of Nb_12_^+^. (a) The global research of Nb_12_ based on USPEX combined with VASP software. The energies are relative to the global minimum structure. The structures with relative lower energies are labelled by structures A, B, and C. (b) Optimized isomers, and the relative zero-point energies (eV) of cationic Nb_12_^+^ with structures A-C as initial guess calculated at BPW91/Lanl2TZ(f) level using G09 grogram.


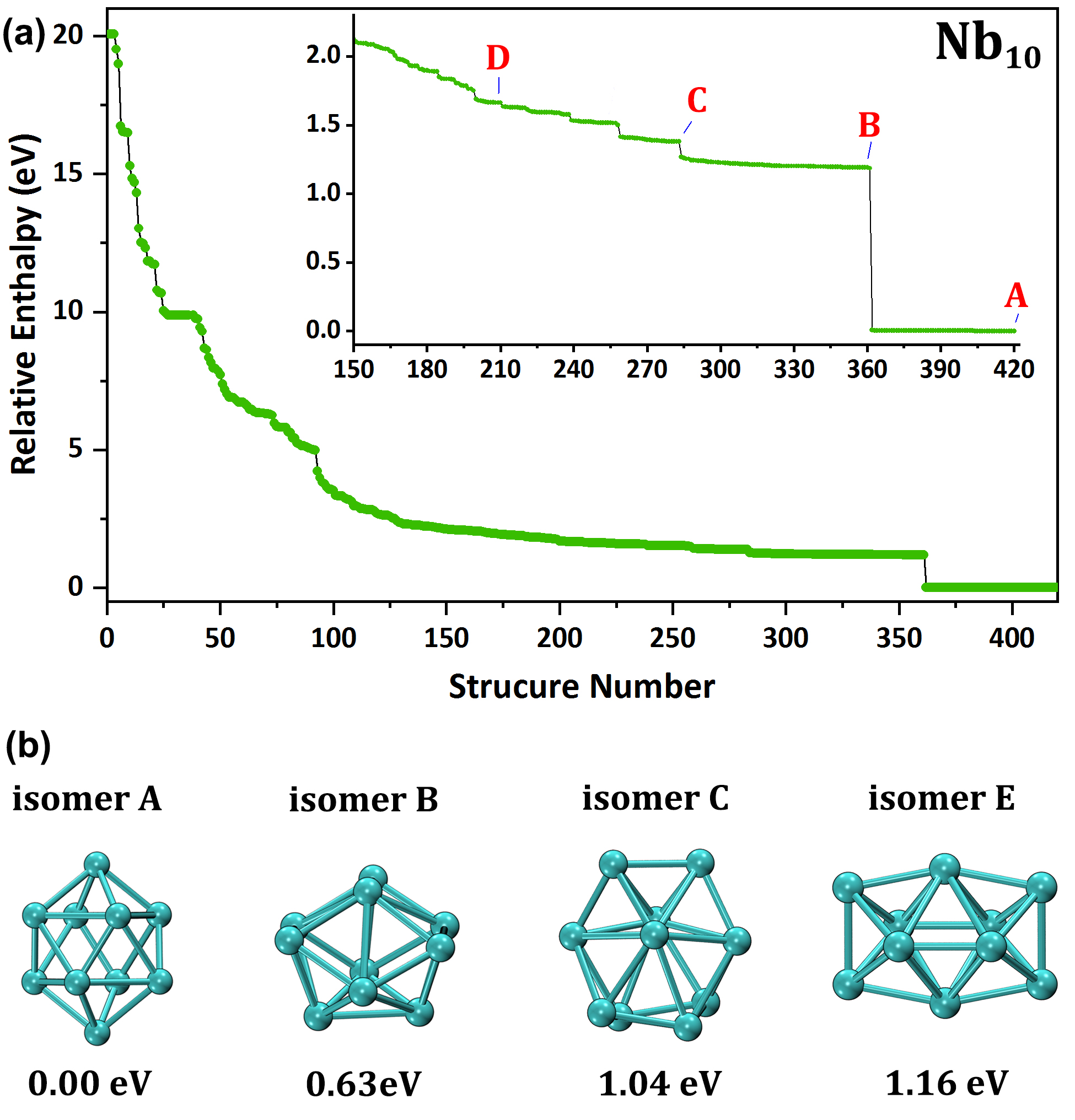


Figure S17 | Global research of Nb_10_^+^. (a) The global research of Nb_10_ based on USPEX combined with VASP software. The energies are relative to the global minimum structure. The structures with relative lower energies are labelled by structures A, B, C, and D. (b) Optimized isomers, and the relative zero-point energies (eV) of cationic Nb_13_^+^ with structures A-D as initial guess calculated at BPW91/Lanl2TZ(f) level using G09 grogram.


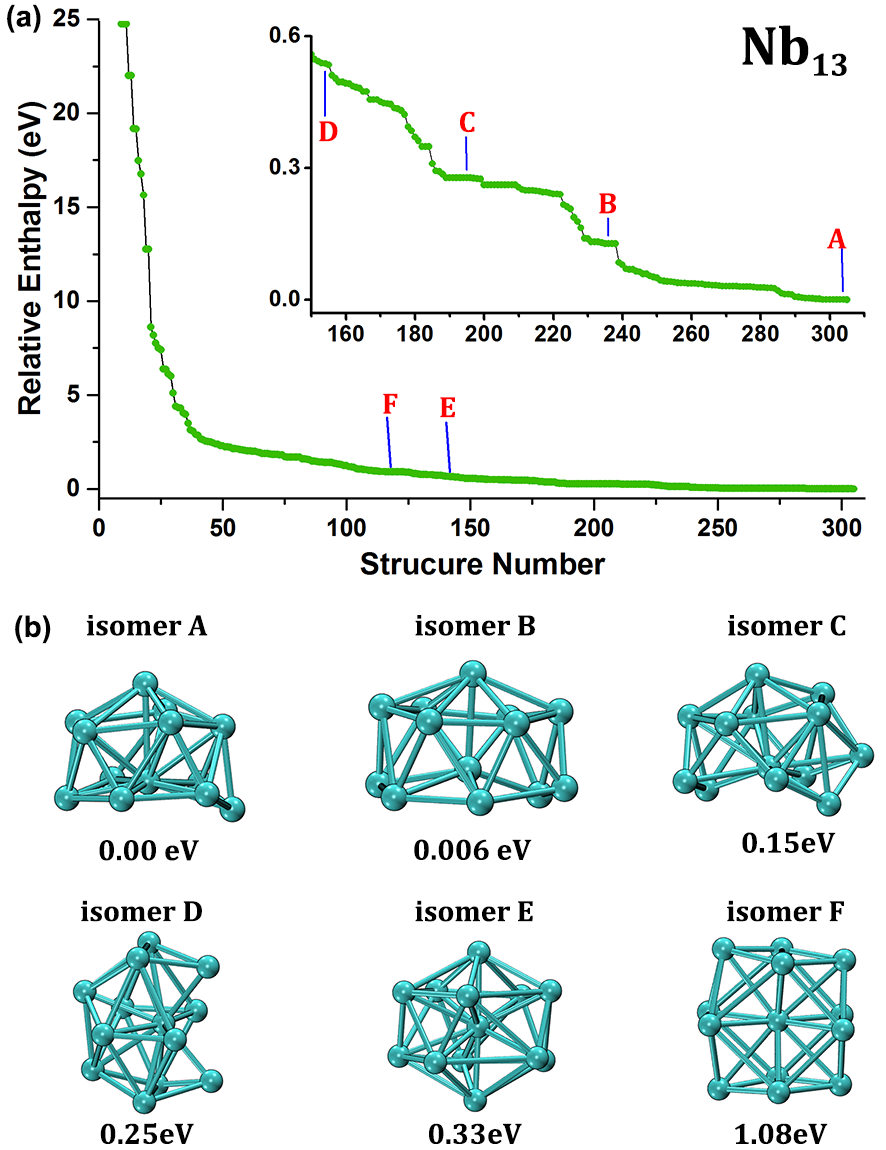


Figure S18 | Global research of Nb_13_^+^. (a) The global research of Nb_13_ based on USPEX combined with VASP software. The energies are relative to the global minimum structure. The structures with relative lower energies are labelled by structures A, B, C, D, and E. (b) Optimized isomers and the relative zero-point energies (eV) of cationic Nb_13_^+^ with structures A-E as initial guess calculated at BPW91/Lanl2TZ(f) level using G09 grogram.

3.2 Charge population and electrostatic potential


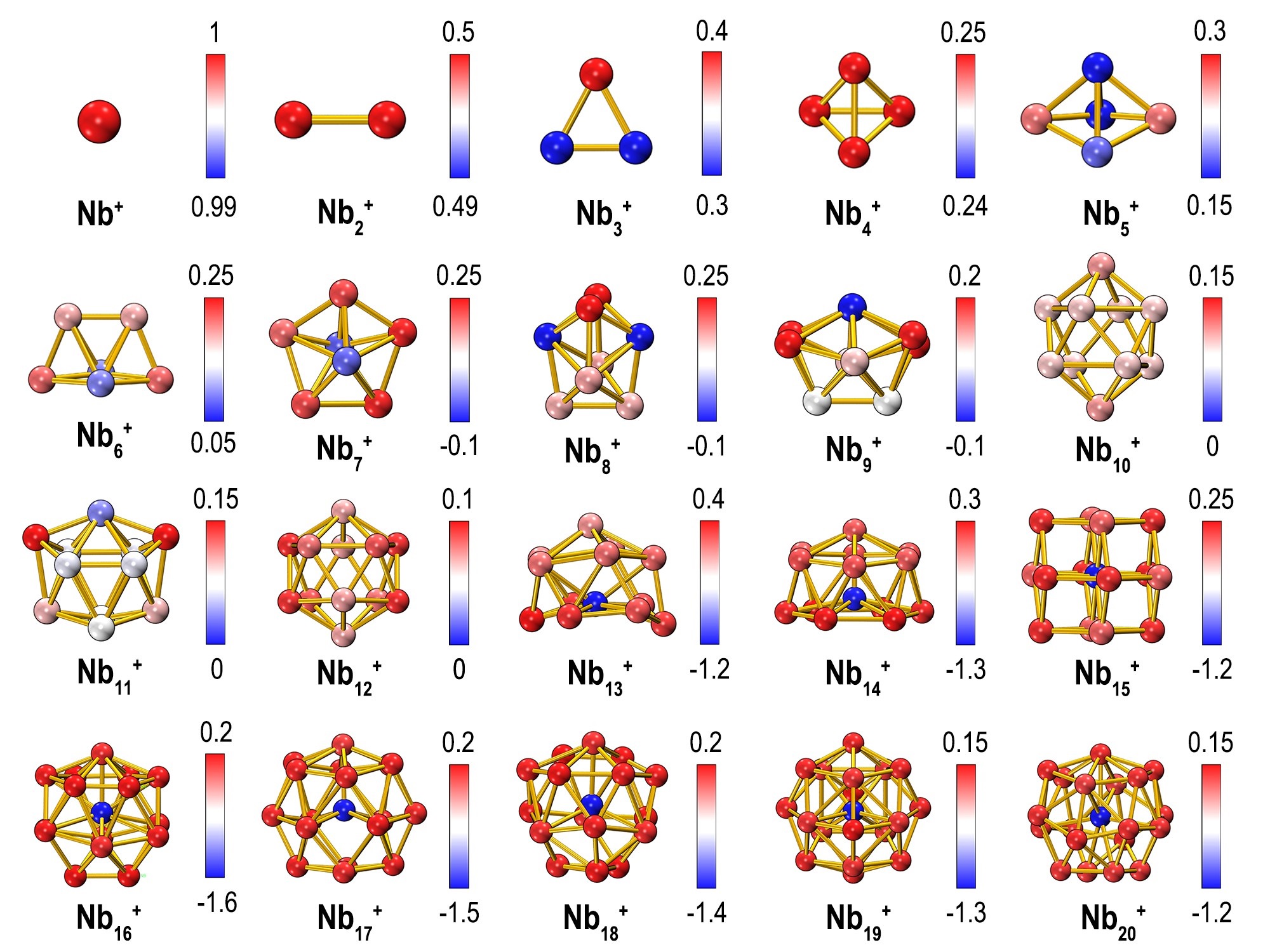


Figure S19 | NPA charge population of Nb_n_^+^ (n=1-20) clusters. The color scale from blue to red corresponds to charge variation.


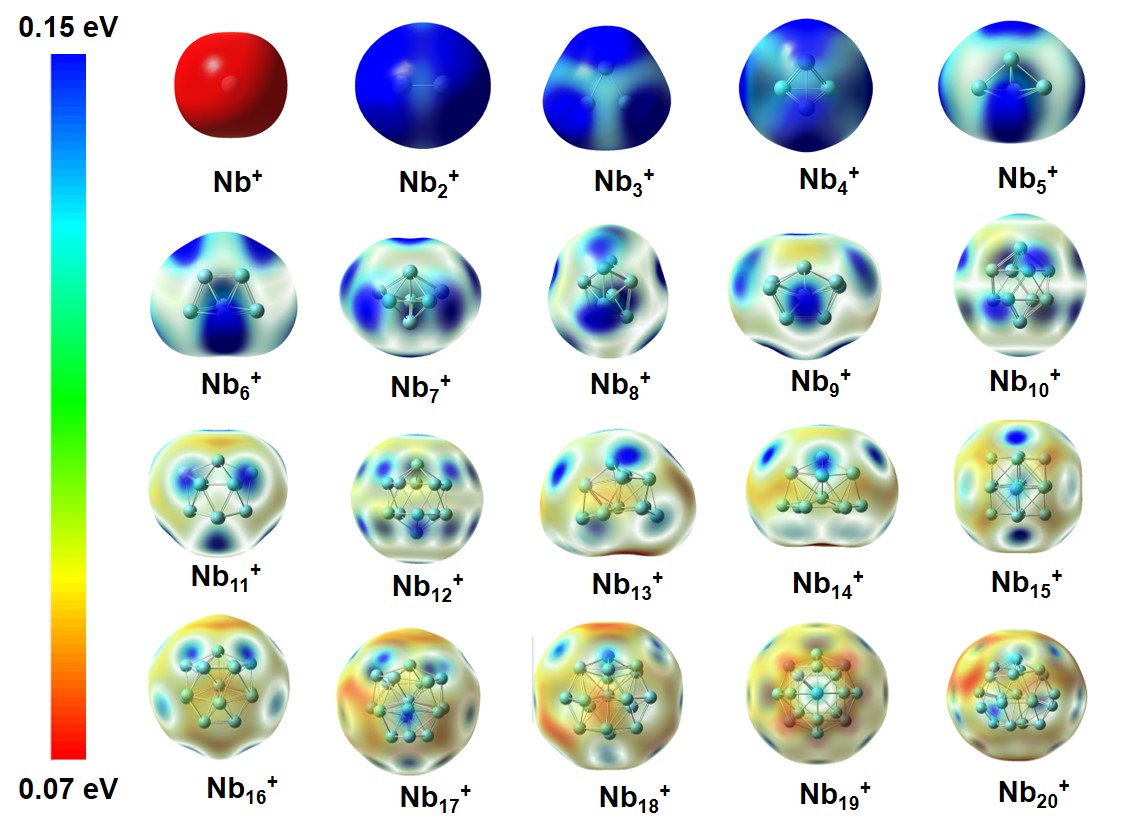


Figure S20 | Electrostatic surface potential of Nb_n_^+^ (n=1-20) clusters. The blue area corresponds to a large ESP value, while the red color region refers to a small ESP value.

3.3 Molecular dynamics simulation

We have conducted Born–Oppenheimer molecular dynamics (BOMD) simulations for the Nb_12_^+^, Nb_10_^+^, and Nb_12_^2+^ clusters respectively for 5 ps using the software suite of CP2K [27]. The results are shown below. It is shown that the superatom cluster Nb_12_^+^ has outstanding thermal stability with the cage structures undissociated up to 1200K.


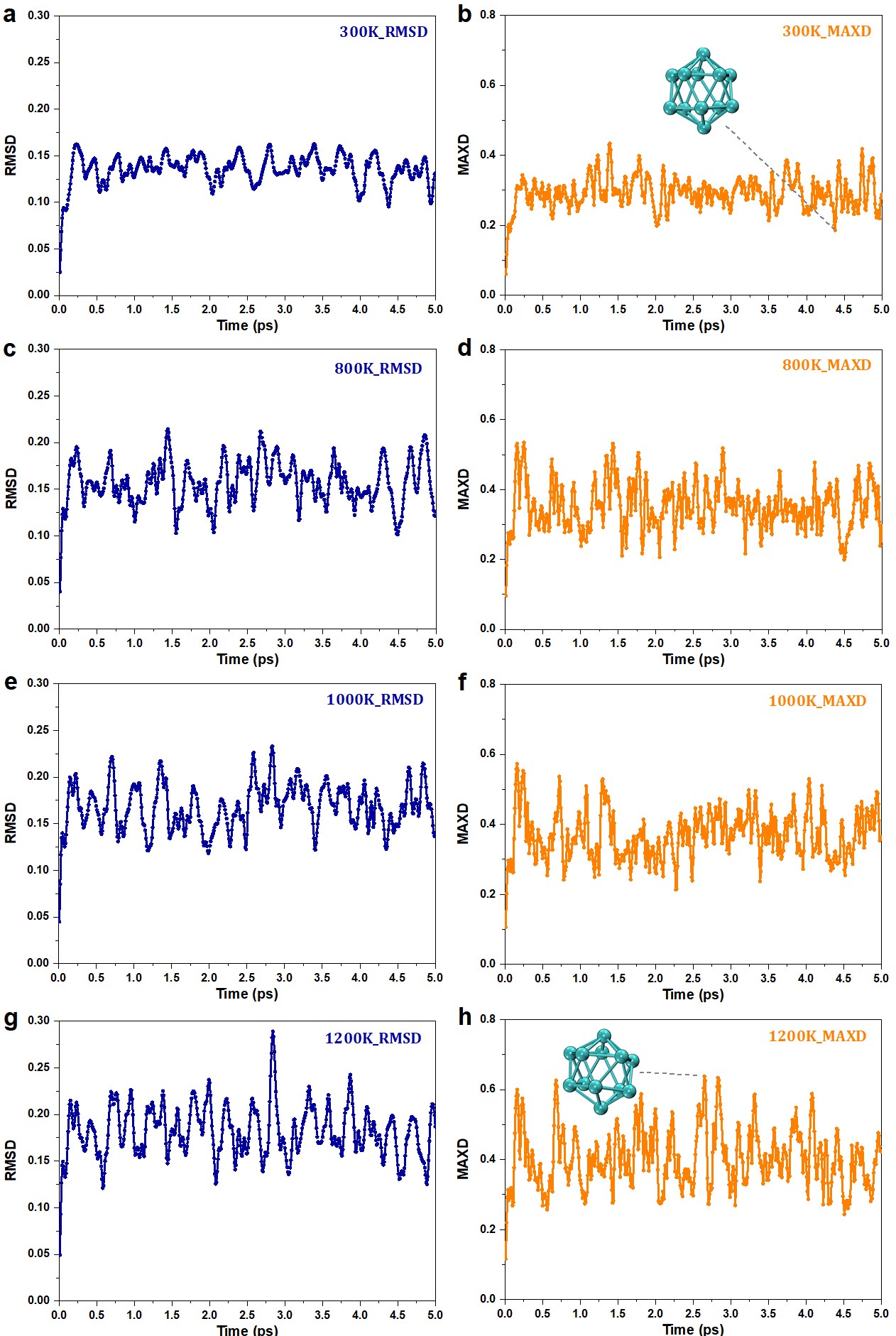


Figure S21 | Molecular dynamics simulation of Nb_12_^+^. BOMD of Nb_12_^+^ at 300K, 800K, 1000K and 1200K for 5 ps with the average root-mean-square-deviation (RMSD) values and maximum bond length deviation (MAXD) values indicated in Å. The insets show the structures with the smallest and largest deformation


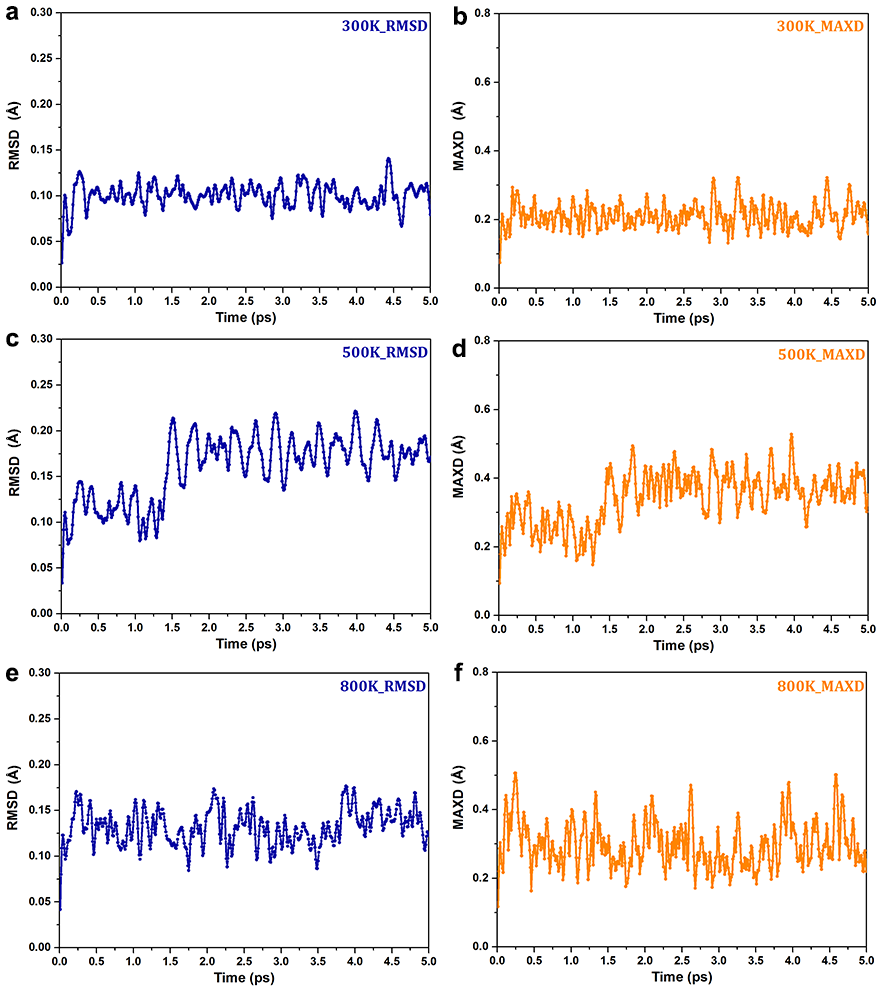


Figure S22 | Molecular dynamics simulation of Nb_10_^+^. Born-Oppenheimer molecular dynamics (BOMD) simulations of Nb_10_^+^ at 300K, 500K, and 800K for 5 ps with the average root-mean-square-deviation (RMSD) values and maximum bond length deviation (MAXD) values indicated in Å.


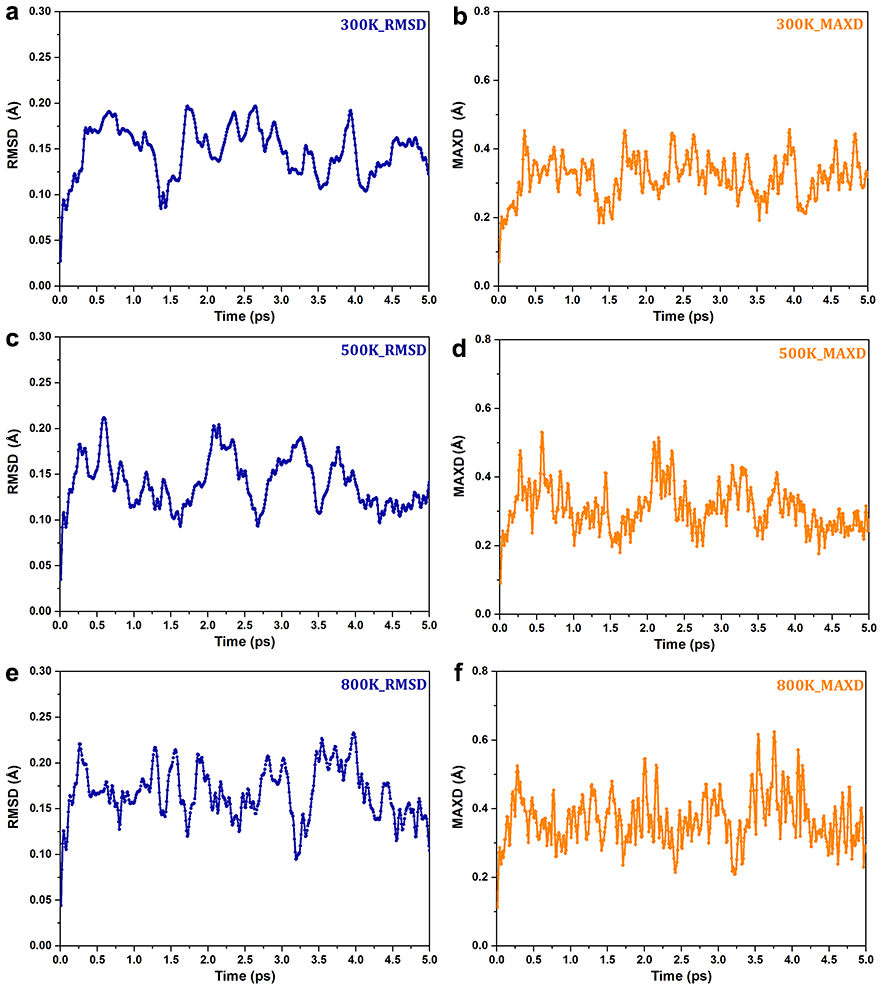


Figure S23 | Molecular dynamics simulation of Nb_12_^2+^. BOMD of Nb_12_^2+^ at 300K, 500K, and 800K for 5 ps with the average root-mean-square-deviation (RMSD) values and maximum bond length deviation (MAXD) values indicated in Å.

3.4 PDOS of Nb_n_^+^ clusters


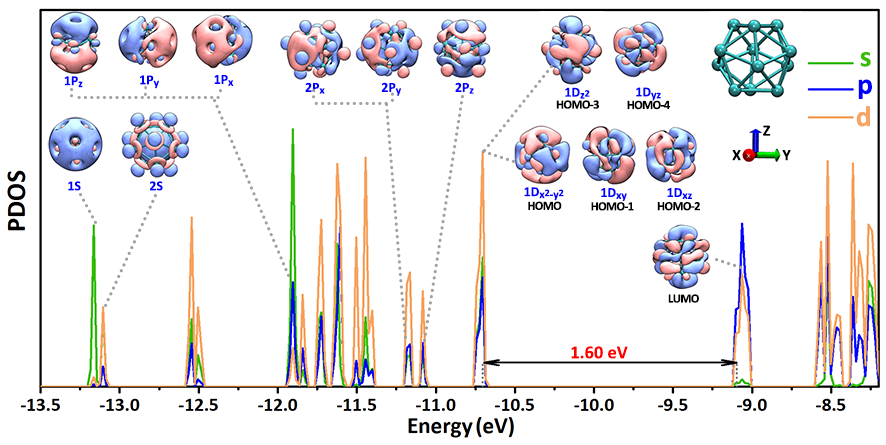


Figure S24 | DOS of Nb_12_^2+^. Partial density of states (PDOS) and selected canonical molecular orbitals (CMOs) of Nb_12_^2+^, calculated at BPW91/Lanl2TZ(f) level. The HOMO-LUMO gap is given in eV. The green, blue, and orange curves indicate the PDOS of s, p, and d shell types respectively.


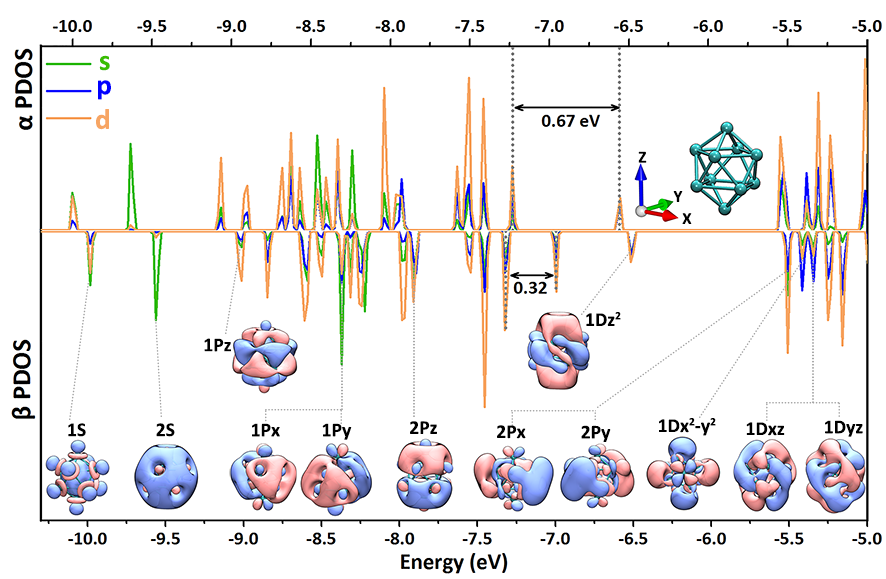


Figure S25 | DOS of Nb_10_^+^. Partial density of states (PDOS) and selected canonical molecular orbitals (CMOs) of Nb_10_^+^, calculated at BPW91/Lanl2TZ(f) level. The HOMO-LUMO gap is given in eV. The green, blue, and orange curves indicate the PDOS of s, p, and d shell types respectively.

3.5 Molecule orbitals and NAO analysis


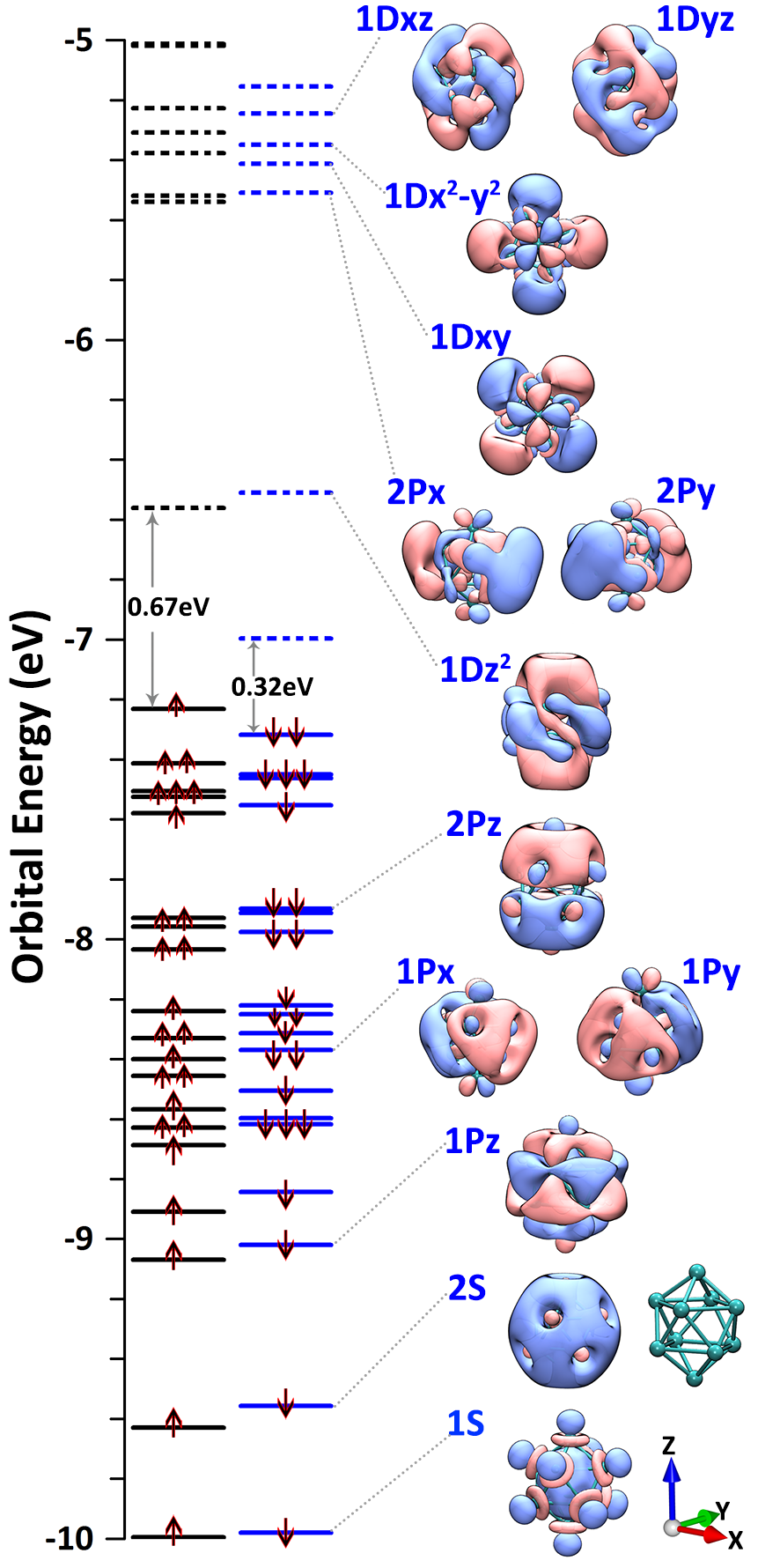


Figure S26 | MOs of Nb_10_^+^ Canonical molecular orbitals and the superatomic features for the valence electrons of Nb_10_^+^ calculated at BPW91/Lanl2TZ(f) level.

3.6 HOMO and LUMO energy levels


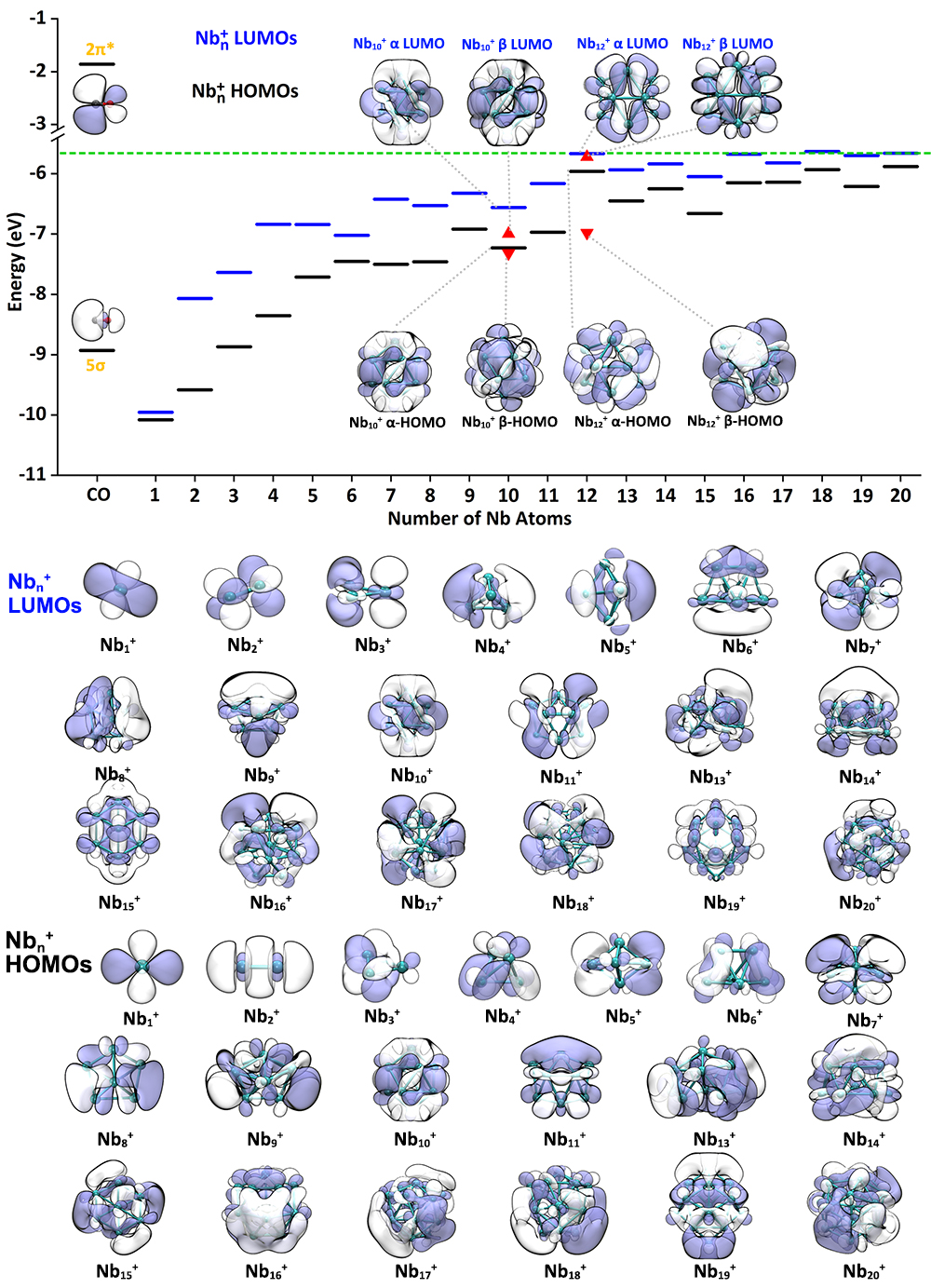


Figure S27 | MO Energy Levels. The alpha-HOMOs (black line) and alpha-LUMOs (blue line) of the Nb_n_^+^ (n=1-20) clusters, with a comparison to that of CO calculated at BPW91/Lanl2TZ(f) level of theory. For Nb_10_^+^ and Nb_12_^+^, the β-HOMO and β-LUMO energy levels are also displayed (red triangle).

3.7 MOs of Nb_n_^+^ at B3LYP/Lanl2TZ(f) Level


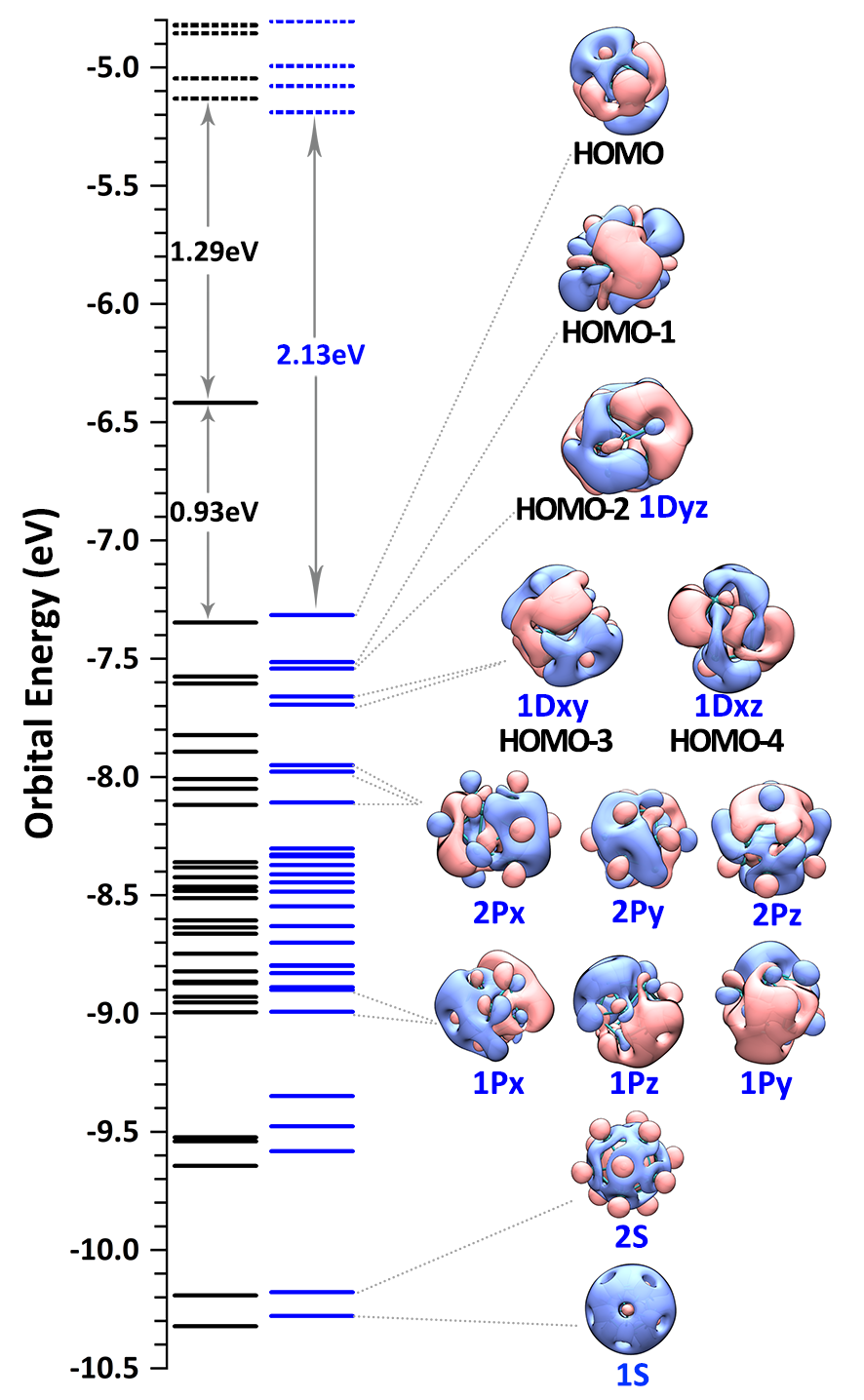


Figure S28 | Canonical molecular orbitals of Nb_12_^+^ with superatomic features for the valence electrons, calculated at B3LYP/Lanl2TZ(f) Level of theory.

**
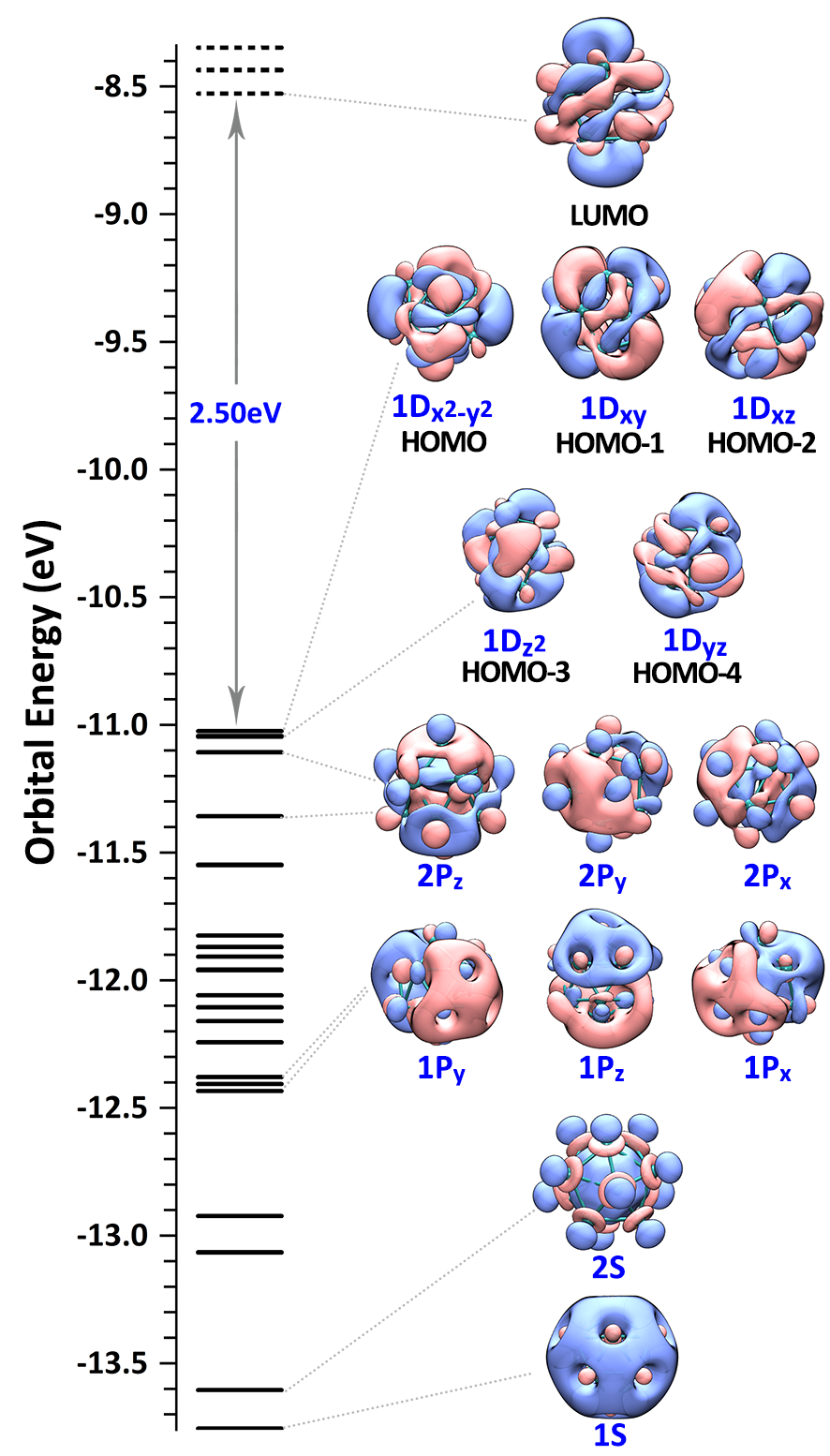
**

Figure S29 | Canonical molecular orbitals of Nb_12_^2+^. The Canonical molecular orbitals of Nb_12_^2+^ with superatomic features for the valence electrons, calculated at B3LYP/Lanl2TZ(f) Level of theory.


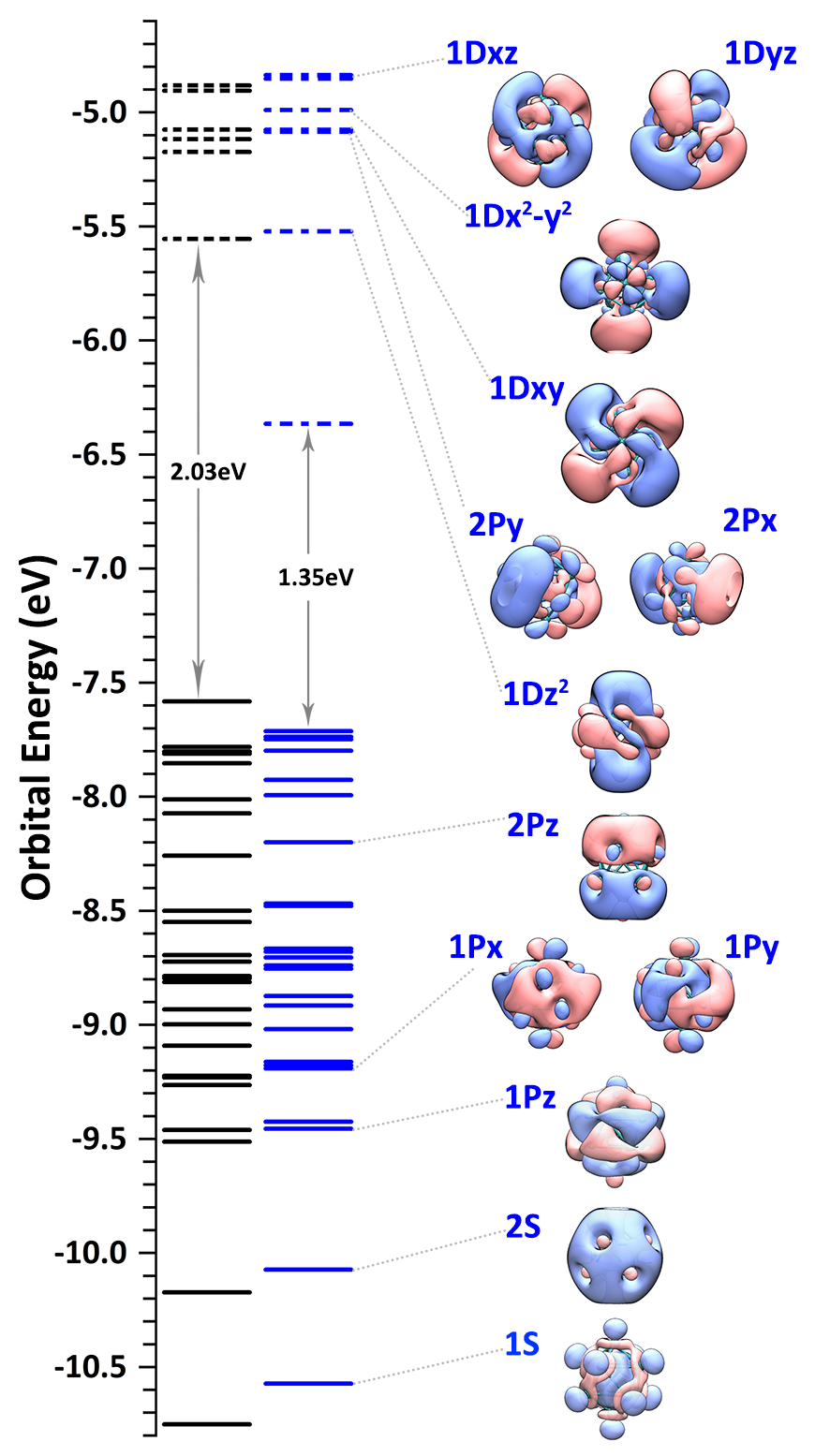


Figure S30 | Canonical molecular orbitals of Nb_10_^+^. The Canonical molecular orbitals of Nb_10_^+^ with superatomic features for the valence electrons, calculated at B3LYP/Lanl2TZ(f) Level of theory.

Seen from the canonical molecular orbitals of Nb_10_^+^ (Fig. S30), it is interesting to note that the energy-descent superatomic 1S orbital composed of d-states locates in the lower energy levels than the 2S orbital composed of s electrons, indicative of a more compact d-orbital overlap interactions in Nb_10_^+^. This is in sharp contrast to the Nb_12_^+^ and Nb_12_^2+^ (Figs S28 and S29) of which more superatomic orbitals are filled with delocalized electrons and 5s-contributed superatomic 1S orbital locates in the lowest energy levels enabling for a better balance of nuclei-electron interactions thus enhanced stability of the cluster.

It is worth mentioning that, the HOMO-LUMO gaps of these canonical molecular orbitals are even larger when applying the B3LYP/Lanl2TZ(f) Level of theory, as shown below. While the overall trend is consistent with each other, and considering the DFT-method test as shown in Figure S2, we emphasize the results by BPW91 level of theory for all the calculations of the niobium clusters.


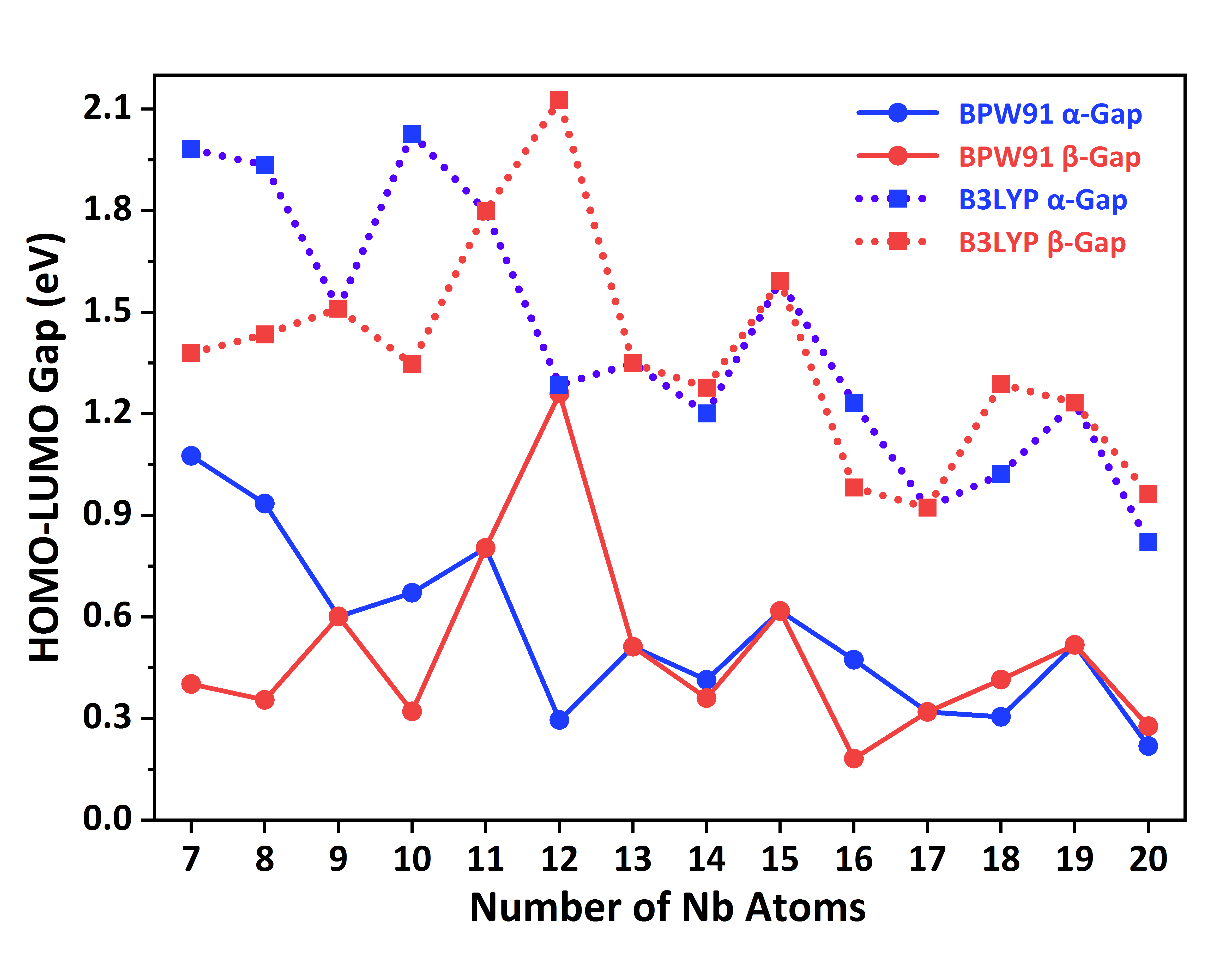


Figure S31 | The HOMO-LUMO gaps of Nb_n_^+^ (n=7-20) clusters calculated at B3LYP/Lanl2TZ(f) Level (dot lines), in a comparison with that calculated at BPW91/Lanl2TZ(f) level of theory (solid lines). The blue and red dash/solid lines correspond to alpha and beta orbitals.

3.8 A comparison of Nb_n_^+^ with V_n_^+^

In addition, we have made a comparison of the structures of Nb_n_^+^ and V_n_^+^, as well as the DOS of Nb_12_^+^ vs V_12_^+^, as shown below. It is shown that for the M_n_^+^ (M=Nb, V) the central atom emerges at n=11 for vanadium while n=13 for niobium, which accounts for their different size-dependent stability.

**
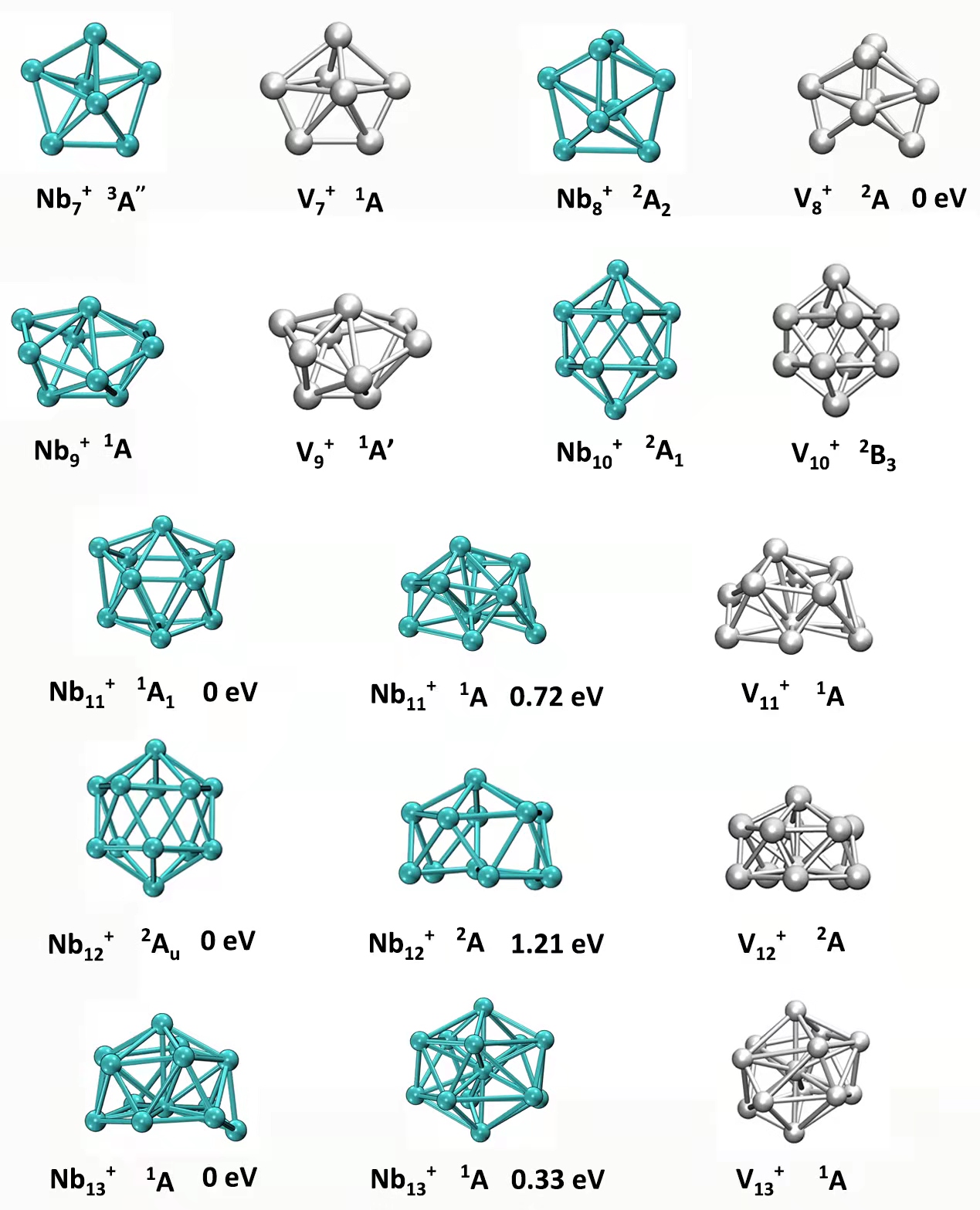
**

Figure S32 | Structures Comparison of Nb_n_^+^ and V_n_^+^ (n=7-13). The structures and electronic states of ground state Nb_n_^+^ and V_n_^+^ clusters. The structures of V_n_^+^ clusters are referred to the literature [37].

3.9 A comparison of Nb_10_^+^ with Nb_12_^+^


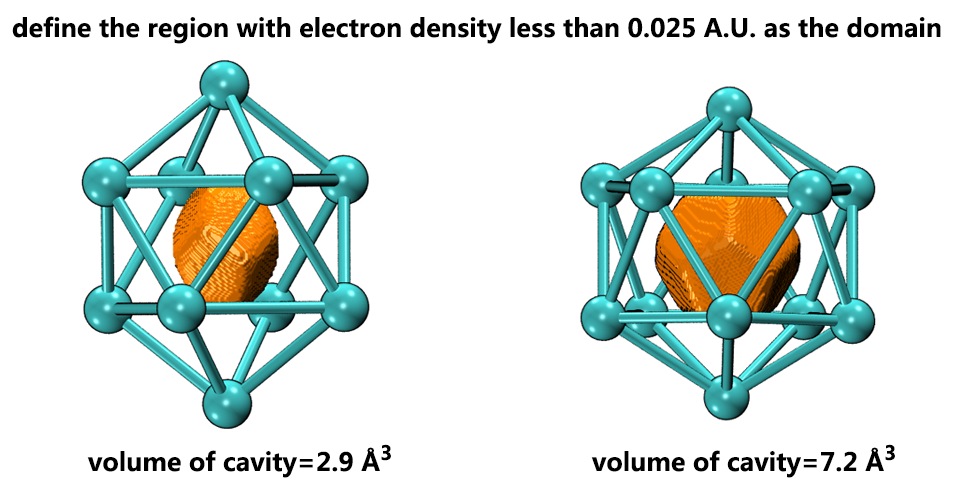


Figure S33 | The cavity volume. The inner cavity of Nb_10_^+^ and Nb_12_^+^. The volume of the cavity is the region in which the electron density is less than 0.025 A.U.

Comparing with the IR-active vibrations of the two clusters, it is seen that the Nb_10_^+^ displays more active modes of varying frequencies, especially the #3 and #1 modes which correspond to the deformation of the cage structure.


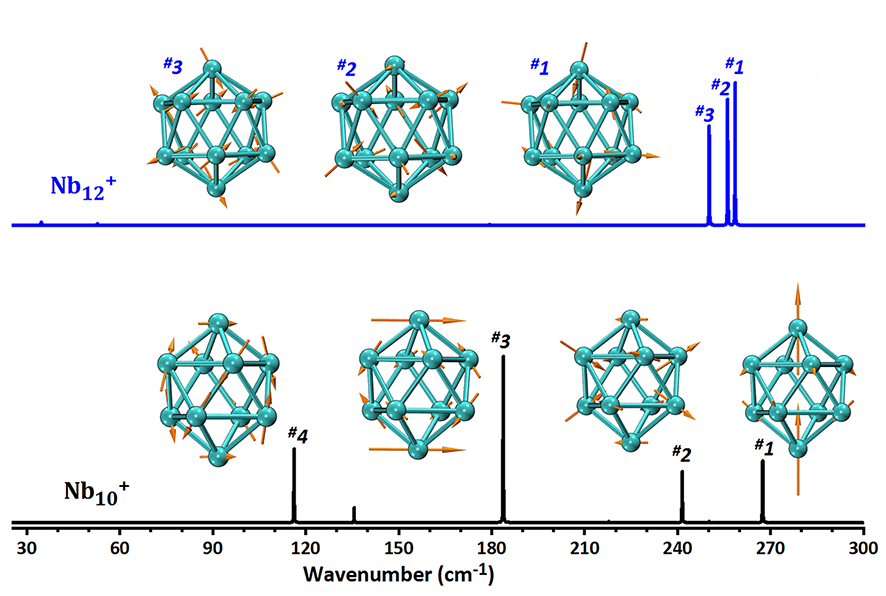


Figure S34 | Vibration Frequency of Nb_10/12_^+^. IR-active vibrational spectra and the main vibration modes of Nb_10/12_^+^ calculated at BPW91/Lanl2TZ(f) level.

4. CO Adsorption

4.1 Geometric and electronic structures


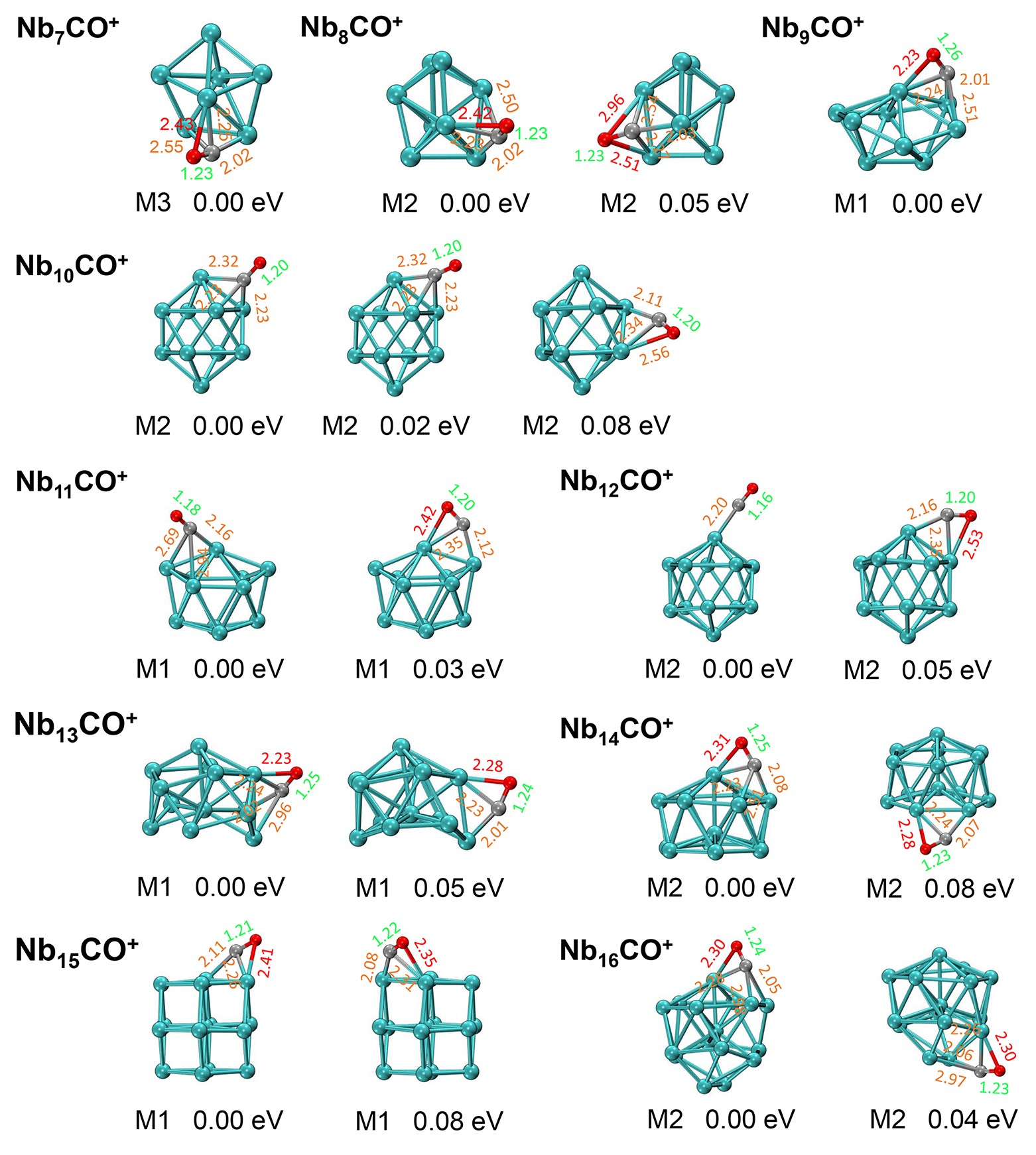


**Figure S35 | Structure of Nb_n_CO^+^.** Optimized structures of Nb_n_CO^+^ (n=7-16) isomers. All the data are calculated at the BPW91/Lanl2TZ(f) level for Nb_n_^+^ and BPW91/6-311G(d) level for CO using the G09 program. Spin multiplicity and relative energy difference (in eV) are given below the structures. Atoms Nb, C, and O are indicated by cyan, grey, and red colour respectively. The length of C-O is marked in green, while Nb-C is marked in orange, Nb-O is marked in red, and the unit is Å.

**Table S3** | **Wiberg Bond order and bond length** of C-O, C-Nb, O-Nb, and C/O-Nb total order in Nb_7-16_CO^+^.

| **Species** | **C-O** | | **C-Nb (1)** | | **C-Nb (2)** | | **C-Nb (3)** | | **O-Nb (1)** | | **C/O-Nb**  **total order** |
| --- | --- | --- | --- | --- | --- | --- | --- | --- | --- | --- | --- |
|  | **Length** | **Order** | **Length** | **Order** | **Length** | **Order** | **Length** | **Order** | **Length** | **Order** |  |
| **Nb_7_CO^+^** | 1.23 | 1.61 | 2.02 | 1.13 | 2.25 | 0.48 | 2.55 | 0.33 | 2.43 | 0.30 | 2.24 |
| **Nb_8_CO^+^** | 1.23 | 1.61 | 2.02 | 1.05 | 2.23 | 0.54 | 2.5 | 0.34 | 2.42 | 0.30 | 2.23 |
| **Nb_9_CO^+^** | 1.26 | 1.46 | 2.01 | 1.17 | 2.24 | 0.45 | 2.51 | 0.38 | 2.23 | 0.45 | 2.45 |
| **Nb_10_CO^+^** | 1.20 | 1.82 | **2.23** | 0.59 | 2.23 | 0.60 | 2.32 | 0.46 | - | - | 1.65 |
| **Nb_11_CO^+^** | 1.18 | 2.02 | 2.16 | 0.86 | 2.69 | 0.24 | 2.94 | 0.19 | - | - | 1.29 |
| **Nb_12_CO^+^** | **1.16** | 2.17 | **2.20** | 0.82 | - | - | - | - | - | - | 0.82 |
| **Nb_13_CO^+^** | 1.25 | 1.50 | 2.02 | 1.16 | 2.24 | 0.51 | 2.96 | 0.22 | 2.23 | 0.45 | 2.34 |
| **Nb_14_CO^+^** | 1.23 | 1.63 | 2.08 | 0.94 | 2.23 | 0.53 | 2.21 | 0.33 | 2.31 | 0.40 | 2.21 |
| **Nb_15_CO^+^** | 1.21 | 1.75 | 2.11 | 0.93 | 2.26 | 0.53 | - | - | 2.41 | 0.31 | 1.77 |
| **Nb_16_CO^+^** | 1.24 | 1.58 | 2.05 | 1.06 | 2.28 | 0.47 | 2.98 | 0.02 | 2.29 | 0.39 | 1.94 |


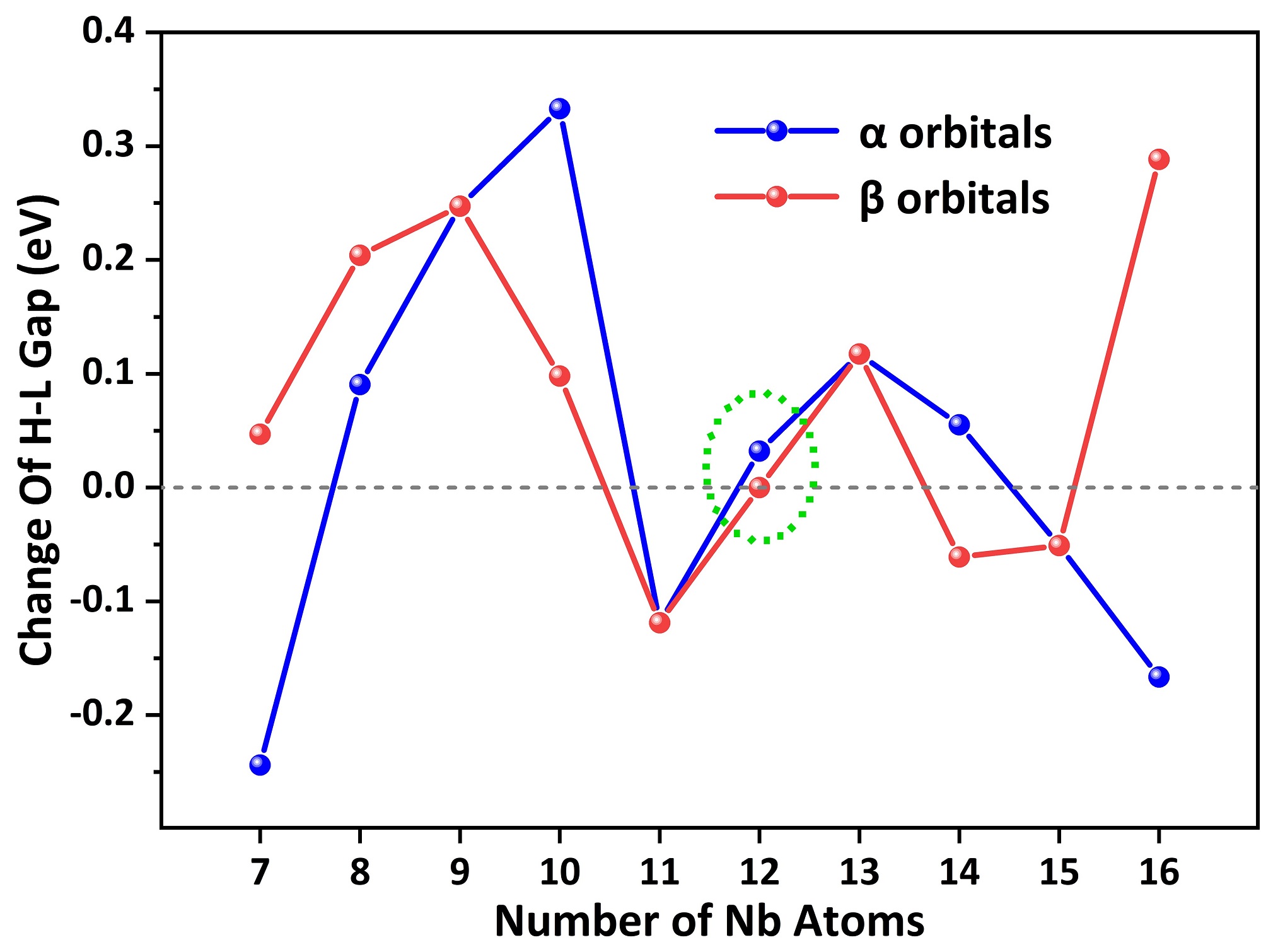


Figure S36 | The HOMO-LUMO gaps of **Nb_n_CO^+^ vs. Nb_n_^+^**. The Change of α and β HOMO-LUMO gaps between Nb_n_CO^+^ and Nb_n_^+^ (n=7-16), calculated at BPW91 Lanl2TZ(f) Level of theory.


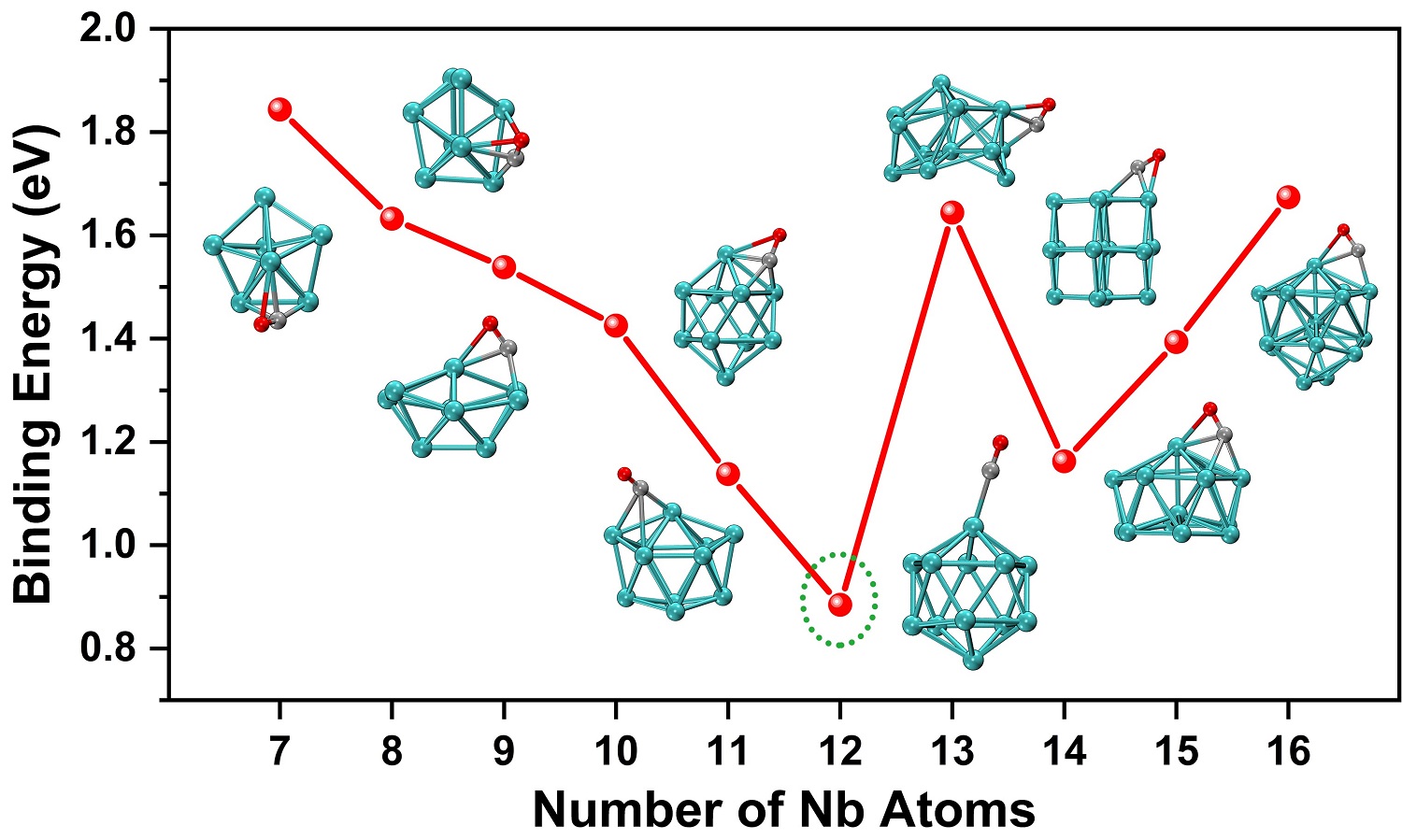


Figure S37 | Binding Energy of Nb_n_CO^2+^. The calculated binding energy of Nb_n_^2+^ (n=7-16) with CO based on optimized lowest-energy structures of Nb_7-16_CO^2+^. The insets show the structures of Nb_7-16_CO^2+^.


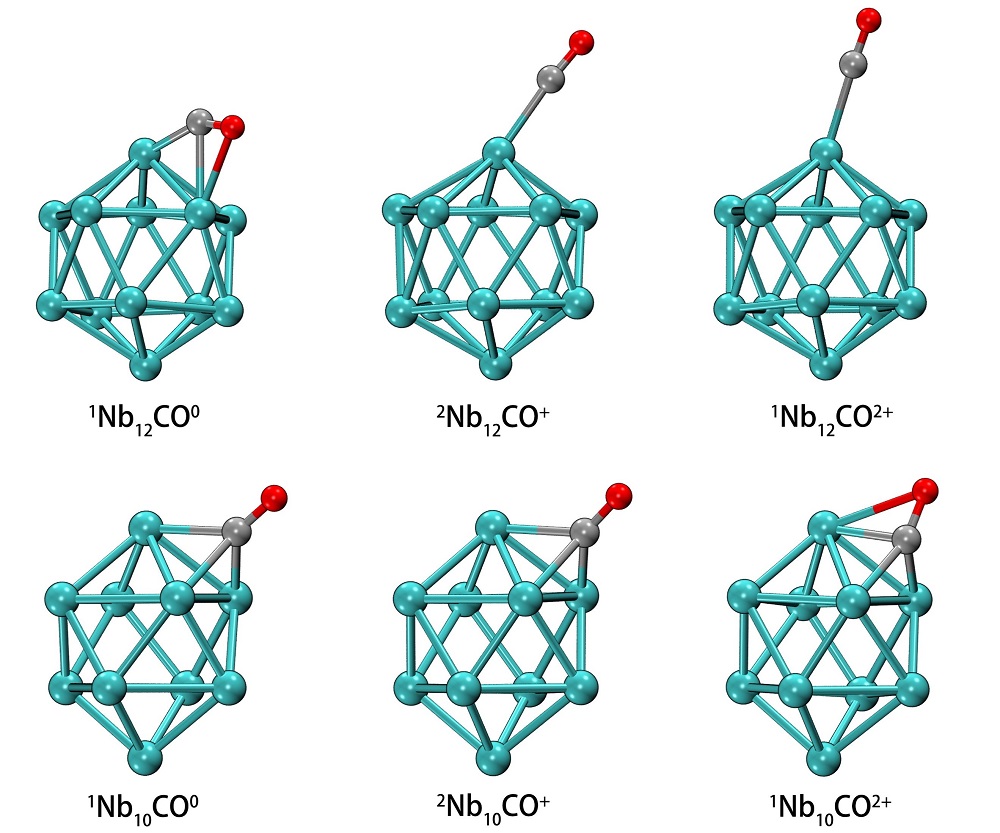


Figure S38 | **Structure of Nb_12,10_(CO)^0/+/2+^.** Optimized lowest-energy structures of ^1^Nb_12_,_10_CO^0^, ^2^Nb_12,10_CO^+^, and ^1^Nb_12,10_CO^2+^. All the data are calculated at the BPW91/Lanl2TZ(f) level for Nb_n_^+^ and BPW91/6-311G(d) level for CO using the G09 program.

**Table S4** | **Binding energy and NPA charge** on CO of optimized ^1^Nb_12,10_CO^0^, ^2^Nb_12,10_CO^+^, and ^1^Nb_12,10_CO^2+^ at the BPW91/Lanl2TZ(f) level.

|  | **ΔE_b_ (eV)** | **NPA Charge On CO** |  |  | **ΔE_b_ (eV)** | **NPA Charge On CO** |
| --- | --- | --- | --- | --- | --- | --- |
| **^1^Nb_12_CO^0^** | 1.38 | -0.19 | **^1^Nb_10_CO^0^** |  | 1.14 | -0.27 |
| **^2^Nb_12_CO^+^** | 0.98 | 0.16 | **^2^Nb_10_CO^+^** |  | 1.22 | -0.17 |
| **^1^Nb_12_CO^2+^** | 0.88 | 0.19 | **^1^Nb_10_CO^2+^** |  | 1.43 | -0.17 |

Extensive studies have been reported upon the coordination of CO with the transition metal atoms/ions as well as CO adsorption on the clusters [36, 38-47]. It has been illustrated that the catalysis is highly sensitive to the local structure and charge states, size and components, polarization, coordination, and electron transfer interactions [48-51]. For this purpose, we have simply studied the multiple CO molecules on the Nb_12_^+^ and Nb_10_^+^ clusters. As result, end-on adsorption is favoured for Nb_12_(CO)_2,4_^+^ while the hollow site adsorption retains for Nb_10_(CO)_2,4_^+^.

**
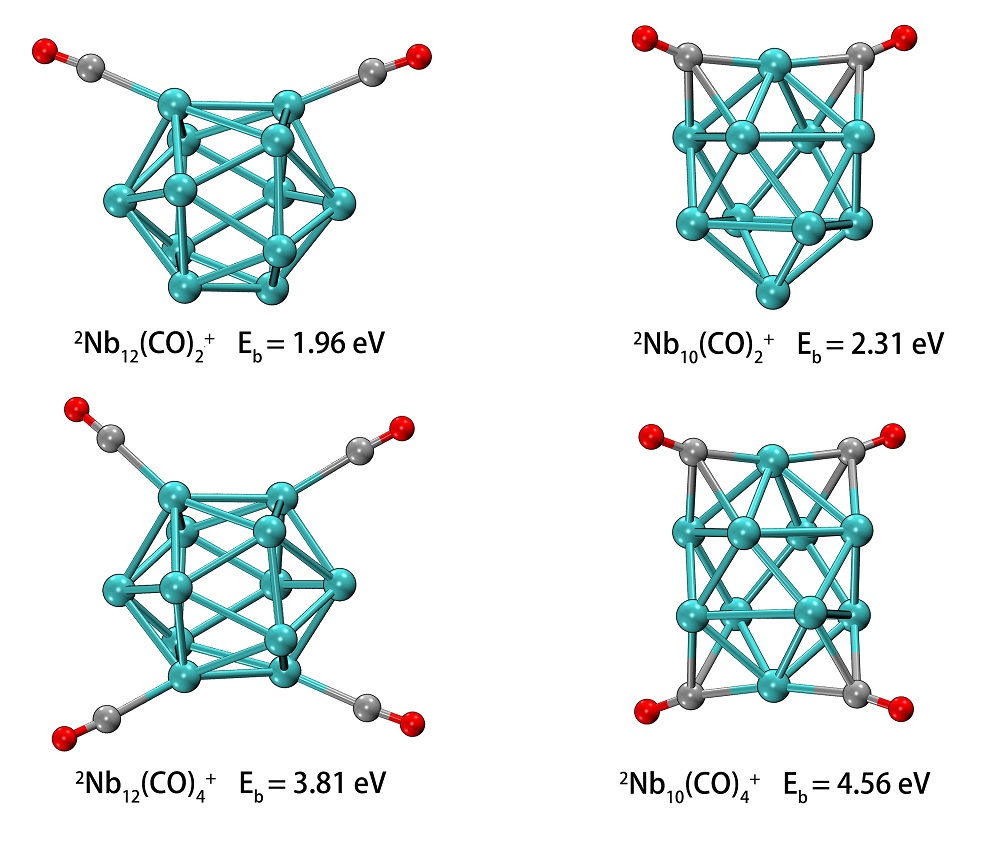
**

Figure S39 | Structure of Nb_12,10_(CO)_2,4_^+^. Optimized lowest-energy structures and total binding energy (E_b_ = E[Nb_12,10_^+^] + E[CO] - E[Nb_12,10_(CO)_2,4_^+^]) of optimized ^2^Nb_12,10_(CO)_2_^+^, ^2^Nb_12,10_(CO)_4_^+^. All the data are calculated at the BPW91/Lanl2TZ(f) level for Nb_n_^+^ and BPW91/6-311G(d) level for CO using the G09 program.

4.2 NPA of Nb_10,12_CO^+^

Table S5| Natural charge and natural electron configuration of Nb_12_^+^, CO, and Nb_12_CO^+^. The data for Nb atoms at the adsorption site are in bold.

| **Atom** | **Nb_12_^+^/CO** | | **Nb_12_CO^+^** | |
| --- | --- | --- | --- | --- |
|  | **Natural charge/e** | **Natural Electron Configuration** | **Natural**  **charge/e** | **Natural Electron Configuration** |
| **(1) Nb** | 0.08 | 5s^0.48^4d^4.21^5p^0.31^5d^0.02^ | 0.07 | 5s^0.47^4d^4.22^5p^0.30^5d^0.02^ |
| **(2) Nb** | 0.07 | 5s^0.49^4d^4.20^5p^0.31^5d^0.02^ | 0.08 | 5s^0.47^4d^4.22^5p^0.30^5d^0.02^ |
| **(3) Nb** | 0.09 | 5s^0.48^4d^4.20^5p^0.31^5d^0.02^ | 0.09 | 5s^0.47^4d^4.22^5p^0.29^5d^0.02^ |
| **(4) Nb** | 0.09 | 5s^0.48^4d^4.18^5p^0.31^5d^0.02^ | 0.08 | 5s^0.47^4d^4.22^5p^0.30^5d^0.02^ |
| **(5) Nb** | 0.10 | 5s^0.48^4d^4.18^5p^0.31^5d^0.02^ | 0.08 | 5s^0.48^4d^4.21^5p^0.29^5d^0.02^ |
| **(6) Nb** | 0.08 | 5s^0.48^4d^4.21^5p^0.31^5d^0.02^ | 0.16 | 5s^0.44^4d^4.16^5p^0.31^5d^0.02^ |
| **(7) Nb** | 0.07 | 5s^0.48^4d^4.21^5p^0.31^5d^0.02^ | 0.18 | 5s^0.46^4d^4.16^5p^0.29^5d^0.02^ |
| **(8) Nb** | 0.07 | 5s^0.49^4d^4.20^5p^0.31^ 5d^0.02^ | 0.16 | 5s^0.47^4d^4.16^5p^0.28^5d^0.02^ |
| **(9) Nb** | 0.09 | 5s^0.48^4d^4.20^5p^0.31^ 5d^0.02^ | **-0.42** | **5s^0.41^4d^4.53^5p^0.54^5d^0.03^** |
| **(10) Nb** | 0.09 | 5s^0.48^4d^4.18^5p^0.31^ 5d^0.02^ | 0.13 | 5s^0.47^4d^4.20^5p^0.27^5d^0.02^ |
| **(11) Nb** | 0.10 | 5s^0.48^4d^4.18^5p^0.31^ 5d^0.02^ | 0.13 | 5s^0.49^4d^4.18^5p^0.27^5d^0.02^ |
| **(12) Nb** | 0.07 | 5s^0.48^4d^4.21^5p^0.31^ 5d^0.02^ | 0.09 | 5s^0.47^4d^4.21^5p^0.30^5d^0.02^ |
| **C** | 0.43 | 2s^1.65^2p^1.87^3s^0.03^3p^0.01^ | 0.53 | 2s^1.24^2p^2.18^3s^0.02^3p^0.02^ |
| **O** | -0.43 | 2s^1.72^2p^4.69^3d^0.01^ | -0.37 | 2s^1.70^2p^4.65^3d^0.01^ |
| **CO** | 0 | \ | 0.16 | \ |

Table S6 | Natural charge and natural electron configuration of Nb_10_^+^, CO, and Nb_10_CO^+^. The data for Nb atoms at the adsorption site are in bold.

| **Atom** | **Nb_10_^+^/CO** | | **Nb_10_CO^+^** | |  |
| --- | --- | --- | --- | --- | --- |
|  | **Natural**  **charge/e** | **Natural Electron Configuration** | **Natural**  **charge/e** | **Natural Electron Configuration** |  |
| **(1) Nb** | 0.10 | 5s^0.48^4d^4.21^5p^0.28^5d^0.02^ | 0.13 | 5s^0.47^4d^4.21^5p^0.26^5d^0.02^ |  |
| **(2) Nb** | 0.10 | 5s^0.48^4d^4.21^5p^0.28^5d^0.02^ | 0.11 | 5s^0.47^4d^4.24^5p^0.25^5d^0.02^ |  |
| **(3) Nb** | 0.10 | 5s^0.48^4d^4.21^5p^0.28^5d^0.02^ | **0.06** | **5s^0.38^4d^4.31^5p^0.34^5d^0.02^** |  |
| **(4) Nb** | 0.10 | 5s^0.48^4d^4.27^5p^0.22^5d^0.02^ | **0.17** | **5s^0.42^4d^4.22^5p^0.26^5d^0.02^** | |
| **(5) Nb** | 0.10 | 5s^0.48^4d^4.21^5p^0.28^5d^0.02^ | **0.05** | **5s^0.37^4d^4.32^5p^0.34^5d^0.02^** | |
| **(6) Nb** | 0.10 | 5s^0.48^4d^4.21^5p^0.28^5d^0.02^ | 0.19 | 5s^0.47^4d^4.16^5p^0.24^5d^0.02^ | |
| **(7) Nb** | 0.10 | 5s^0.48^4d^4.21^5p^0.28^5d^0.02^ | 0.11 | 5s^0.47^4d^4.19^5p^0.30^5d^0.02^ | |
| **(8) Nb** | 0.10 | 5s^0.48^4d^4.21^5p^0.28^5d^0.02^ | 0.08 | 5s^0.47^4d^4.25^5p^0.28^5d^0.02^ | |
| **(9) Nb** | 0.10 | 5s^0.48^4d^4.21^5p^0.28^5d^0.02^ | 0.12 | 5s^0.46^4d^4.21^5p^0.29^5d^0.02^ | |
| **(10) Nb** | 0.11 | 5s^0.48^4d^4.27^5p^0.22^5d^0.02^ | 0.15 | 5s^0.55^4d^4.16^5p^0.22^5d^0.02^ | |
| **C** | 0.43 | 2s^1.65^2p^1.87^3s^0.03^3p^0.01^ | 0.21 | 2s^1.08^2p^2.66^3s^0.01^3p^0.03^ | |
| **O** | -0.43 | 2s^1.72^2p^4.69^3d^0.01^ | -0.38 | 2s^1.68^2p^4.69^3p^0.01^ | |
| **CO** | 0 | \ | -0.17 | \ | |

4.3 Vibration frequency

In general, end-on molecular chemisorption of CO is favoured on most transition metals and alkaline earth metals [52, 53], as verified by the presence of ν(CO) bands in the vibrational spectra; on the other hand, dissociative adsorption of CO was attainable for the early transition metals such as V, Nb and Ta [54]. Among the Nb_n_CO^+^ products, Nb_12_CO^+^ shows its particularity with an end-on adsorption of CO, which accounts for its largest frequency of ν(C-O) at 1876.52 cm^-1^ close to that of the nascent CO molecule at 2133 cm^-1^. The vibration modes are displayed below.

**
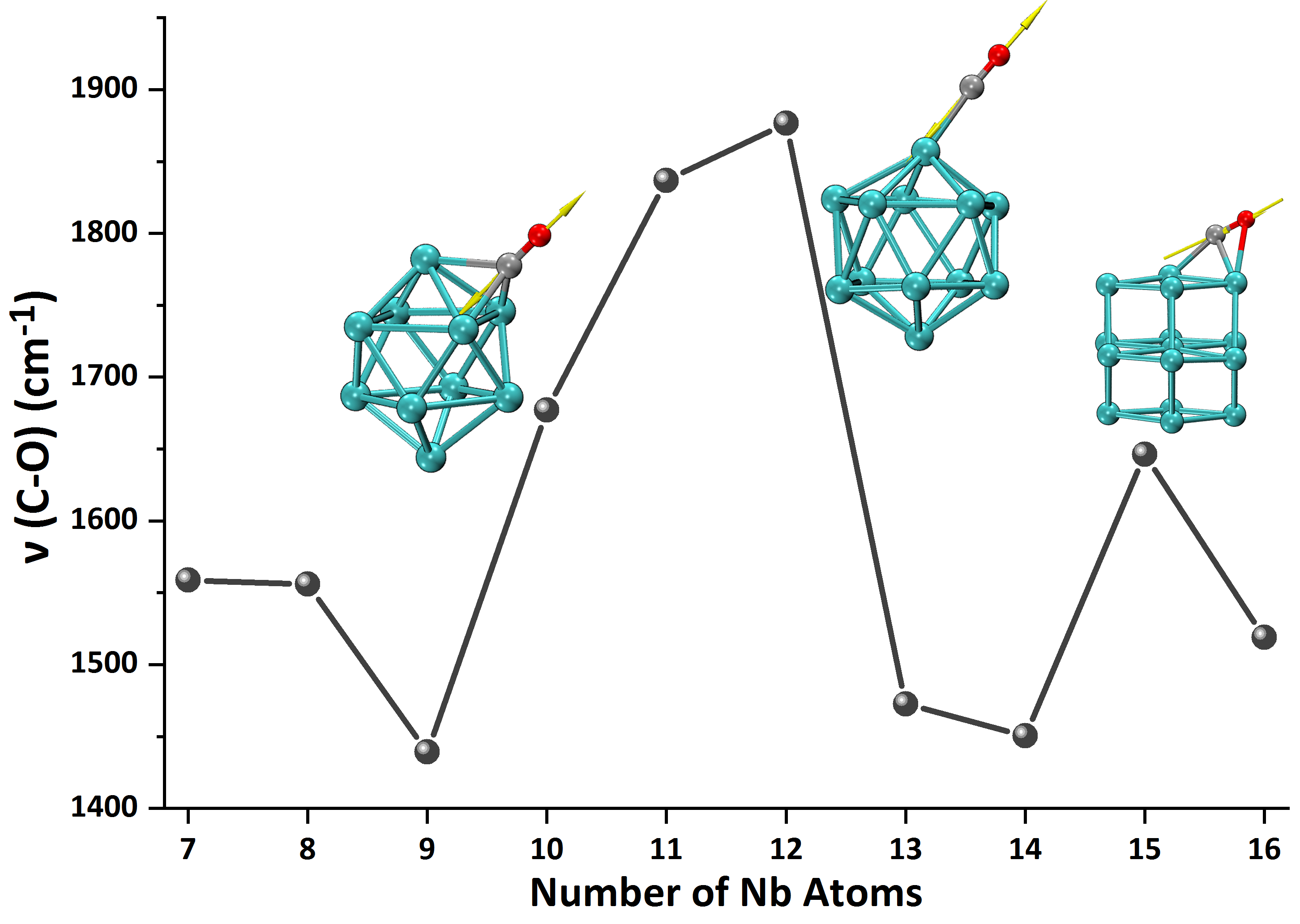
**

**Figure S40 | Vibration frequency of ν (C-O)** **in Nb_n_CO^+^.** Insets show the typical vibrational modes of ν(C-O) in Nb_10,12,15_^+^.

Table S7 | Vibration frequency. DFT-calculated IR-active vibration of ν (C-O) in CO and Nb_7-16_CO^+^ clusters.

|  | **Frequency analysis** |
| --- | --- |
| **Species** | **ν (C-O) (cm^-1^)** |
| **CO** | **2133.10** |
| **Nb_7_CO^+^** | 1558.72 |
| **Nb_8_CO^+^** | 1555.87 |
| **Nb_9_CO^+^** | 1439.20 |
| **Nb_10_CO^+^** | 1677.01 |
| **Nb_11_CO^+^** | 1836.82 |
| **Nb_12_CO^+^** | **1876.52** |
| **Nb_13_CO^+^** | 1472.68 |
| **Nb_14_CO^+^** | 1450.37 |
| **Nb_15_CO^+^** | 1646.41 |
| **Nb_16_CO^+^** | 1518.85 |

4.4 Reaction coordinates


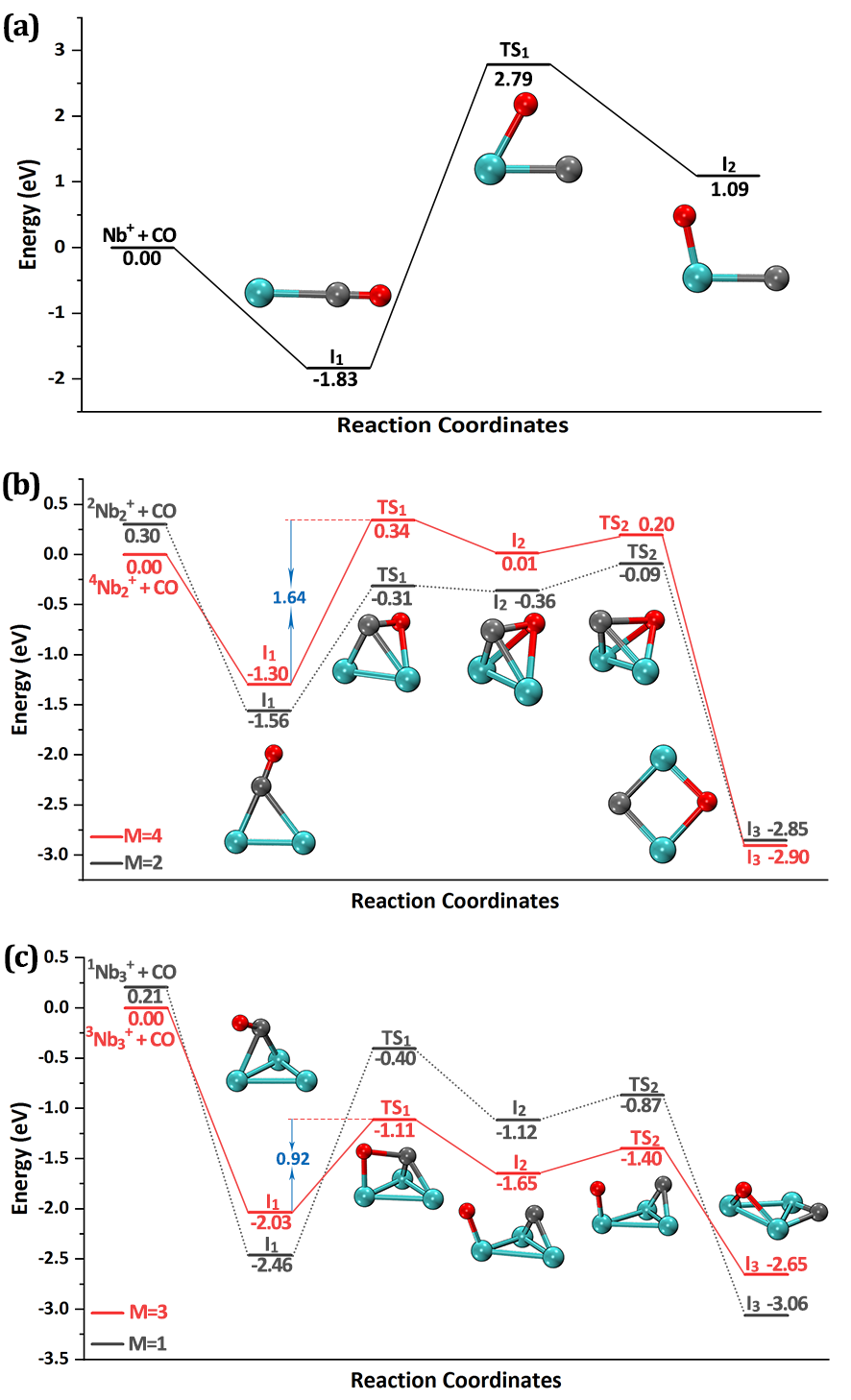


**Figure S41 | Reaction coordinates.** Reaction coordinates of **(a)** Nb^+^ **(b)** Nb_2_^+^ and **(c)** Nb_3_^+^ reacting with CO. All the data are calculated at the BPW91/Lanl2TZ(f) level for Nb_n_^+^ and BPW91/6-311G(d) level for CO using the G09 program. The energy values are relative to the entrance channel, corrected with zero-point vibration energies, and given in eV. Spin multiplicity is marked as pre-superscript.

4.5 PDOS and NAO of Nb_12,10_CO^+^


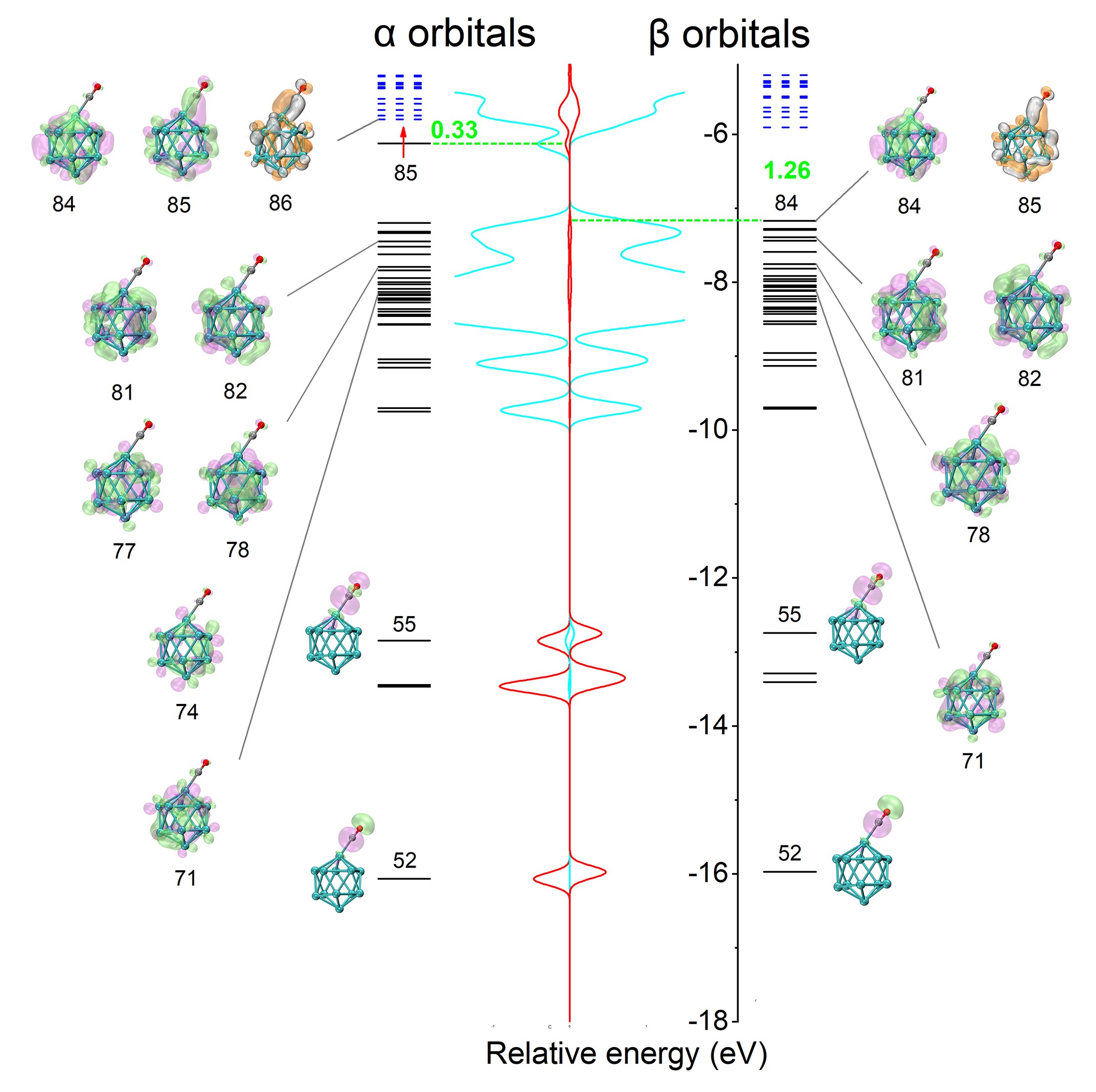


Figure S42 | DOS of Nb_12_CO^+^. The energy level of ^2^Nb_12_CO^+^ together with PDOS of ^2^Nb_12_^+^ (sky blue) and ^1^CO (red). Insets show the interacting orbitals and LUMO orbitals which are marked with two other colors. The isosurface values of orbitals are 0.03.


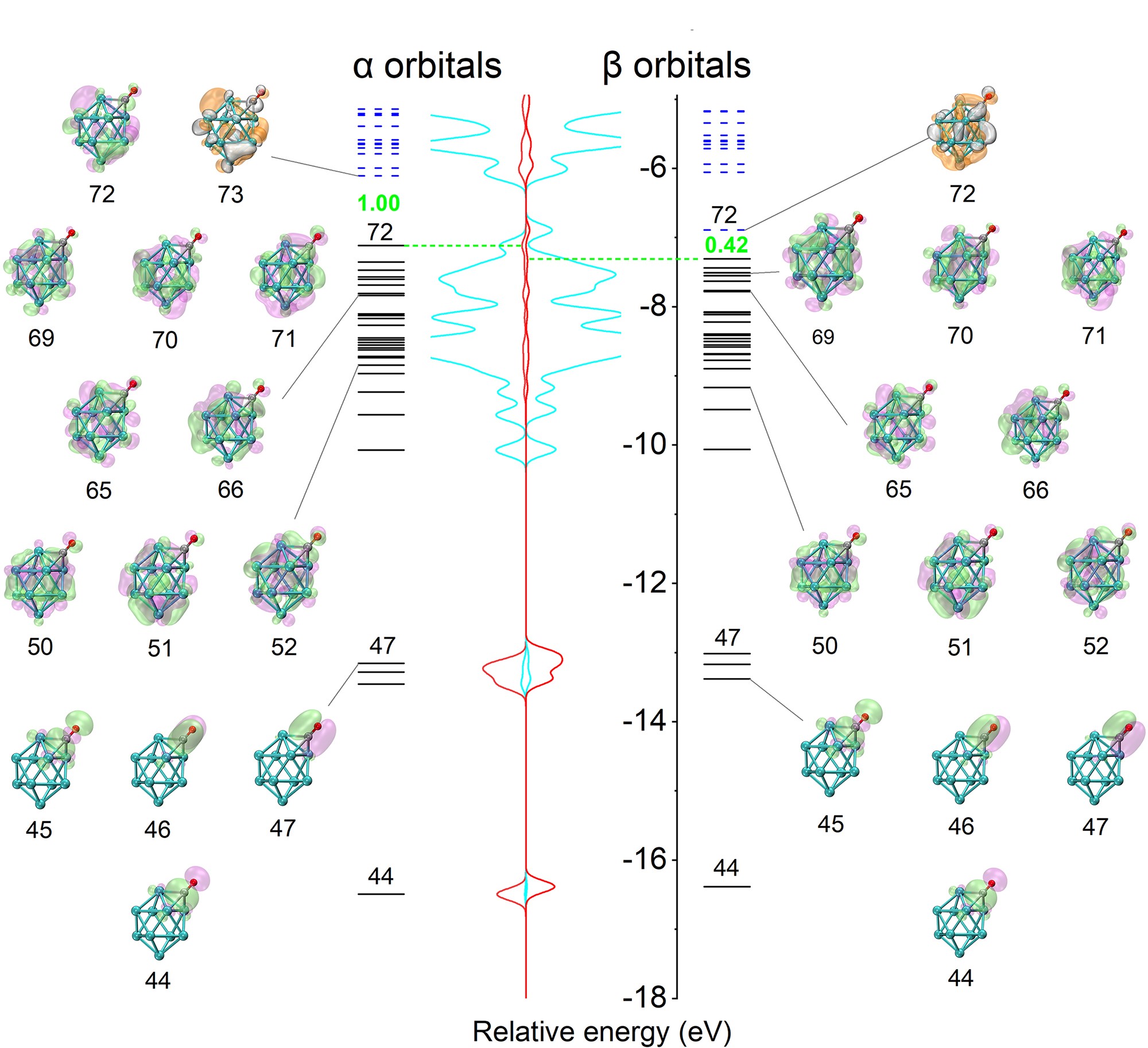


Figure S43 | DOS of Nb_10_CO^+^. Energy level of ^2^Nb_10_CO^+^ together with PDOS of ^2^Nb_10_^+^ (sky blue) and ^1^CO (red). Insets show the interacting orbitals and LUMO orbitals which are marked with two other colors. The isosurface values of orbitals are 0.03.

Table S8 | The atomic contributions to the interacting MO orbitals in ^2^Nb_12_CO^+^ and ^2^Nb_10_CO^+^.

| **Species** | **Number** | **Nb_12/10_^+^** | | **C** | | **O** | |
| --- | --- | --- | --- | --- | --- | --- | --- |
|  |  | Orbital elements | % | Orbital elements | % | Orbital elements | % |
| **^‑2^Nb_12_CO^+^** | 84 | 4d_、_5s _、_5p | 88.11 | 2p | 6.69 | 2p | 4.51 |
|  | 55 | 4d_、_5s _、_5p | 24.57 | 2s_、_2p | 49.82 | 2s_、_2p | 25.01 |
| **^2^Nb_10_CO^+^** | 72 | 4d_、_5s _、_5p | 86.25 | 2s_、_2p | 6.00 | 2p | 7.18 |
|  | 71 | 4d_、_5s _、_5p | 95.30 | 2s_、_2p | 1.79 | 2p | 2.55 |
|  | 66 | 4d_、_5s _、_5p | 94.16 | 2s_、_2p | 2.21 | 2p | 3.29 |
|  | 52 | 4d_、_5s _、_5p | 93.32 | 2s_、_2p | 1.02 | 2p | 5.19 |
|  | 45 | 4d_、_5s _、_5p | 24.57 | 2s_、_2p | 24.46 | 2s_、_2p | 52.97 |

4.6 Energy decomposition analysis

We have conducted energy decomposition analysis (EDA) using the Amsterdam density functional (ADF) program [18, 19], and calculated the interaction energies (ΔE_int_) in Nb_7-16_ CO^+^. The total interaction energies are normally divided into three components (ΔE_int_ = ΔE_pauli_ + ΔE_elstat_ + ΔE_orb_) corresponding to Pauli exclusion (ΔE_pauli_), electrostatic interaction (ΔE_elstat_) and orbital interaction (ΔE_orb_), as shown below. ΔE_elstst_ and ΔE_orb_ are the attraction energies corresponding to electrostatic and orbital interactions, respectively. More specifically, the terms ΔE_orb-α_ and ΔE_orb-β_ refer to α/β orbital interaction energy, respectively, while ΔE_orb-α (1)_ and ΔE_orb-β (2)_ aim at maximum α/β orbital interaction energy items. As shown in Figs 4c and 4d, the ΔE_int_, ΔE_orb_, ΔE_orb-α/β_ (alpha/beta ΔE_orb_), and ΔE_orb-α/β(1)_ (the maximum energy items of ΔE_orb-α/β_) of Nb_12_CO^+^ all have the lowest values among the studied clusters Nb_7-16_CO^+^ (for details see Tables S9-10).


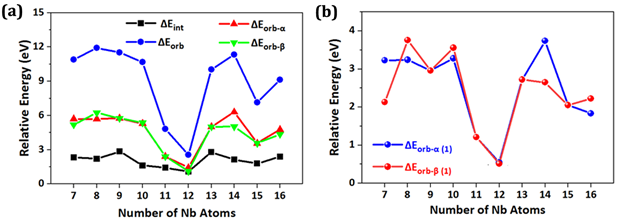


**Figure S44 | Intracluster interactions of Nb_n_CO^+^.** The calculated ΔE_int_ (interaction energy), ΔE_orb_ (overall orbital interaction energy), ΔE_orb-α/β_ (alpha/beta ΔE_orb_), and ΔE_orb-α/β(1)_ (the maximum energy items of ΔE_orb-α/β_) of Nb_12_CO^+^ (c) ΔE_int_, ΔE_orb_, ΔE_orb-α_, ΔE_orb-β_ of Nb_n_CO^+^. (b) The values of ΔE_orb-α (1)_ and ΔE_orb-β (1)_ of Nb_n_CO^+^. The data are calculated at the PW91/TZP level using the ADF program.

Table S9 **|** EDA results for Nb_7-16_CO^+^ at the PW91/TZP level of theory using ADF software package. Energy values are given in eV.

| **Energy term** | **7** | **8** | **9** | **10** | **11** | **12** | **13** | **14** | **15** | **16** |
| --- | --- | --- | --- | --- | --- | --- | --- | --- | --- | --- |
| **ΔE_int_** | -2.3 | -2.2 | -2.9 | -1.6 | -1.4 | -1.1 | -2.8 | -2.2 | -1.8 | -2.4 |
| **ΔE_pauli_** | 18.8 | 20.9 | 19.2 | 18.2 | 8.4 | 4.3 | 16.7 | 20.0 | 12.6 | 15.7 |
| **ΔE_ele_** | -10.3 | -11.2 | -10.6 | -9.2 | -5.0 | -2.8 | -9.5 | -10.9 | -7.3 | -8.9 |
| **ΔE_orb_** | -10.9 | -11.9 | -11.5 | -10.7 | -4.8 | -2.6 | -10.0 | -11.3 | -7.1 | -9.1 |
| **ΔE_orb-α_** | -5.7 | -5.7 | -5.8 | -5.3 | -2.4 | -1.4 | -5.0 | -6.3 | -3.6 | -4.8 |
| **ΔE_orb-β_** | -5.2 | -6.2 |  | -5.4 |  | -1.1 |  | -5.0 |  | -4.4 |
| **ΔE_orb-α (1)_** | -3.2 | -3.2 | -3.0 | -3.3 | -1.2 | -0.6 | -2.7 | -3.7 | -2.1 | -1.8 |
| **ΔE_orb-β (1)_** | -2.1 | -3.8 |  | -3.6 |  | -0.5 |  | -2.7 |  | -2.2 |

Table S10 | EDA results for Nb_12_CO^+^ and Nb_10_CO^+^, calculated at the PW91/TZP level of theory using ADF, taking Nb_12_^+^(/Nb_10_^+^) and CO as interacting fragments. Energies are given in kcal/mol.

| **^2^Nb_12_CO^+^** | | |
| --- | --- | --- |
| **Energy term** | **Assignment** | **kcal/mol** |
| ΔE_int_ |  | -25.0 |
| ΔE_pauli_ |  | 99.7 |
| ΔE_ele_ |  | -65.5 (52.5%) |
| ΔE_orb_ |  | -59.2 (47.5%) |
| ΔE_orb-α (1)_ | π backdonation | -12.7 (25.1%) |
| ΔE_orb-α (2)_ | polarization | -10.8 (18.1%) |
| ΔE_orb-α (3)_ | polarization | -6.3 (10.6%) |
| ΔE_orb-β (1)_ | polarization | -11.8 (19.9%) |
| ΔE_orb-β (2)_ | polarization | -6.4 (9.8%) |
| ΔE_orb_ - rest | - | -11.3 (19.1%) |

| **^2^Nb_10_CO^+^** | |  |
| --- | --- | --- |
| **Energy term** | **Assignment** | **kcal/mol** |
| ΔE_int_ |  | -37.1 |
| ΔE_pauli_ |  | 420.6 |
| ΔE_ele_ |  | -211.9 (46.3%) |
| ΔE_orb_ |  | -246.9 (53.7%) |
| ΔE_orb-α (1)_ | σ donation + π backdonation | -75.5 (30.7%) |
| ΔE_orb-α (2)_ | π backdonation | -27.4 (7.4%) |
| ΔE_orb-α (3)_ | π backdonation + polarization | -9.4 (3.8%) |
| ΔE_orb-β (1)_ | σ donation + π backdonation | -82.0 (33.3%) |
| ΔE_orb-β (2)_ | π backdonation + polarization | -21.5 (8.7%) |
| ΔE_orb-β (3)_ | π backdonation | -9.0 (3.7%) |
| ΔE_orb_ - rest | - | -21.1 (8.6%) |


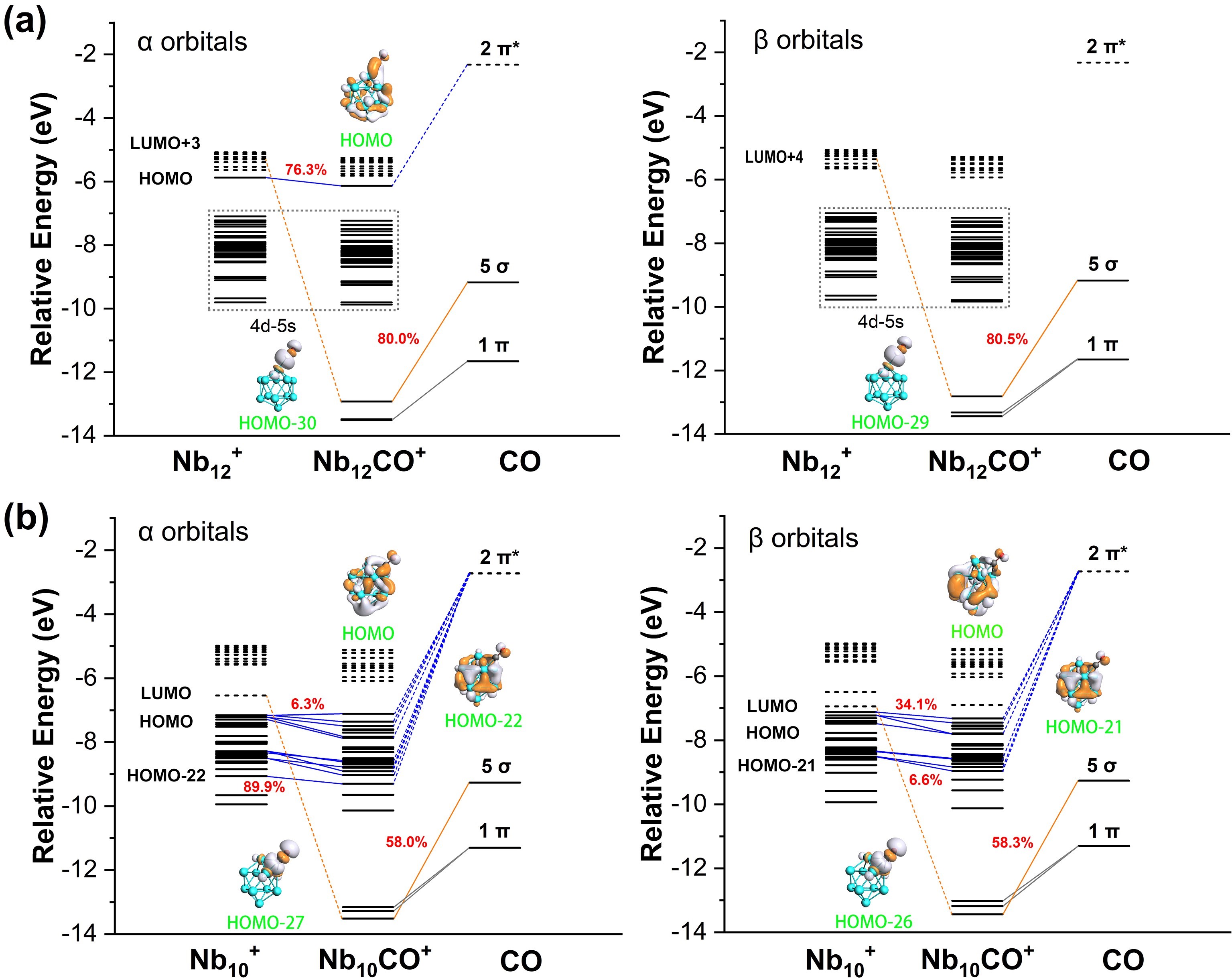


Figure S45 | Kohn-sham **orbital correlation** of Nb_10,12_CO^+^. Kohn-Sham orbital correlation diagrams of ^2^Nb_12_CO^+^ **(a)** and ^2^Nb_10_CO^+^ **(b)** in the α orbitals and β orbitals. Blue lines are for σ donation interaction, orange lines are for π backdonation of two fragments with a percentage, while solid lines are for occupied orbitals and dotted lines are for vacant orbitals. Insets show the main interactional orbitals of complexes.

It is found that the π backdonation in Nb_12_CO^+^ (Nb_12_^+^→CO) is only associated with the α-HOMO of Nb_12_^+^ and vacant 2π* orbital of CO, but not in β orbitals due to the large difference between the energy level of β HOMO of Nb_12_^+^ and the 2π* orbitals of CO. The inner orbitals of Nb_12_^+^ cannot interact with the 2π* orbitals of CO given their much lower energy levels and unmatched orbital shape. In contrast, there are relatively strong π backdonation interactions of both α and β orbitals in Nb_10_CO^+^ due to the matched orbital shape and energy levels.

5. Superatomic nature

To fully unveil the superatomic nature, we have calculated the value of distortion parameter $\eta$ to evaluate the energy levels of Nb_10_^+^ and Nb_12_^+^ in the Clemenger-Nilsson diagram [55], based on the following equation:

$\eta=\frac{2(r_{z}-r_{x})}{r_{z}+r_{x}}$ (s4)

where $r_{z}$ and $r_{x}$ (=$r_{y}$)  refers to the length of the minor and major axes of an oblate-shaped cluster, respectively. As results,

for the lowest energy structure of Nb_10_^+^, $r_{z}$ = 5.13 Å, and $r_{x}$= 3.81 Å, $\eta$ = **0.30**

for the lowest energy structure of Nb_12_^+^, $r_{z}$ = 5.04 Å, and $r_{x}$= 4.34 Å, $\eta$ = **0.15**

for the lowest energy structure of Nb_12_^2+^, r_z_ = 5.07 Å, and r_x_= 4.41 Å, η = **0.14**

**
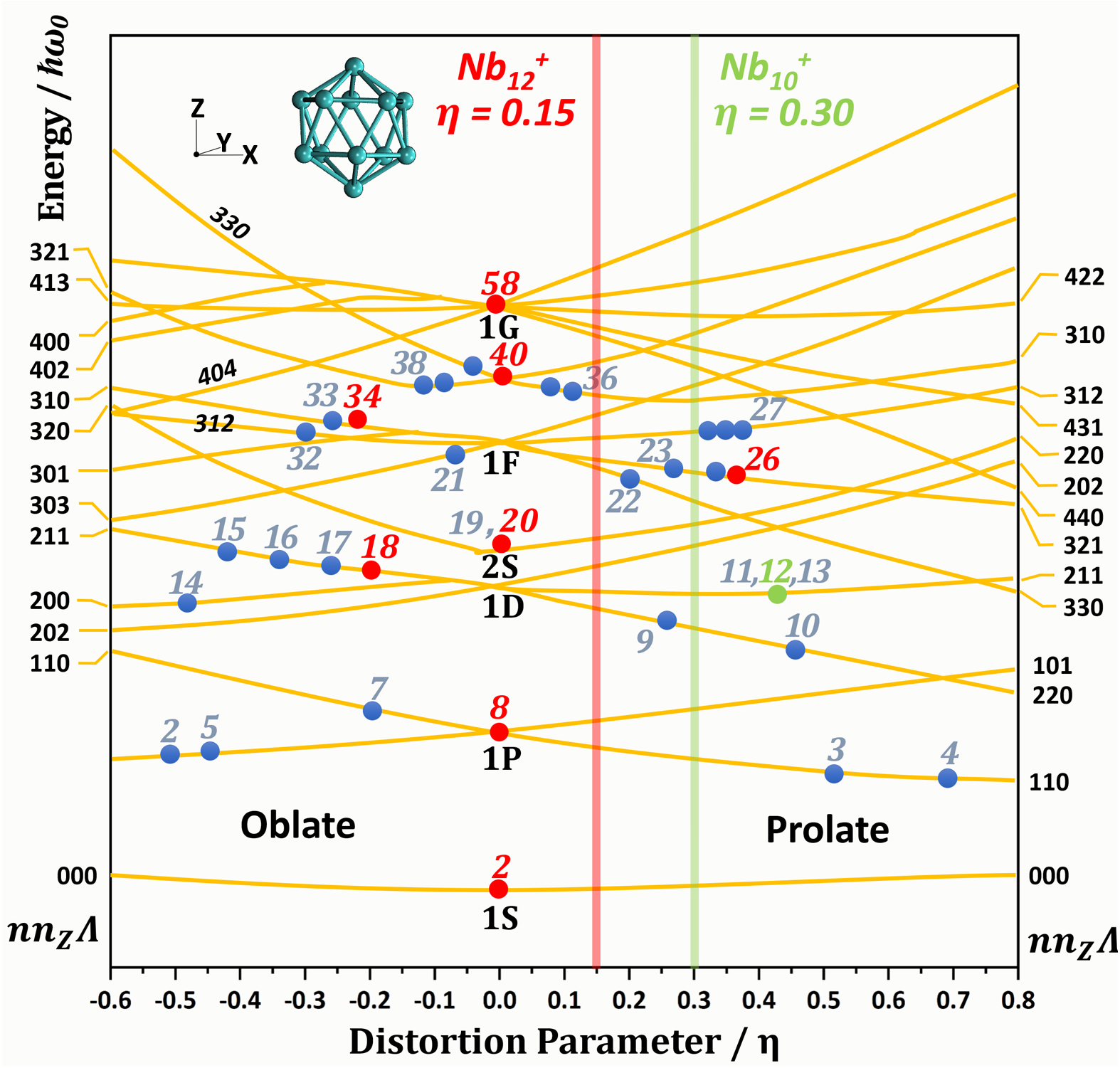
**

**Figure S46 | The effects of well shape on energy levels** as a function of oblate/prolate ellipsoidal distortion, according to the previous results in Ref [55] .

Further, we have estimated the atomic orbital contributions to the superatomic 1S and 2S orbitals, and calculated the orbital delocalization index (ODI) of which the value of a certain orbital (i) is calculated by the following formula, in which $\theta_{A,i}$ means the composition of atom A in orbital i. The smaller ODI value is, the higher delocalization degree of the orbital is. The threshold value range of ODI is at (0, 100].

${ODI}_{i}=0.01\times\sum_{A} {(\theta_{A,i})}^{2}$ (s5)

In this regard, the delocalization degrees of superatomic orbital 1S and 2S in Nb_12_^+^ and Nb_12_^2+^ are at the same level, while the delocalization degrees of superatomic 1S and 2S orbitals in Nb_10_^+^ are relatively lower.

**Table S11** | **Atomic orbital contributions** to the superatomic 1S and 2S orbitals of Nb_12_^+^.

| **Centre** | **Superatomic Orbital 1S**  **Composition** | **Superatomic Orbital 2S**  **Composition** |
| --- | --- | --- |
| **1 (Nb)** | 8.15% | 8.29% |
| **2 (Nb)** | 8.45% | 8.16% |
| **3 (Nb)** | 8.15% | 8.37% |
| **4 (Nb)** | 8.45% | 8.55% |
| **5 (Nb)** | 8.45% | 8.55% |
| **6 (Nb)** | 8.15% | 8.29% |
| **7 (Nb)** | 8.35% | 8.09% |
| **8 (Nb)** | 8.45% | 8.16% |
| **9 (Nb)** | 8.15% | 8.37% |
| **10 (Nb)** | 8.45% | 8.55% |
| **11 (Nb)** | 8.45% | 8.55% |
| **12 (Nb)** | 8.35% | 8.09% |
|  | **Orbital delocalization index=8.34** | **Orbital delocalization index=8.34** |

**Table S12 | Atomic orbital contributions** to the superatomic 1S and 2S orbitals of the divalent cation Nb_12_^2+^.

| **Centre** | **Superatomic Orbital 1S**  **Composition** | **Superatomic Orbital 2S**  **Composition** |
| --- | --- | --- |
| **1 (Nb)** | 8.38% | 8.33% |
| **2 (Nb)** | 8.38% | 8.33% |
| **3 (Nb)** | 8.29% | 8.33% |
| **4 (Nb)** | 8.28% | 8.33% |
| **5 (Nb)** | 8.38% | 8.33% |
| **6 (Nb)** | 8.38% | 8.33% |
| **7 (Nb)** | 8.29% | 8.34% |
| **8 (Nb)** | 8.38% | 8.34% |
| **9 (Nb)** | 8.29% | 8.33% |
| **10 (Nb)** | 8.28% | 8.34% |
| **11 (Nb)** | 8.38% | 8.33% |
| **12 (Nb)** | 8.30% | 8.34% |
|  | **Orbital delocalization index=8.33** | **Orbital delocalization index=8.33** |

**Table S13** | **Atomic orbital contributions** to the superatomic 1S and 2S orbitals of Nb_10_^+^.

| **Centre** | **Superatomic Orbital 1S**  **Composition** | **Superatomic Orbital 2S**  **Composition** |
| --- | --- | --- |
| **1 (Nb)** | 10.35% | 10.22% |
| **2 (Nb)** | 10.35% | 10.22% |
| **3 (Nb)** | 10.35% | 10.22% |
| **4 (Nb)** | 8.61% | 9.11% |
| **5 (Nb)** | 10.35% | 10.22% |
| **6 (Nb)** | 10.35% | 10.22% |
| **7 (Nb)** | 10.35% | 10.22% |
| **8 (Nb)** | 10.35% | 10.22% |
| **9 (Nb)** | 10.35% | 10.22% |
| **10 (Nb)** | 8.61% | 9.11% |
|  | **Orbital delocalization index=10.05** | **Orbital delocalization index=10.02** |

In addition, we have also compared the compositions of the 1S superatomic orbital in Nb_12_^+^, Nb_12_^2+^, and Nb_10_^+^ based on NAO method, as shown below.

| **Table S14 \|** **Composition of the superatomic 1S orbital** in Nb_12_^+^ based on NAO method. | | | | |
| --- | --- | --- | --- | --- |
| **NAO#** | **Centre** | **Label** | **Type** | **Composition** |
| 128 | 4(Nb) | s | Val(5s) | 5.24% |
| 170 | 5(Nb) | s | Val(5s) | 5.24% |
| 380 | 10(Nb) | s | Val(5s) | 5.24% |
| 422 | 11(Nb) | s | Val(5s) | 5.24% |
| 44 | 2(Nb) | s | Val(5s) | 5.217% |
| 296 | 8(Nb) | s | Val(5s) | 5.217% |
| 254 | 7(Nb) | s | Val(5s) | 5.144% |
| 464 | 12(Nb) | s | Val(5s) | 5.144% |
| 86 | 3(Nb) | s | Val(5s) | 5.031% |
| 338 | 9(Nb) | s | Val(5s) | 5.031% |
| 2 | 1(Nb) | s | Val(5s) | 5.027% |
| 212 | 6(Nb) | s | Val(5s) | 5.027% |
| 127 | 4(Nb) | s | Cor(4s) | 2.008% |
| 169 | 5(Nb) | s | Cor(4s) | 2.008% |
| 379 | 10(Nb) | s | Cor(4s) | 2.008% |
| 421 | 11(Nb) | s | Cor(4s) | 2.008% |
| 43 | 2(Nb) | s | Cor(4s) | 1.991% |
| 295 | 8(Nb) | s | Cor(4s) | 1.991% |
| 253 | 7(Nb) | s | Cor(4s) | 1.954% |
| 463 | 12(Nb) | s | Cor(4s) | 1.954% |
| 1 | 1(Nb) | s | Cor(4s) | 1.915% |
| 85 | 3(Nb) | s | Cor(4s) | 1.915% |
| 211 | 6(Nb) | s | Cor(4s) | 1.915% |
| 337 | 9(Nb) | s | Cor(4s) | 1.915% |
| **……** | **…** | **……** | **……** | **……** |
| **Note:** Cor: core NAO; Val: valence shell NAO. | | | | |

| **Table S15 \| Composition of the 2S superatomic orbital in Nb_12_^+^** based on NAO method**.** | | | | |
| --- | --- | --- | --- | --- |
| **NAO#** | **Centre** | **Label** | **Type** | **Composition** |
| 105 | 3(Nb) | dxy | Val(4d) | 5.392% |
| 357 | 9(Nb) | dxy | Val(4d) | 5.392% |
| 198 | 5(Nb) | dx2y2 | Val(4d) | 5.276% |
| 450 | 11(Nb) | dx2y2 | Val(4d) | 5.276% |
| 276 | 7(Nb) | dxz | Val(4d) | 4.129% |
| 486 | 12(Nb) | dxz | Val(4d) | 4.129% |
| 27 | 1(Nb) | dyz | Val(4d) | 3.972% |
| 237 | 6(Nb) | dyz | Val(4d) | 3.972% |
| 69 | 2(Nb) | dyz | Val(4d) | 3.873% |
| 321 | 8(Nb) | dyz | Val(4d) | 3.873% |
| 150 | 4(Nb) | dxz | Val(4d) | 3.771% |
| 402 | 10(Nb) | dxz | Val(4d) | 3.771% |
| 159 | 4(Nb) | dz2 | Val(4d) | 3.168% |
| 411 | 10(Nb) | dz2 | Val(4d) | 3.168% |
| 285 | 7(Nb) | dz2 | Val(4d) | 2.384% |
| 495 | 12(Nb) | dz2 | Val(4d) | 2.384% |
| 117 | 3(Nb) | dz2 | Val(4d) | 2.053% |
| 369 | 9(Nb) | dz2 | Val(4d) | 2.053% |
| 201 | 5(Nb) | dz2 | Val(4d) | 1.88% |
| 453 | 11(Nb) | dz2 | Val(4d) | 1.88% |
| 72 | 2(Nb) | dx2y2 | Val(4d) | 1.742% |
| 324 | 8(Nb) | dx2y2 | Val(4d) | 1.742% |
| 30 | 1(Nb) | dx2y2 | Val(4d) | 1.565% |
| 240 | 6(Nb) | dx2y2 | Val(4d) | 1.565% |
| 21 | 1(Nb) | dxy | Val(4d) | 1.438% |
| 231 | 6(Nb) | dxy | Val(4d) | 1.438% |
| 63 | 2(Nb) | dxy | Val(4d) | 1.297% |
| 315 | 8(Nb) | dxy | Val(4d) | 1.297% |
| **……** | **…** | **……** | **……** | **……** |
| **Note:** Cor: core NAO; Val: valence shell NAO. | | | | |

| **Table S16 \| Composition of the 1S superatomic orbital** in Nb_12_^2+^ based on NAO method. | | | | |
| --- | --- | --- | --- | --- |
| **NAO#** | **Centre** | **Label** | **Type** | **Composition** |
| 2 | 1(Nb) | s | Val(5s) | 4.516% |
| 212 | 6(Nb) | s | Val(5s) | 4.516% |
| 44 | 2(Nb) | s | Val(5s) | 4.511% |
| 170 | 5(Nb) | s | Val(5s) | 4.511% |
| 296 | 8(Nb) | s | Val(5s) | 4.511% |
| 422 | 11(Nb) | s | Val(5s) | 4.511% |
| 464 | 12(Nb) | s | Val(5s) | 4.452% |
| 86 | 3(Nb) | s | Val(5s) | 4.448% |
| 254 | 7(Nb) | s | Val(5s) | 4.448% |
| 338 | 9(Nb) | s | Val(5s) | 4.448% |
| 380 | 10(Nb) | s | Val(5s) | 4.444% |
| 128 | 4(Nb) | s | Val(5s) | 4.439% |
| 1 | 1(Nb) | s | Cor(4s) | 1.6% |
| 211 | 6(Nb) | s | Cor(4s) | 1.6% |
| 295 | 8(Nb) | s | Cor(4s) | 1.6% |
| 43 | 2(Nb) | s | Cor(4s) | 1.598% |
| 169 | 5(Nb) | s | Cor(4s) | 1.598% |
| 421 | 11(Nb) | s | Cor(4s) | 1.598% |
| 253 | 7(Nb) | s | Cor(4s) | 1.59% |
| 463 | 12(Nb) | s | Cor(4s) | 1.59% |
| 85 | 3(Nb) | s | Cor(4s) | 1.588% |
| 337 | 9(Nb) | s | Cor(4s) | 1.588% |
| 127 | 4(Nb) | s | Cor(4s) | 1.585% |
| 379 | 10(Nb) | s | Cor(4s) | 1.585% |
| 237 | 6(Nb) | dyz | Val(4d) | 1.228% |
| 105 | 3(Nb) | dxy | Val(4d) | 1.203% |
| 27 | 1(Nb) | dyz | Val(4d) | 1.177% |
| 357 | 9(Nb) | dxy | Val(4d) | 1.169% |
| 492 | 12(Nb) | dx2y2 | Val(4d) | 1.147% |
| 66 | 2(Nb) | dxz | Val(4d) | 1.141% |
| 282 | 7(Nb) | dx2y2 | Val(4d) | 1.096% |
| 318 | 8(Nb) | dxz | Val(4d) | 1.03% |
| **……** | **…** | **……** | **……** | **……** |
| **Note:** Cor: core NAO; Val: valence shell NAO. | | | | |

| **Table S17 \| Composition of the 2S superatomic orbital** in Nb_12_^2+^ based on NAO method. | | | | |
| --- | --- | --- | --- | --- |
| **NAO#** | **Centre** | **Label** | **Type** | **Composition** |
| 237 | 6(Nb) | dyz | Val(4d) | 4.248% |
| 105 | 3(Nb) | dxy | Val(4d) | 4.235% |
| 357 | 9(Nb) | dxy | Val(4d) | 4.113% |
| 66 | 2(Nb) | dxz | Val(4d) | 4.036% |
| 492 | 12(Nb) | dx2y2 | Val(4d) | 4.032% |
| 27 | 1(Nb) | dyz | Val(4d) | 3.94% |
| 282 | 7(Nb) | dx2y2 | Val(4d) | 3.849% |
| 318 | 8(Nb) | dxz | Val(4d) | 3.367% |
| 192 | 5(Nb) | dxz | Val(4d) | 3.028% |
| 408 | 10(Nb) | dx2y2 | Val(4d) | 2.615% |
| 156 | 4(Nb) | dx2y2 | Val(4d) | 2.277% |
| 447 | 11(Nb) | dyz | Val(4d) | 2.25% |
| 147 | 4(Nb) | dxy | Val(4d) | 2.076% |
| 444 | 11(Nb) | dxz | Val(4d) | 2.039% |
| 399 | 10(Nb) | dxy | Val(4d) | 1.74% |
| 243 | 6(Nb) | dz2 | Val(4d) | 1.281% |
| 195 | 5(Nb) | dyz | Val(4d) | 1.275% |
| 33 | 1(Nb) | dz2 | Val(4d) | 1.268% |
| 285 | 7(Nb) | dz2 | Val(4d) | 1.239% |
| 495 | 12(Nb) | dz2 | Val(4d) | 1.234% |
| 453 | 11(Nb) | dz2 | Val(4d) | 1.217% |
| 369 | 9(Nb) | dz2 | Val(4d) | 1.208% |
| 117 | 3(Nb) | dz2 | Val(4d) | 1.203% |
| 159 | 4(Nb) | dz2 | Val(4d) | 1.199% |
| 411 | 10(Nb) | dz2 | Val(4d) | 1.199% |
| 201 | 5(Nb) | dz2 | Val(4d) | 1.197% |
| 327 | 8(Nb) | dz2 | Val(4d) | 1.197% |
| 75 | 2(Nb) | dz2 | Val(4d) | 1.186% |
| **……** | **…** | **……** | **……** | **……** |
| **Note:** Cor: core NAO; Val: valence shell NAO. | | | | |

| **Table S18 \| Composition of the 1S superatomic orbital** in Nb_10_^+^ based on NAO method. | | | | |
| --- | --- | --- | --- | --- |
| **NAO#** | **Centre** | **Label** | **Type** | **Composition** |
| 159 | 4(Nb) | dz2 | Val(4d) | 6.85% |
| 411 | 10(Nb) | dz2 | Val(4d) | 6.85% |
| 27 | 1(Nb) | dyz | Val(4d) | 2.86% |
| 66 | 2(Nb) | dxz | Val(4d) | 2.86% |
| 111 | 3(Nb) | dyz | Val(4d) | 2.86% |
| 192 | 5(Nb) | dxz | Val(4d) | 2.86% |
| 234 | 6(Nb) | dxz | Val(4d) | 2.86% |
| 279 | 7(Nb) | dyz | Val(4d) | 2.86% |
| 318 | 8(Nb) | dxz | Val(4d) | 2.86% |
| 363 | 9(Nb) | dyz | Val(4d) | 2.86% |
| 30 | 1(Nb) | dx2y2 | Val(4d) | 2.22% |
| 72 | 2(Nb) | dx2y2 | Val(4d) | 2.22% |
| 114 | 3(Nb) | dx2y2 | Val(4d) | 2.22% |
| 198 | 5(Nb) | dx2y2 | Val(4d) | 2.22% |
| 240 | 6(Nb) | dx2y2 | Val(4d) | 2.22% |
| 282 | 7(Nb) | dx2y2 | Val(4d) | 2.22% |
| 324 | 8(Nb) | dx2y2 | Val(4d) | 2.22% |
| 366 | 9(Nb) | dx2y2 | Val(4d) | 2.22% |
| 21 | 1(Nb) | dxy | Val(4d) | 1.75% |
| 63 | 2(Nb) | dxy | Val(4d) | 1.75% |
| 105 | 3(Nb) | dxy | Val(4d) | 1.75% |
| 189 | 5(Nb) | dxy | Val(4d) | 1.75% |
| 231 | 6(Nb) | dxy | Val(4d) | 1.75% |
| 273 | 7(Nb) | dxy | Val(4d) | 1.75% |
| 315 | 8(Nb) | dxy | Val(4d) | 1.75% |
| 357 | 9(Nb) | dxy | Val(4d) | 1.75% |
| 2 | 1(Nb) | s | Val(5s) | 1.03% |
| 44 | 2(Nb) | s | Val(5s) | 1.03% |
| 86 | 3(Nb) | s | Val(5s) | 1.03% |
| 170 | 5(Nb) | s | Val(5s) | 1.03% |
| 212 | 6(Nb) | s | Val(5s) | 1.03% |
| 254 | 7(Nb) | s | Val(5s) | 1.03% |
| 296 | 8(Nb) | s | Val(5s) | 1.03% |
| 338 | 9(Nb) | s | Val(5s) | 1.03% |
| **……** | **…** | **……** | **……** | **……** |
| **Note:** Cor: core NAO; Val: valence shell NAO. | | | | |

| **Table S19 \| Composition of the 2S superatomic orbital** in Nb_10_^+^ based on NAO method. | | | | |
| --- | --- | --- | --- | --- |
| **NAO#** | **Centre** | **Label** | **Type** | **Composition** |
|  |  |  |  |  |
| 2 | 1(Nb) | s | Val(5s) | 5.76% |
| 44 | 2(Nb) | s | Val(5s) | 5.76% |
| 86 | 3(Nb) | s | Val(5s) | 5.76% |
| 170 | 5(Nb) | s | Val(5s) | 5.76% |
| 212 | 6(Nb) | s | Val(5s) | 5.76% |
| 254 | 7(Nb) | s | Val(5s) | 5.76% |
| 296 | 8(Nb) | s | Val(5s) | 5.76% |
| 338 | 9(Nb) | s | Val(5s) | 5.76% |
| 128 | 4(Nb) | s | Val(5s) | 5.28% |
| 380 | 10(Nb) | s | Val(5s) | 5.28% |
| 1 | 1(Nb) | s | Cor(4s) | 1.87% |
| 43 | 2(Nb) | s | Cor(4s) | 1.87% |
| 85 | 3(Nb) | s | Cor(4s) | 1.87% |
| 169 | 5(Nb) | s | Cor(4s) | 1.87% |
| 211 | 6(Nb) | s | Cor(4s) | 1.87% |
| 253 | 7(Nb) | s | Cor(4s) | 1.87% |
| 295 | 8(Nb) | s | Cor(4s) | 1.87% |
| 337 | 9(Nb) | s | Cor(4s) | 1.87% |
| 127 | 4(Nb) | s | Cor(4s) | 1.80% |
| 379 | 10(Nb) | s | Cor(4s) | 1.80% |
| 159 | 4(Nb) | dz2 | Val(4d) | 1.60% |
| 411 | 10(Nb) | dz2 | Val(4d) | 1.60% |
| 27 | 1(Nb) | dyz | Val(4d) | 0.75% |
| 66 | 2(Nb) | dxz | Val(4d) | 0.75% |
| 111 | 3(Nb) | dyz | Val(4d) | 0.75% |
| 192 | 5(Nb) | dxz | Val(4d) | 0.75% |
| 234 | 6(Nb) | dxz | Val(4d) | 0.75% |
| 279 | 7(Nb) | dyz | Val(4d) | 0.75% |
| 318 | 8(Nb) | dxz | Val(4d) | 0.75% |
| 363 | 9(Nb) | dyz | Val(4d) | 0.75% |
| **……** | **…** | **……** | **……** | **……** |
| **Note:** Cor: core NAO; Val: valence shell NAO. | | | | |

References

1. Luo Z, Woodward WH, Smith JC *et al.* Growth kinetics of Al clusters in the gas phase produced by a magnetron-sputtering source. *Int J Mass Spectrom* 2012; **309**: 176-81.

2. Yang M, Wu H, Huang B *et al.* Cluster−π interactions cause size-selective reactivity of cationic silver clusters with acetylene: The distinctive Ag_7_^+^[C_2_H_2_]. *J Phys Chem A* 2019; **123**: 6921-6.

3. Frisch MJ, Trucks GW, Schlegel HB *et al.* Gaussian 09 Rev. E.01. Gaussian 09 Rev. E.01, Wallingford CT; 2009.

4. Oganov AR and Glass CW. Crystal structure prediction using ab initio evolutionary techniques: Principles and applications. *J Chem Phys* 2006; **124**: 244704.

5. Kresse G and Furthmüller J. Efficient iterative schemes for ab initio total-energy calculations using a plane-wave basis set. *Phys Rev B* 1996; **54**: 11169-86.

6. Hales DA, Lian L and Armentrout PB. Collision-induced dissociation of Nb_n_^+^ (n = 2-11): Bond energies and dissociation pathways. *Int J Mass Spectrom Ion Processes* 1990; **102**: 269-301.

7. Holmgren L, Andersson M and Rosén A. CO reactivity of small transition-metal clusters: Ni_n_ and Nb_n_. *Surf Sci* 1995; **331-333**: 231-6.

8. Berg C, Schindler T, Niedner‐Schatteburg G *et al.* Reactions of simple hydrocarbons with Nb_n_^+^: Chemisorption and physisorption on ionized niobium clusters. *J Chem Phys* 1995; **102**: 4870-84.

9. Pham Vu N, Vu Thi N, Truong Ba T *et al.* Electronic structures, vibrational and thermochemical properties of neutral and charged niobium clusters Nb_n_, n=7-12. *J Phys Chem A* 2011; **115**: 3523-35.

10. Nhat PV and Nguyen MT. Structures, spectra, and energies of niobium clusters from Nb_13_ to Nb_20_. *J Phys Chem A* 2012; **116**: 7405-18.

11. Hay PJ and Wadt WR. Ab initio effective core potentials for molecular calculations. Potentials for the transition metal atoms Sc to Hg. . *J Chem Phys* 1985; **82**: 270-83.

12. Becke AD. Density-functional exchange-energy approximation with correct asymptotic behavior. *Phys Rev A* 1988; **38**: 3098-100.

13. Perdew JP and Wang Y. Accurate and simple analytic representation of the electron-gas correlation energy. *Phys Rev B* 1992; **45**: 13244-9.

14. Reed AE, Weinstock RB and Weinhold F. Natural population analysis. *J Chem Phys* 1985; **83**: 735-46.

15. Glendening ED, Landis CR and Weinhold F. Nbo 6.0: Natural bond orbital analysis program. *J Comput Chem* 2013; **34**: 1429-37.

16. Lu T and Chen F. Multiwfn: A multifunctional wavefunction analyzer. *J Comput Chem* 2012; **33**: 580-92.

17. Humphrey W, Dalke A and Schulten K. VMD: Visual molecular dynamics. *J Mol Graph* 1996; **14**: 33-8.

18. Baerends EJ, Ziegler T, Autschbach J *et al.* *ADF2018, SCM, Theoretical chemistry*.

19. te Velde G, Bickelhaupt FM, Baerends EJ *et al.* Chemistry with ADF. *J Comput Chem* 2001; **22**: 931-67.

20. Elser V and Haddon RC. Icosahedral C_60_: An aromatic molecule with a vanishingly small ring current magnetic susceptibility. *Nature* 1987; **325**: 792-4.

21. Schleyer PVR, Maerker C, Dransfeld A *et al.* Nucleus-independent chemical shifts: A simple and efficient aromaticity probe. *J Am Chem Soc* 1996; **118**: 6317-8.

22. Chen ZF, Corminboeuf C, Heine T *et al.* Do all-metal antiaromatic clusters exist? *J Am Chem Soc* 2003; **125**: 13930-1.

23. Chen Z, Wannere CS, Corminboeuf C *et al.* Nucleus-independent chemical shifts (NICS) as an aromaticity criterion. *Chem Rev* 2005; **105**: 3842-88.

24. Jusélius J, Sundholm D and Gauss J. Calculation of current densities using gauge-including atomic orbitals. *J Chem Phys* 2004; **121**: 3952-63.

25. Fliegl H, Taubert S, Lehtonen O *et al.* The gauge including magnetically induced current method. *Phys Chem Chem Phys* 2011; **13**: 20500-18.

26. Taubert S, Sundholm D and Jusélius J. Calculation of spin-current densities using gauge-including atomic orbitals. *J Chem Phys* 2011; **134**: 054123.

27. VandeVondele J, Krack M, Mohamed F *et al.* Quickstep: Fast and accurate density functional calculations using a mixed gaussian and plane waves approach. *Comput Phys Commun* 2005; **167**: 103-28.

28. Wolinski K, Hinton JF and Pulay P. Efficient implementation of the gauge-independent atomic orbital method for NMR chemical shift calculations. *J Am Chem Soc* 1990; **112**: 8251-60.

29. Ditchfield R. Self-consistent perturbation theory of diamagnetism. *Mol Phys* 1974; **27**: 789-807.

30. Perdew JP. Erratum: Density-functional approximation for the correlation energy of the inhomogeneous electron gas. *Phys Rev B* 1986; **34**: 7406-.

31. Lee C, Yang W and Parr RG. Development of the Colle-Salvetti correlation-energy formula into a functional of the electron density. *Phys Rev B* 1988; **37**: 785-9.

32. Knickelbein MB and Yang S. Photoionization studies of niobium clusters: Ionization potentials for Nb_2_–Nb_76_. *J Chem Phys* 1990; **93**: 5760-7.

33. Böhme DK. Experimental studies of positive ion chemistry with flow-tube mass spectrometry: Birth, evolution, and achievements in the 20^th^ century. *Int J Mass Spectrom* 2000; **200**: 97-136.

34. Ferguson EE, Fehsenfeld FC and Schmeltekopf AL. Flowing afterglow measurements of ion-neutral reactions. In: Bates, DR and Estermann, I (eds.). *Advances in Atomic and Molecular Physics*: Academic Press; 1969. 1-56.

35. Luo Z and Khanna S. *Metal clusters and their reactivity*. Springer Nature Singapore Pte Ltd.: Springer; 2020.

36. Balteanu I, Achatz U, Balaj OP *et al.* The effect of charge upon CO-adsorption by ionic group 5 and group 9 transition metal clusters. *Int J Mass Spectrom* 2003; **229**: 61-5.

37. Yang M, Zhang H, Jia Y *et al.* Charge-sensitive cluster-pi interactions cause altered reactivity of Al_n_^+/-,0^ clusters with benzene: Enhanced stability of Al_13_^+^Bz. *J Phys Chem A* 2020; **124**: 4087-94.

38. Cox DM, Reichmann KC, Trevor DJ *et al.* CO chemisorption on free gas phase metal clusters. *J Chem Phys* 1988; **88**: 111-9.

39. Macchi P and Sironi A. Chemical bonding in transition metal carbonyl clusters: Complementary analysis of theoretical and eexperimental electron densities. *Coord Chem Rev* 2003; **238**: 383-412.

40. Veldeman N, Lievens P and Andersson M. Size-dependent carbon monoxide adsorption on neutral gold clusters. *J Phys Chem A* 2005; **109**: 11793-801.

41. Fielicke A, von Helden G, Meijer G *et al.* Gold cluster carbonyls: Saturated adsorption of CO on gold cluster cations, vibrational spectroscopy, and implications for their structures. *J Am Chem Soc* 2005; **127**: 8416-23.

42. Fielicke A, von Helden G, Meijer G *et al.* Size and charge effects on the binding of CO to late transition metal clusters. *J Chem Phys* 2006; **124**: 194305.

43. Glatzel P, Singh J, Kvashnina KO *et al.* In situ characterization of the 5d density of states of Pt nanoparticles upon adsorption of CO. *J Am Chem Soc* 2010; **132**: 2555-7.

44. Kerpal C, Harding DJ, Meijer G *et al.* CO adsorption on neutral iridium clusters. *Eur Phys J D* 2011; **63**: 231-4.

45. McNary CP and Armentrout PB. Iron cluster-CO bond energies from the kinetic energy dependence of the Fe_n_^+^ (n = 4-17) + CO association reactions. *Phys Chem Chem Phys* 2014; **16**: 26467-77.

46. Deng G, Lei S, Pan S *et al.* Filling a gap: The coordinatively saturated group 4 carbonyl complexes TM(CO)_8_ (TM=Zr, Hf) and Ti(CO)_7_. *Chemistry* 2020; **26**: 10487-500.

47. Unkrig W, Schmitt M, Kratzert D *et al.* Synthesis and characterization of crystalline niobium and tantalum carbonyl complexes at room temperature. *Nat Chem* 2020; **12**: 647-53.

48. Yoon B, Hakkinen H, Landman U *et al.* Charging effects on bonding and catalyzed oxidation of CO on Au_8_ clusters on MgO. *Science* 2005; **307**: 403-7.

49. Abdulhussein HA, Ferrari P, Vanbuel J *et al.* Altering CO binding on gold cluster cations by Pd-doping. *Nanoscale* 2019; **11**: 16130-41.

50. Ferrari P, Libeert G, Tam NM *et al.* Interaction of carbon monoxide with doped metal clusters. *CrystEngComm* 2020; **22**: 4807-15.

51. Frenking G, Fernández I, Holzmann N *et al.* Metal–CO bonding in mononuclear transition metal carbonyl complexes. *JACS Au* 2021; **1**: 623-45.

52. Fielicke A, Gruene P, Meijer G *et al.* The adsorption of CO on transition metal clusters: A case study of cluster surface chemistry. *Surf Sci* 2009; **603**: 1427-33.

53. Wu X, Zhao L, Jin J *et al.* Observation of alkaline earth complexes M(CO)_8_ (M = Ca, Sr, or Ba) that mimic transition metals. *Science* 2019; **361**: 912-6.

54. Felicio-Sousa P, Andriani KF and Da Silva JLF. Ab initio investigation of the role of the d-states occupation on the adsorption properties of H_2_, CO, CH_4_ and CH_3_OH on the Fe_13_, Co_13_, Ni_13_ and Cu_13_ clusters dagger. *Phys Chem Chem Phys* 2021; **23**: 8739-51.

55. Clemenger K. Ellipsoidal shell structure in free-electron metal clusters. *Phys Rev B* 1985; **32**: 1359-62.
